# Lipid biomarkers for the prediction of type 2 diabetes risk, an umbrella review and updated meta-analyses of prospective observational studies

**DOI:** 10.3389/fendo.2026.1784917

**Published:** 2026-05-08

**Authors:** Xuxin Chen, Zishan Jin, Li Gong, ShunSeng Keng, Yifan Zhang, Yilu Zhang, Caiyi Long, Yueheng Pu, Mengnan Zhang, Sicheng Wang, Boxun Zhang

**Affiliations:** 1 Department of Endocrinology, Hospital of Chengdu University of Traditional Chinese Medicine, Chengdu, China; 2 Beijing University of Chinese Medicine, Beijing, China; 3 Institute of Metabolic Disease, Guang’anmen Hospital, China Academy of Chinese Medical Sciences, Beijing, China; 4 The Second Clinical College, The Second Affiliated Hospital of Qiqihar Medical University, Qiqihar, China; 5 The Second Clinical College, Chongqing Medical University, Chongqing, China

**Keywords:** lipid biomarkers, meta-analysis, prospective study, type 2 diabetes, umbrella review

## Abstract

**Background:**

Although multiple prospective studies have examined associations between lipid metabolism disorders and the risk of type 2 diabetes (T2D), a systematic review and data updates are still lacking. Nowadays, some noval lipid metabolism biomarkers have received increasing attention, but who is the most predictive biomarkers of T2D? this study aims to conduct the in-depth exploration.

**Methods:**

We conducted an umbrella review of meta-analyses of prospective cohort studies by searching PubMed, Web of Science, Cochrane Library, and Embase from inception to 14 February 2025. Methodological quality was assessed using the AMSTAR 2, and evidence strength was evaluated using predefined credibility criteria. The study protocol was registered in PROSPERO (CRD420250649341).

**Results:**

A total of 13 meta-analyses, 133 original articles (including 39 newly included articles) and 1, 226, 322 participants were included. The results of meta-analysis showed significant positive correlation between several lipid metabolism parameters and the risk of T2D. The strongest associations were observed for the hypertriglyceridemic waist (HTW) phenotype [Relative risk (RR) = 3.54, 95% CI: 1.92, 6.53]. Significant positive correlations were also confirmed for the lipid accumulation product (LAP) (RR = 2.94, 95% CI: 2.31, 3.73), the triglyceride glucose (TyG) index (RR = 2.51, 95% CI: 2.13, 2.95), the atherogenic index of plasma (AIP) (RR = 1.97, 95% CI: 1.54, 2.52), the visceral adiposity index (VAI) (RR = 1.77, 95% CI: 1.61, 1.94), the triglyceride to high-density lipoprotein cholesterol (TG/HDL-C) ratio (RR = 1.51, 95% CI: 1.36, 1.69), and non-high-density lipoprotein cholesterol (non-HDL-C) (RR = 1.27, 95% CI: 1.07, 1.52). In contrast, lipoprotein(a) [Lp(a)] showed an inverse association (RR = 0.73, 95% CI: 0.56, 0.96). Dose-response meta-analysis suggested that there was a significant linear relationship between the TG/HDL-C ratio and T2D risk (*P*_for nonlinearity_ = 0.2502), whereas significant nonlinear associations were observed for the VAI, LAP, TyG index, and AIP (*P*_for nonlinearity_ < 0.05). Furthermore, the analysis results of fatty acids revealed positive associations between palmitic acid (C16:0; RR = 1.19, 95% CI: 1.07, 1.33), palmitoleic acid (C16:1n-7; RR = 1.33, 95% CI: 1.19, 1.49), dihomo-γ-linolenic acid (DGLA, C20:3n-6; RR = 1.36, 95% CI: 1.21, 1.53) and the risk of T2D. In contrast, negative associations were found between pentadecanoic acid (C15:0; RR = 0.82, 95% CI: 0.73, 0.92), arachidic acid (C20:0; RR = 0.78, 95% CI: 0.68, 0.90), linoleic acid (LA, C18:2n-6; RR = 0.80, 95% CI: 0.74, 0.87) and the occurrence of T2D.

**Conclusion:**

This systematic review supportsed the predictive value of multiple lipid biomarkers for the risk of T2D, especially some composite indicators such as the HTW, LAP, TyG index, AIP, VAI and TG/HDL-C ratio, but larger-scale and longer-term prospective studies are warranted to validate these associations.

## Graphical Abstract

**Figure:**
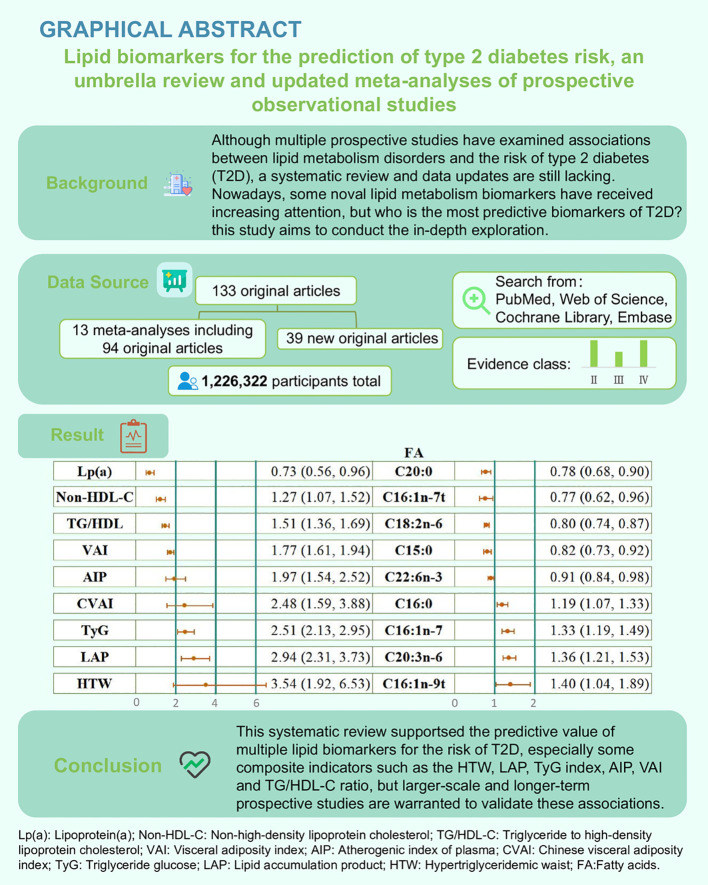

## Introduction

The global epidemic of T2D has become a major public health challenge. Preventing the progression of individuals at high risk of diabetes to T2D represents a crucial strategy for curbing the high incidence of this disease. The glucose-lipid metabolism interplay is a core physiological process essential for maintaining systemic energy homeostasis, and the dysregulation may directly drive the development of T2D.

Mechanistically, elevated circulating triglycerides (TG) promote the deposition of free fatty acids (FFAs) in the pancreas, where pancreatic lipotoxicity can induce endoplasmic reticulum stress and mitochondrial dysfunction, directly impairing the insulin secretion (1). Besides, excessive FFAs may also accumulate in insulin target organs such as the liver, muscle and adipose tissue, thereby activating inflammatory signaling pathways and inhibiting insulin receptor substrate phosphorylation (2), and this inhibition disrupts the downstream PI3K-Akt signaling pathway, leading to reduced glucose uptake and increased hepatic glucose production, which collectively promote systemic insulin resistance and T2D onset. Concurrently, cholesterol metabolism can also significantly regulate the glucose metabolism, in which elevated circulating low-density lipoprotein (LDL) can directly impair the islet β-cell function and inhibit insulin secretion, whereas the high-density lipoprotein (HDL) can exert protective effects on islet β-cells (3). A meta-analysis published a decade ago provided the clinical evidence stating that abnormal lipid parameters possess predictive value for the development of T2D, underscoring the potential impact of lipid metabolism disorders on glucose metabolism.

Over the past decade, in addition to the traditional lipid profile (e.g. total cholesterol (TC), TG, LDL-C, HDL-C), indicators such as non-HDL-C levels and Lp(a) have gained increasing clinical attention. Non-HDL-C, which represents the total concentration of atherogenic lipoprotein particles (4), exhibits superior predictive value for T2D risk, and the elevation of non-HDL-C increases the risk of developing T2D by 1.16-fold in individuals without baseline T2D (5). In addition, Lp(a), a unique lipoprotein formed by the covalent binding of apolipoprotein(a) to an LDL-like particle via a disulfide bond (6), serves not only as an independent risk factor for the atherosclerotic cardiovascular disease but also shows a complex association with the T2D risk, in which very low Lp(a) levels have been shown to increase the T2D risk by 1.28-fold in individuals without baseline T2D, whereas high Lp(a) levels appear inversely associated with the disease (7). Additionally, other lipid-related indicators such as low APOA1 levels (8), high APOB levels (9), elevated concentration of large very-low-density lipoprotein (VLDL) particles, and increased mean particle size of VLDL have also demonstrated predictive value T2D onset (10). In addition, with the advancement of metabolomics technology, fatty acids (FAs)-the fundamental constituents of human lipids-have garnered increasing attention. Clinical evidence indicates that odd-chain saturated fatty acids (SFAs) (C15:0, C17:0) are associated with lowered T2D risk, while even-chain SFAs (e.g., C14:0, C16:0) increase the risk. Furthermore, among polyunsaturated fatty acids (PUFAs), C18:2n-6 and the trans-C16:1n-7 are associated with the reduced T2D risk, whilst imbalances in these FA proportions may promote the disease development through mechanisms such as chronic inflammation.

In addition to individual lipid parameters, composite indices derived from lipid metabolism indicators also exhibit strong predictive potential. The TG/HDL-C ratio which represents the ratio of TG to HDL-C, its elevation signifies an increased risk of dyslipidemia and cardiovascular disease. The VAI which incorporates TG, HDL-C, body mass index (BMI) and waist circumference (WC), quantifies the dysfunctional visceral adipose tissue and the profile/status of lipid metabolism. The abovementioned formulas can also reflect the severity of insulin resistance to some extent (11). The LAP, incorporating TG and WC, quantifies the accumulation of dysfunctional visceral adipose tissue and reflects associated metabolic disturbances. The TyG index integrates fasting TG and blood glucose levels and serves as an independent marker of T2D risk, while the HTW further incorporates the TG and WC, which highlight the significant contribution of central obesity to the metabolic abnormalities. Furthermore, cholesterol level are also crucial for the metabolic status assessment. The AIP which calculated based on TC and HDL-C levels, not only evaluates the risk of atherosclerosis but also demonstrates predictive value for glucose metabolism disorders (the calculation formula for the above composite parameters are detailed in **Supplementary Table 1**).

As mentioned above, numerous prospective cohort-based clinical studies have consistently reported significant correlation between different lipid metabolism indicators and T2D incidence, and several meta-analyses have also investigated the relationship between a single lipid metabolism parameter and the T2D risk. However, a comprehensive analysis focusing on the clinical evidence of lipid metabolism and the T2D risk remains lacking. Therefore, this review aims: (1) systematically retrieve and summarize meta-analyses of prospective cohort studies investigating the relationship between lipid metabolism indicators-including individual lipid parameters (e.g. TC, TG, LDL-C, HDL-C, non-HDL-C, Lp(a), etc.), composite lipid metabolism indices (e.g. TG/HDL-C, VAI, LAP, TyG index, AIP, etc.), and FAs (Saturated and unsaturated)-and T2D risk; (2) explore potential dose-response relationships between the above parameters and T2D risk; (3) conduct a methodological quality assessment (using the AMSTAR-2 tool) of existing meta-analyses, and construct a comprehensive clinical evidence landscape for the “lipid biomarker-T2D risk” relationship, aiming to provide stronger clinical evidence for T2D risk warning and the individualized intervention.

## Methods

This review was conducted and reported according to the Preferred Reporting Items for Systematic Reviews and Meta Analyses (PRISMA) statement (12), and the protocol was prospectively registered with PROSPERO(CRD420250649341).

### Search strategy and study selection

Two investigators (X-XC and Z-SJ) independently searched PubMed, Web of Science, Cochrane Library and Embase databases from inception to 14 February, 2025 using a search strategy to identify meta-analyses of prospective cohort studies. The literature search strategies was provided in **Supplementary Table 2**. Besides, we also searched for individual prospective cohort studies to update the meta-analyses (strategy in **Supplementary Tables 3**–**S11**). All identified publications went through a 3-step parallel review of title, abstract, and full text based on predefined inclusion and exclusion criteria, and any discrepancies were resolved by consensus.

### Inclusion and exclusion criteria

Systematic reviews with meta-analyses were included if they met the following criteria: they synthesized results from studies conducted in general adult populations (participants ≥ 18 years old) without diagnosed T2D at the baseline; they assessed the incidence of T2D as the primary outcome; and they reported quantitative associations [adjusted hazard ratio (HR), relative risk (RR), or odds ratio (OR)] between the incidence rate of T2D and at least one of the lipid metabolism indicators; and meta-analyses were conducted based on prospective cohort studies. On the contrary, some studies were excluded due to the following reasons: they only focused on children, adolescents, pregnant women or other special groups of people; systematic reviews that did not include a quantitative meta-analysis component; the results of meta-analyses were based on retrospective data, cross-sectional study or animal studies, instead of the prospective follow-up cohort; and the language of publication is non-English. In addition, newly retrieved original studies were also reviewed whether met the inclusion and exclusion criteria, and the study population, study design, outcome measures are the same as those for the meta-analysis.

### Data extraction

For the meta-analyses, the name of the lead author, publication year, study design, number of participants, follow up period, summary risk estimates, heterogeneity (*I*^2^), and the publication bias were extracted and recorded; for the original research, the name of the lead author, publication year, study design, number of participants, follow up period and the reported risk estimate were recorded. Two investigators (X-XC and Z-SJ) extracted data independently using a predesigned data extraction form, and disagreements were resolved by consensus.

### Data synthesis and statistical analysis

We extracted primary studies from eligible meta-analyses, deduplicated them, and then supplemented with newly published studies (especially those after the latest meta-analysis) to generate a complete, deduplicated list of original studies based on current literature. Subsequently, an updated meta-analysis was performed when a newly published study meeting the eligibility criteria became available, provided sufficient data could be obtained. Heterogeneity was evaluated using Cochran’ s *Q* test and the *I^2^* statistic, and 95% prediction intervals were calculated to interpret the dispersion of true effects. Where significant heterogeneity was present (*I^2^* ≥ 50%), the DerSimonian and Laird random-effects model was applied. Where appropriate, publication bias and small-study effects were assessed by a combination of graphical and statistical methods. For meta-analysis including 10 studies or more, funnel plot symmetry was visually inspected, and Begg’ s and Egger’ s tests were conducted. Sensitivity analyses were performed to examine the stability of the reported associations. For continuous exposure biomarkers with at least three quantitative categories of exposure reported by studies, a random-effects dose–response meta-analysis was conducted using the generalized least-squares regression trend estimation method based on the natural logarithm of the logRRs across these categories. Where sufficient data permitted, potential non-linear relationships were evaluated with restricted cubic splines (typically placed at seven knots). Non-linearity was statistically tested using a Wald-type test on the coefficient of the second spline term. All analyses were conducted using Stata (version 15; StataCorp, College Station, TX, USA). Separately, an excess significance test was conducted using the *χ^2^* test to investigate whether the observed number of studies with nominally significant results (O;”positive” studies, *P* < 0.05) differed from the expected number of significant results (E). An excess significance test result of *P* < 0.05 suggests the presence of excess significance bias. For each meta-analysis, the expected number of significant studies was calculated by summing the statistical power estimates for each component study, under the assumption that the effect size of each study was equal to that of the largest study in the respective meta-analysis. This analysis of excess significance was performed using R software (version 4.5.1; R Foundation for Statistical Computing, Vienna, Austria).

### Quality assessment

The methodological quality of the included systematic reviews with meta-analyses was evaluated using the AMSTAR 2 questionnaire. AMSTAR 2 is consisted of 16 items that needs reviewers to answer “Yes” or “Partial Yes” or “No” or “Not a Meta-Analysis”. Two researchers (X-XC and Z-SJ) independently applied the tool to each eligible meta-analysis. The assessment focused on key methodological aspects such as the adequacy of the literature search, study selection and data extraction procedures, appropriateness of meta-analytical methods, consideration of risk of bias when interpreting results, and discussion of review limitations. Based on the assessment of these items, particularly the critical domains, an overall rating of confidence in the review findings was assigned (high, moderate, low, or critically low). Any disagreement was resolved through the consensus with the third reviewer.

### Evaluation of evidence credibility

We used credibility assessment criteria (**Supplementary Table 12**) to evaluate the strength of the evidence. Evidence from meta-analyses of observational studies with nominally significant summary results (*P* < 0.05) was classified into 4 categories: convincing, highly suggestive, suggestive, or weak evidence (class I, II, III, and IV, respectively). For meta-analyses performed on the same outcome, we examined the consistency between studies and the largest meta-analysis was retained for further analyses.

## Results

**Figure 1:**
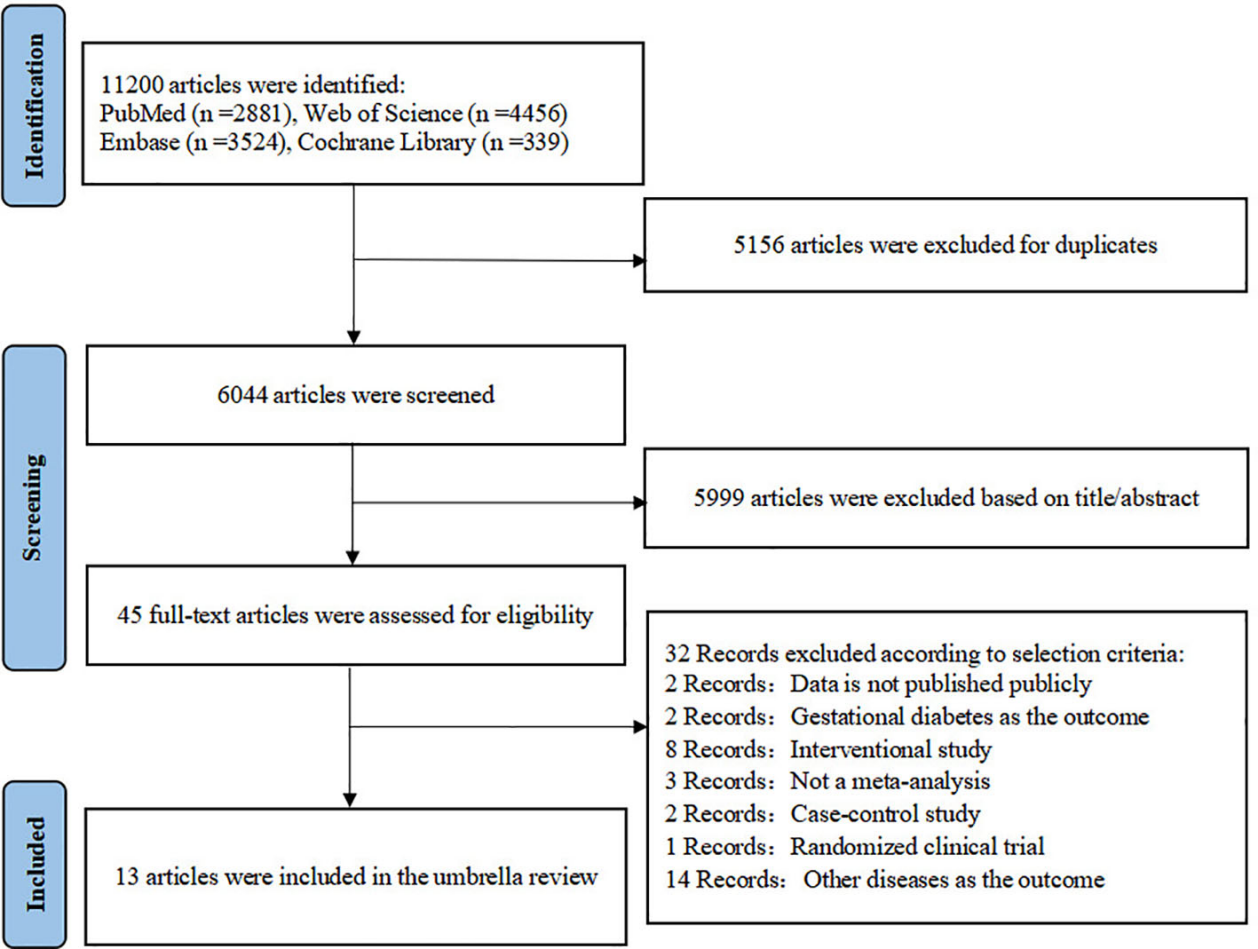
Study selection process.

A total of 11, 200 records were identified through the initial database search. After excluding 5, 156 duplicates, 6, 044 records were further investigated by screening the title and abstract. Subsequently, 45 full-text articles were assessed for eligibility. Following this assessment, several articles were excluded, with detailed reasons documented in **Supplementary Table 13**. Ultimately, 13 articles (comprising 13 meta-analyses) for 9 types of lipid biomarkers were included in this review. The literature search and screening process are summarized in **Figure 1**. One meta-analysis was identified for exploring the relationship of non-HDL (5), Lp(a) (7), and LAP (13) with the risk of T2D respectively, and two meta-analyses for TG/HDL (14, 15), VAI (16, 17), TyG (18, 19), HTW (20, 21), and FAs, respectively (22, 23).

The methodological quality of the 13 included systematic reviews was assessed using the AMSTAR 2 tool, a critical appraisal instrument for systematic reviews of healthcare interventions. The assessment was conducted in duplicate by two independent reviewers, with disagreements resolved through consensus. According to the AMSTAR 2 rating criteria, which emphasize performance on critical items, the overall confidence in the findings was rated as “Critically Low” for twelve reviews and “Low” for one; no review was rated as “Moderate” or “High.”.

Common methodological strengths observed across most reviews included comprehensive literature searches, clear reporting of PICO-based inclusion criteria, and appropriate methods for risk-of-bias assessment and meta-analysis. However, a prevalent limitation—particularly regarding the critical item on funding source reporting (Item 7)—was failed to describe the funding sources of the included primary studies. No critical flaws were identified in the formulation of research questions or review protocols. Detailed results of the quality assessment are presented in **Table 1**.

**Table 1:** The quality assessment of the included studies.

Study (first author, year)	Indicators of lipid metabolism	Disease	Q1	Q2↑	Q3	Q4↑	Q5	Q6	Q7↑	Q8	Q9↑	Q10	Q11↑	Q12	Q13↑	Q14	Q15↑	Q16	Quality grade	Total/CD
Mengqi, 2025	Non-high-density lipoprotein cholesterol	T2D	Y1	Y1	Y1	PY	Y1	Y1	N0	PY	PY	Y1	Y1	N0	N0	Y1	Y1	Y1	CL	11.5/4
Ellie, 2017	Lipoprotein(a)	T2D	Y1	PY	Y1	PY	Y1	Y1	N0	PY	N0	Y1	Y1	N0	N0	Y1	N0	Y1	CL	9.5/2
Hongjing, 2025	Triglyceride to HDL cholesterol ratio	T2D	Y1	Y1	Y1	PY	Y1	Y1	N0	PY	PY	N0	Y1	N0	N0	Y1	Y1	Y1	CL	10.5/4
Cheng, 2019	Triglyceride to HDL cholesterol ratio	T2D	Y1	PY	Y1	PY	Y1	Y1	N0	PY	N0	Y1	Y1	N0	N0	Y1	Y1	Y1	CL	10.5/3
Ruixue, 2024	Visceral adiposity index	T2D	Y1	Y1	Y1	PY	Y1	Y1	N0	PY	Y1	Y1	Y1	N0	N0	Y1	Y1	Y1	CL	12/4.5
Fang, 2024	Visceral adiposity index	T2D	Y1	Y1	Y1	PY	Y1	Y1	N0	PY	Y1	Y1	Y1	N0	N0	Y1	Y1	Y1	CL	12/4.5
Shaghayegh Khanmohammadi, 2022	Lipid accumulation product	T2D	Y1	PY	Y1	PY	Y1	Y1	N0	PY	PY	N0	Y1	N0	N0	Y1	N0	Y1	CL	9/2.5
Raymond, 2021	Triglyceride-glucose index	T2D	Y1	PY	Y1	PY	Y1	Y1	N0	PY	PY	N0	Y1	N0	Y1	Y1	Y1	Y1	L	11/4.5
Alessandra, 2020	Triglyceride-glucose index	T2D	Y1	Y1	Y1	PY	Y1	Y1	N0	PY	PY	Y1	Y1	N0	N0	Y1	Y1	Y1	CL	11.5/4
Yongcheng Ren, 2016	Hypertriglyceridemic waist	T2D	Y1	PY	Y1	PY	Y1	Y1	N0	PY	PY	Y1	Y1	N0	N0	Y1	Y1	Y1	CL	11/3.5
Chun-Ming, 2019	Hypertriglyceridemic waist	T2D	Y1	PY	Y1	PY	Y1	Y1	N0	PY	PY	Y1	Y1	N0	N0	Y1	Y1	Y1	CL	11/3.5
Lihua, 2019	Fatty Acids	T2D	Y1	Y1	Y1	PY	Y1	Y1	N0	PY	Y1	Y1	Y1	N0	N0	Y1	Y1	Y1	CL	12/4.5
Gengdong, 2020	Fatty Acids	T2D	Y1	PY	Y1	PY	Y1	Y1	N0	PY	PY	Y1	Y1	N0	N0	Y1	Y1	Y1	CL	11/3.5

T2D, Type 2 diabetes; Y, Yes; PY, Partial Yes; N, No; L, Low; CL, Critically Low.

In addition, **Supplementary Figures S1**-**S9** show the process of selection of original articles in conducting the updated meta-analyses. Overall, 74, 104 publications were retrieved from the initial search. After a systematic literature screening and exclusion (reasons documented in **Supplementary Tables 14**-**S22**), 39 new original articles were identified, including three for Lp(a) (24–26), six for VAI (27–32), ten for LAP (30, 33–41), four for TyG (42–45), two for HTW (46, 47), eight for AIP (48–55), and six for FAs (56–61). When combined with 94 original articles derived from the previously included meta-analyses, a total of 133 original articles were included in the study.

### Non-high-density lipoprotein cholesterol

We identified 4 prospective cohort studies with a total of 132, 003 participants to analyse the association of non-HDL-C and the occurrence of T2D. Pooled analysis using a random-effects model revealed a significant positive association between non-HDL-C and the risk of T2D (RR = 1.27, 95% CI: 1.07, 1.52, *I^2^* = 81.9%; **Supplementary Figure S10**).

The results of the sensitivity analysis indicated that the total effect size remained stable and did not show any significant change after the sequential deletion of each study (**Supplementary Figure S11**). The sensitivity analysis indicated that exclusion of the study by *In-Ho* et al. (2022) (62) substantially reduced the heterogeneity (*I^2^* = 25.3%). No evidence of small-study publication bias was detected by Egger’ s (*P* = 0.293) or Begg’ s tests (*P* = 0.734). Owing to the limited number of included studies (n = 4), subgroup analyses, funnel plot assessments, and dose–response analyses were precluded.

### Lipoprotein(a)

We identified 7 prospective cohort studies with a total of 49, 451 participants to analyse the association of Lp(a) and the occurrence of T2D. Pooled random-effects meta-analysis demonstrated an inverse association between Lp(a) and the risk of T2D development (RR = 0.73, 95% CI: 0.56, 0.96, *I^2^* = 76.6%; **Supplementary Figure S12**).

The results of the sensitivity analysis indicated that the total effect size remained stable and did not show any significant change after the sequential deletion of each study (**Supplementary Figure S13**), and after excluding the study of *Ellie* et al.’s (2017) (7) the heterogeneity was substantially reduced (*I^2^* = 41.5%). In the middle-aged and elderly population, the association was not significant (RR = 0.82, 95% CI: 0.63, 1.06; n = 5). In contrast, a significantly stronger inverse correlation was observed in the adult cohort of all ages (RR = 0.35, 95% CI: 0.18, 0.68; n = 2; **Supplementary Figure S14a**). The analysis conducted based on follow-up duration stratification showed that a reduction in Lp(a) had a more significant predictive value for T2D incidence in cohorts with ≤ 10 years of follow-up (RR = 0.60, 95% CI: 0.44, 0.83; n = 5), whereas the association was not significant in the two cohorts with > 10 years of follow-up (RR = 0.93, 95% CI: 0.65, 1.33; n = 2; **Supplementary Figure S14b**). Subsequently, publication bias was assessed using Egger’s and Begg’s tests, and the results from both tests consistently indicated no significant publication bias, with no evidence of small-study effects (Egger’s test: *P* = 0.189) or overall bias (Begg’s test: *P* = 0.548). Funnel plots and dose-response analysis as there was not enough data available.

### Triglyceride to HDL cholesterol ratio

We identified 16 prospective cohort studies (12 publications) with a total of 134, 389 participants to analyse the triglyceride to HDL cholesterol (TG/HDL) ratio and T2D risk. Random-effects meta-analysis indicated a positive association between the TG/HDL ratio and T2D risk (RR = 1.51, 95% CI: 1.36, 1.69, *I^2^* = 93.0%; **Supplementary Figure S15**).

The results of the sensitivity analysis indicated that the total effect size remained stable and did not show any significant change after the sequential deletion of each study (**Supplementary Figure S16**). Subgroup analysis by age revealed a significantly elevated risk of T2D in the middle-aged and elderly population (RR = 2.13, 95% CI: 1.85, 2.45; n = 9). A weaker yet still statistically significant association was also observed in the adult cohort of all ages (RR = 1.17, 95% CI: 1.09, 1.26; n = 7; **Supplementary Figure S17a**). When stratified by follow-up duration, a significant positive association was observed in studies with ≤ 10 years of follow-up (RR = 1.54, 95% CI: 1.35, 1.74; n = 11). The association remained significant in studies with > 10 years of follow-up (RR = 1.43, 95% CI: 1.19, 1.72; n = 5; **Supplementary Figure S17b**), although the effect size was somewhat attenuated compared to the shorter follow-up group. Egger’s test indicated significant small-study effects (*P* < 0.001), whereas Begg’s test did not reach statistical significance (*P* = 0.444). meanwhile, the funnel plot (**Supplementary Figure S18A**) also demonstrated evident asymmetry attributable to publication bias.

**Figure 2:**
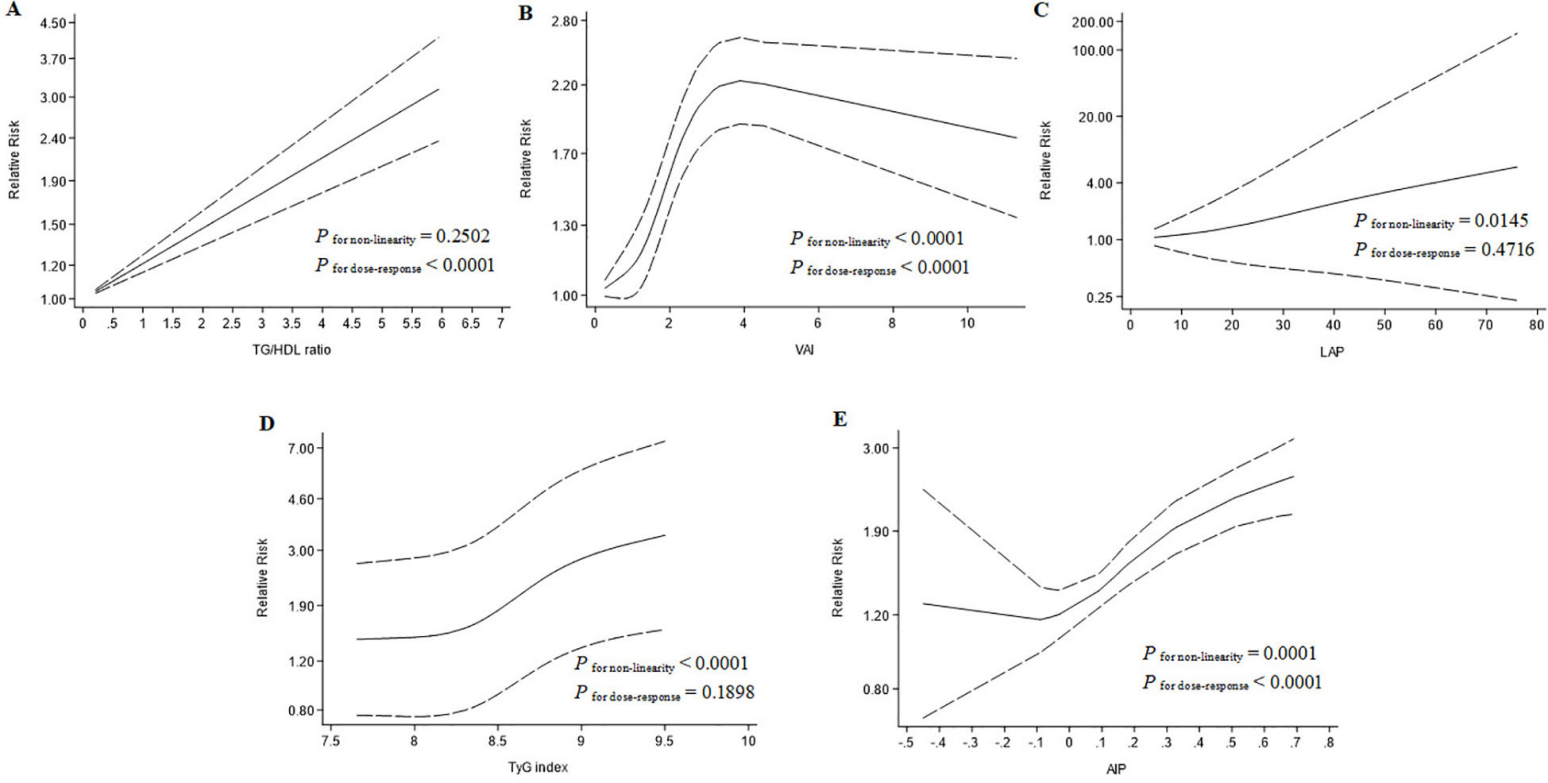
Dose‑response relationship between lipid metabolism indicators and relative risk of Type 2 Diabetes. **(A)** Triglyceride to high‑density lipoprotein cholesterol ratio; **(B)** Visceral adiposity index; **(C)** Lipid accumulation product; **(D)** Triglyceride glucose index; **(E)** Atherogenic index of plasma.

Among the cohort reports, 13 studies provided sufficient information and were used to conduct the linear dose-response meta-analysis. The analysis demonstrated a linear association between the TG/HDL-C ratio and T2D (*P*_for non-linearity_ = 0.2502; *P*_for dose-response_ < 0.0001; **Figure 2A**), with no evidence of departure from linearity at any threshold.

### Visceral adiposity index

We identified 28 prospective cohort studies (22 publications) with a total of 293, 297 participants to analyze the association between VAI and T2D risk. Using a random-effects model, meta-analysis revealed a positive association between the VAI and T2D risk (RR = 1.77, 95%CI: 1.61, 1.94, *I^2^* = 97.2%; **Supplementary Figure S19**).

The results of the sensitivity analysis indicated that the total effect size remained stable and did not show any significant change after the sequential deletion of each study (**Supplementary Figure S20**). Subgroup analyses revealed a significantly elevated risk in the adult cohort of all ages (RR = 2.08, 95% CI: 1.62, 2.66; n = 11). A statistically significant association was also observed in the middle-aged and elderly populations, though with a relatively lower risk estimate (RR = 1.71, 95% CI: 1.54, 1.89; n = 17; **Supplementary Figure S21a**). Based on follow-up duration stratification, a significant positive association was observed in studies with ≤ 10 years of follow-up (RR = 1.80, 95% CI: 1.62, 2.00; n = 24). The association remained significant in studies with > 10 years of follow-up (RR = 1.45, 95% CI: 1.37, 1.53; n = 4; **Supplementary Figure S21b**), though the effect size was somewhat attenuated compared to the shorter follow-up group. Multiple methods were conducted to assess the publication bias, and the results indicated the presence of small-study effects. Besides, Egger’s test indicated there is significant small-study effects (*P* = 0.030), corroborated by evident funnel plot asymmetry (**Supplementary Figure S18B**). In contrast, Begg’s test showed no significant bias (*P* = 0.199).

Among the cohort reports, 9 studies were used to conduct the non-linear dose-response meta-analysis and the analysis demonstrated there is a non-linear association between the VAI and T2D (*P*_for non-linearity_ < 0.0001; *P*_for dose-response_ < 0.0001; **Figure 2B**).

Meanwhile, we identified 7 prospective cohort studies with a total of 85, 892 participants to analyze the association of chinese visceral adiposity index (CVAI) and T2D risk. Using a random-effects model, meta-analysis revealed a positive association between the CVAI and T2D risk (RR = 2.48, 95% CI: 1.59, 3.88; *I^2^* = 95.8%; **Supplementary Figure S22**). The results of the sensitivity analysis indicated that the total effect size remained stable and did not show any significant change after the sequential deletion of each study (**Supplementary Figure S23**). No significant publication bias was detected, as both Egger’s (*P* = 0.944) and Begg’s tests (*P* = 0.548) showed no small-study or overall bias, but subgroup analyses and funnel plot analysis was omitted due to the limited number of studies (n = 7). Dose-response relationships could not be evaluated due to insufficient data.

### Lipid accumulation product

We identified 22 prospective cohort studies (16 publications) with a total of 169, 060 participants to analyze the association between the LAP and T2D risk. Meta-analysis using a random-effects model revealed a positive association between the LAP and T2D risk (RR = 2.94, 95% CI: 2.31, 3.73, *I^2^* = 93.4%; **Supplementary Figure S24**).

The results of the sensitivity analysis indicated that the total effect size remained stable and did not show any significant change after the sequential deletion of each study (**Supplementary Figure S25**). Subgroup analysis by age revealed a significantly elevated risk of T2D in the middle-aged and elderly population (RR = 3.20, 95% CI: 2.27, 4.51; n = 13). A weaker yet still statistically significant association was also observed in the adult cohort of all ages (RR = 2.58, 95% CI: 1.85, 3.60; n = 9; **Supplementary Figure S26**). Egger’s test indicated significant small-study effects (*P* = 0.005), whereas Begg’s test showed borderline significance (*P* = 0.048). meanwhile, the funnel plot (**Supplementary Figure S18C**) also demonstrated evident asymmetry attributable to publication bias.

Among the cohort reports, 7 studies provided information and were used to conduct the non-linear dose-response meta-analysis. The analysis demonstrated a non-linear association between the LAP and T2D (*P*_for non-linearity_ = 0.0145; *P*_for dose-response_ = 0.4716; **Figure 2C**).

### Triglyceride-glucose index

We identified 20 prospective cohort studies (17 publications) with a total of 84, 709 participants to analyze the association between the TyG index and T2D risk. Random-effects meta-analysis demonstrated a strong positive association between the TyG index and T2D risk (RR = 2.51, 95% CI: 2.13, 2.95; *I^2^* = 87.6%; **Supplementary Figure S27**).

The results of the sensitivity analysis indicated that the total effect size remained stable and did not show any significant change after the sequential deletion of each study (**Supplementary Figure S28**). Subgroup analyses revealed a significantly elevated risk in the adult cohort of all ages (RR = 3.30, 95% CI: 2.05, 5.32; n = 8). A statistically significant association was also observed in the middle-aged and elderly populations, though with a comparatively lower risk estimate (RR = 2.26, 95% CI: 1.90, 2.68; n = 12; **Supplementary Figure S29a**). When stratified by follow-up duration, a significant positive association was observed in studies with ≤ 10 years of follow-up (RR = 2.69, 95% CI: 2.24, 3.23; n = 17). The association remained significant in the subgroup with > 10 years of follow-up (RR = 1.62, 95% CI: 1.25, 2.11; n = 3; **Supplementary Figure S29b**), though with a relatively lower risk estimate. Publication bias was evident with a highly significant small-study effect in Egger’s test (*P* < 0.001) and borderline significance in Begg’s tests (*P* = 0.098). Furthermore, funnel plot asymmetry (**Supplementary Figure S18D**) also confirmed this bias.

Among the cohort reports, 15 studies provided sufficient information and were used to conduct the non-linear dose-response meta-analysis. The analysis demonstrated a non-linear association between the TyG index and T2D (*P*_for non-linearity_ < 0.0001; *P*_for dose-response_ = 0.1898; **Figure 2D**).

### Hypertriglyceridemic waist

We identified 7 prospective cohort studies (6 publications) with a total of 40, 660 participants to analyze the association between HTW phenotype and T2D risk. Meta-analysis using a random-effects model revealed a strong positive association between the HTW phenotype and T2D risk (RR = 3.54, 95% CI: 1.92, 6.53, *I^2^* = 94.2%; **Supplementary Figure S30**).

The results of the sensitivity analysis indicated that the total effect size remained stable and did not show any significant change after the sequential deletion of each study (**Supplementary Figure S31**). Sensitivity analysis excluding *Mohsen* et al. (2016) (63) and *Ge* et al. (2021) (47) substantially reduced heterogeneity (*I^2^* = 0.0%), yielding a stronger and more precise association (RR = 6.12, 95% CI: 5.26, 7.12). Subgroup analyses revealed a significantly elevated risk in the adult cohort of all ages (RR = 5.64, 95% CI: 4.11, 7.73; n = 4). In contrast, the association was not statistically significant in the middle-aged and elderly populations (RR = 2.37, 95% CI: 0.80, 6.99; n = 3; **Supplementary Figure S32**). No significant publication bias or small-study effects were observed (Egger’s *P* = 0.700; Begg’s *P* = 0.764). A funnel plot was not constructed due to the limited number of studies (n = 7) Dose-response relationships could not be evaluated due to insufficient data.

### Atherogenic index of plasma

We identified 10 prospective cohort studies (8 publications) with a total of 57, 907 participants to analyze the association between the AIP and T2D risk. Meta-analysis using a random-effects model revealed a positive association between the AIP and T2D risk (RR = 1.97, 95% CI: 1.54, 2.52, *I^2^* = 89.1%; **Supplementary Figure S33**).

The results of the sensitivity analysis indicated that the total effect size remained stable and did not show any significant change after the sequential deletion of each study (**Supplementary Figure S34**). Subgroup analyses showed statistically significant associations in both the middle-aged and elderly populations (RR = 2.17, 95% CI: 1.52, 3.08; n = 5) and the adult cohort of all ages (RR = 1.79, 95% CI: 1.20, 2.68; n = 5; **Supplementary Figure S35**), with a relatively higher point estimate observed in the older age group. Multiple methods were used to assess publication bias for the 10 included studies, visual inspection of the funnel plot (**Supplementary Figure S18E**) did not show obvious asymmetry, Egger’s test did not indicate significant small-study effects (*P* = 0.235), and Begg’s test did not suggest significant overall bias (*P* = 0.283).

Among the cohort reports, 4 studies provided information and were used to conduct the non-linear dose-response meta-analysis. The analysis demonstrated a non-linear association between the AIP and T2D (*P*_for non-linearity_ = 0.0001; *P*_for dose-response_ < 0.0001; **Figure 2E**).

### Fatty acids

We analyzed a series of prospective cohort studies (36 publications) with a total of 151, 048 participants using random-effects models, revealing differential associations between specific FAs subtypes and T2D risk.

Overall, SFAs exhibited divergent associations with T2D risk depending on chain length and structure (**Supplementary Figures S36–38**). Even-chain SFAs were associated with increased risk, with palmitic acid (C16:0) showing the strongest effect (RR = 1.19, 95% CI: 1.07, 1.33), followed by myristic acid (C14:0; RR = 1.10, 95% CI: 1.04, 1.17). In contrast, both odd-chain SFAs (C15:0: RR = 0.82, 95% CI: 0.73, 0.92; C17:0: RR = 0.71, 95% CI: 0.62, 0.81) and very long-chain SFAs (C20:0: RR = 0.78, 95% CI: 0.68, 0.90; C22:0: RR = 0.77, 95% CI: 0.66, 0.90) demonstrated inverse associations.

Monounsaturated Fatty Acids (MUFAs) showed heterogeneous associations with T2D risk (**Supplementary Figure S39**). Palmitoleic acid (C16:1n-7) had the strongest positive association (RR = 1.33, 95% CI: 1.19, 1.49). Other MUFAs varied, vaccenic acid (C18:1n-7) showed an inverse association (RR = 0.81, 95% CI: 0.68, 0.98), while isomers including gondoic acid (C20:1n-9; RR = 0.93, 95% CI: 0.80, 1.09) and nervonic acid (C24:1n-9; RR = 1.00, 95% CI: 0.95, 1.05) showed no significant association.

From an overall perspective, N-3 PUFAs were inversely associated with T2D risk (RR = 0.94, 95% CI: 0.91, 0.97; **Supplementary Figure S40**). Docosahexaenoic acid (DHA, C22:6n-3; RR = 0.91, 95% CI: 0.84, 0.98) and docosapentaenoic acid (DPA, C22:5n-3; RR = 0.92, 95% CI: 0.86, 0.98) showed the strongest inverse associations, while ALA and EPA showed non-significant associations.

Different classes of N-6 PUFAs pointed to different outcomes (**Supplementary Figure S41**). Linoleic acid (LA; C18:2n-6) was inversely related to T2D risk (RR = 0.80, 95% CI: 0.74, 0.87). In contrast, dihomo-γ-linolenic acid (DGLA; C20:3n-6) showed the strongest positive association (RR = 1.36, 95% CI: 1.21, 1.53), followed by γ-linolenic acid (GLA; C18:3n-6; RR = 1.24, 95% CI: 1.11, 1.39).

No significant association was observed for total trans FAs (RR = 0.86, 95% CI: 0.76, 0.97; **Supplementary Figure S42**). However, some individual trans FAs isomers showed opposing effects: trans-palmitoleic acid (C16:1n-7t) was inversely associated with risk (RR = 0.77, 95% CI: 0.62, 0.96), while its positional isomer C16:1n-9t was positively associated (RR = 1.40, 95% CI: 1.04, 1.89).

### Evidence assessment of included studies

**Figure 3:**
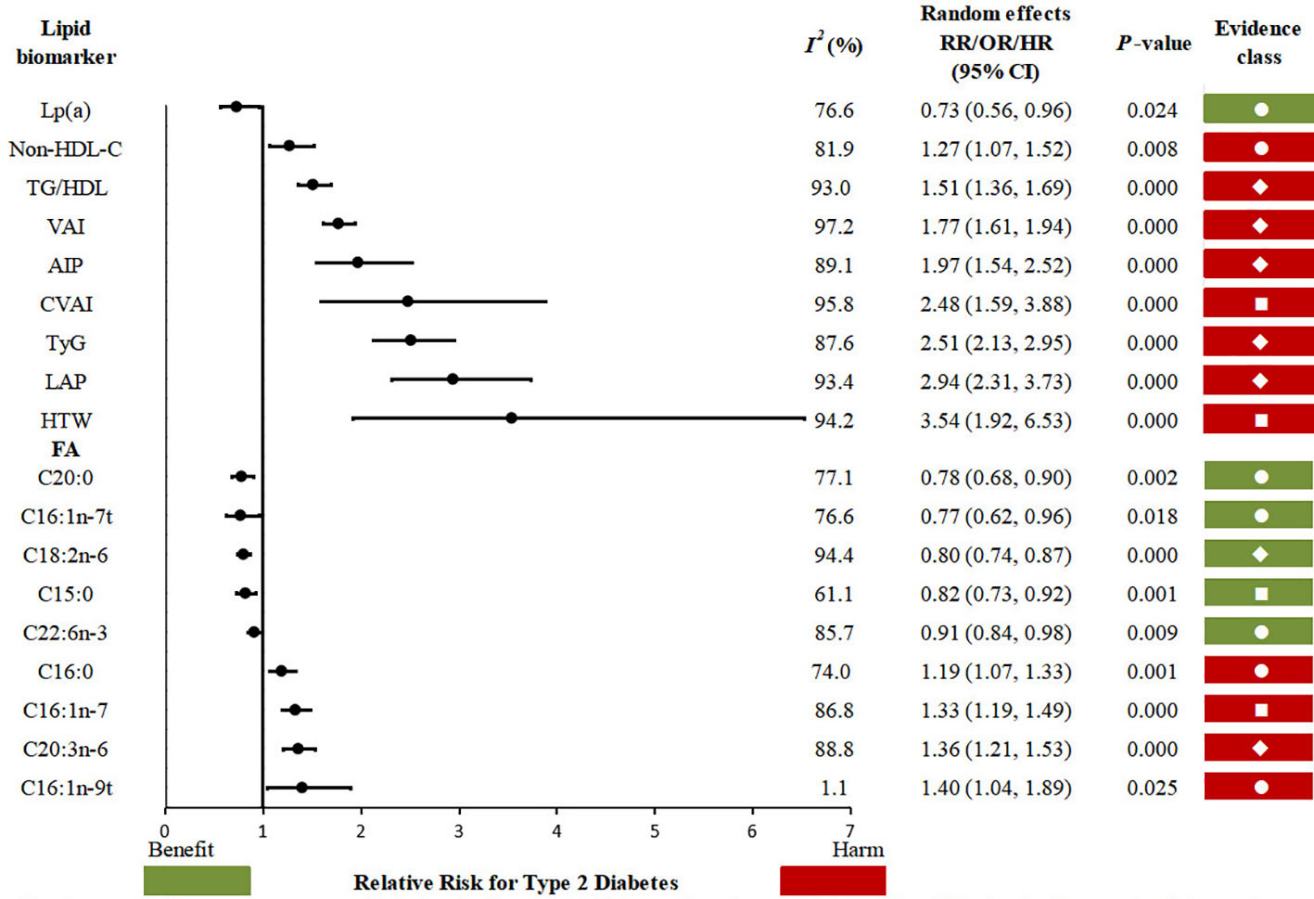
Forest plot of association between lipid metabolism indicators and type 2 diabetes.

Evidence assessment of the identified associations was performed according to our credibility assessment criteria. The summary of evidence classes and subgroup analysis is presented in **Table 2**. Seven meta-analyses showed a *P*-value below 10^−6^, seventeen had > 1000 cases (or > 20, 000 total participants for continuous outcomes), only one had no large heterogeneity (*I^2^* < 50%), and nine had neither small-study effects nor excess significance bias. After credibility assessment, no lipid metabolism indicator presented convincing evidence; seven lipid biomarkers presented highly suggestive evidence (class II: TG/HDL, VAI, LAP, TyG, AIP, LA, and DGLA); four presented suggestive evidence (class III: CVAI, HTW, C15:0, and C16:1n-7) and seven presented weak evidence (class IV: Lp(a), non-HDL, C16:0, C20:0, C16:1n-7t, C16:1n-9t, and DHA) for their associations with the occurrence of T2D (**Figure 3**).

**Table 2:** Summary of evidence classes and subgroup analysis for the associations between lipid metabolic indicators and Type 2 diabetes.

Lipid metabolic indicators	Study design	Studies,n	Case,n	Total participants,n	Random effects RR/OR/HR (95% CI)	*P* value *	I^2^(%)	Egger's *P*↑	Observed ↕	Expected ↕	*P* value for excess significance test	Evidence class
Non-high-density lipoprotein cholesterol	PCS	4	14,527	132,003	1.27 (1.07, 1.52)	0.008	81.9	0.293	3	3	0.4181	IV
Lipoprotein(a)												
All studies	PCS	7	2,604	49,451	0.73 (0.56, 0.96)	0.024	76.6	0.189	5	3	0.2567	IV
Age												
Adult cohort of all ages		2	76	869	0.35 (0.18, 0.68)	0.002	0.0					
Middle-aged and elderly population cohort		5	2,528	48,582	0.82 (0.63, 1.06)	0.127	78.2					
Length of follow-up												
≤ 10		5	840	21,890	0.60 (0.44, 0.83)	0.002	30.9					
> 10		2	1,764	27,561	0.93(0.65, 1.33)	0.703	90.0					
Triglyceride to HDL cholesterol ratio												
All studies	PCS/NCC	16	9,043	134,389	1.51 (1.36, 1.69)	0.000	93.0	0.000	15	12	0.0121	II
Age												
Adult cohort of all ages		7	5,334	86,058	1.17(1.09, 1.26)	0.000	87.8					
Middle-aged and elderly population cohort		9	3,709	48,331	2.13(1.85, 2.45)	0.000	32.0					
Length of follow-up												
≤ 10		11	6,960	117,737	1.54(1.35, 1.74)	0.000	82.1					
> 10		5	2,083	16,652	1.43(1.19, 1.72)	0.000	96.2					
Visceral adiposity index												
All studies	PCS	28	18,993	293,297	1.77 (1.61, 1.94)	0.000	97.2	0.030	26	23	0.0288	II
Age												
Adult cohort of all ages		11	2,462	54,267	2.08(1.62, 2.66)	0.000	92.9					
Middle-aged and elderly population cohort		17	16,532	239,030	1.71(1.54, 1.89)	0.000	97.1					
Length of follow-up												
≤ 10		24	12,231	116,031	1.80(1.62, 2.00)	0.000	94.2					
> 10		4	6,762	177,266	1.45(1.37, 1.53)	0.000	82.8					
Chinese visceral adiposity index	PCS	7	9,241	85,892	2.48 (1.59, 3.88)	0.000	95.8	0.944	7	7	0.4265	III
Lipid accumulation product												
All studies	PCS	22	13,483	169,060	2.94(2.31, 3.73)	0.000	93.4	0.005	21	21	0.3588	II
Age												
Adult cohort of all ages		9	6,168	90,219	2.58(1.85, 3.60)	0.000	92.3					
Middle-aged and elderly population cohort		13	7,315	78,841	3.20(2.27, 4.51)	0.000	93.4					
Triglyceride-glucose index												
All studies	PCS	20	9,621	84,709	2.51 (2.13, 2.95)	0.000	87.6	0.000	20	18	0.0741	II
Age												
Adult cohort of all ages		8	2,151	34,190	3.30(2.05, 5.32)	0.000	90.3					
Middle-aged and elderly population cohort		12	7,470	50,519	2.26(1.90, 2.68)	0.000	85.8					
Length of follow-up												
≤ 10		17	8,841	77,097	2.69(2.24, 3.23)	0.000	87.3					
> 10		3	780	7,612	1.62(1.25, 2.11)	0.000	48.0					
Hypertriglyceridemic waist												
All studies	PCS	7	2,781	40,660	3.54 (1.92, 6.53)	0.000	94.2	0.700	6	6	0.6228	III
Age												
Adult cohort of all ages		4	785	15,681	5.64(4.11, 7.73)	0.000	14.4					
Middle-aged and elderly population cohort		3	1,996	24,979	2.37(0.80, 6.99)	0.118	97.8					
Atherogenic index of plasma												
All studies	PCS	10	10,484	57,907	1.97 (1.54, 2.52)	0.000	89.1	0.235	8	9	0.8655	II
Age												
Adult cohort of all ages		5	6,902	25,314	1.79(1.20, 2.68)	0.005	88.1					
Middle-aged and elderly population cohort		5	3,582	32,593	2.17(1.52, 3.08)	0.000	91.1					
FAs												
All studies	PCS/NCC	43	38,436	151,048								
SFA-C16:0	PCS/NCC	19	17,727	58,822	1.19 (1.07, 1.33)	0.001	74.0	0.501	9	6	0.0349	IV
SFA-C15:0	PCS/NCC	10	15,796	40,703	0.82 (0.73, 0.92)	0.001	61.1	0.979	4	4	0.4974	III
SFA-C20:0	PCS/NCC	13	14,903	50,262	0.78 (0.68, 0.90)	0.002	77.1	0.134	9	6	0.0812	IV
MUFA-C16:1n-7	PCS/NCC	17	5,585	30,853	1.33 (1.19, 1.49)	0.000	86.8	0.000	10	11	0.6270	III
trans FA-C16:1n-7t	PCS/NCC	6	2,327	17,671	0.77 (0.62, 0.96)	0.018	76.6	0.061	2	3	0.8224	IV
trans FA-C16:1n-9t	PCS	3	486	3,302	1.40 (1.04, 1.89)	0.025	1.1	0.643	1	1	0.3783	IV
PUFA-C22:6n-3	PCS/NCC	18	20,163	75,201	0.91 (0.84, 0.98)	0.009	85.7	0.046	4	5	0.5462	IV
PUFA-C18:2n-6	PCS/NCC	22	20,265	74,177	0.80 (0.74, 0.87)	0.000	94.4	0.002	13	12	0.6433	II
PUFA-C20:3n-6	PCS/NCC	16	17,395	54,465	1.36 (1.21, 1.53)	0.000	88.8	0.002	10	11	0.4171	II

Observed, Number of studies with nominally significant results (*P*  < 0.05).

Expected, Expected number of significant studies, calculated from the summed statistical power of component studies.

Excess significance test *P* value: *P*  < 0.05 indicates potential excess significance bias.

PCS, Prospective cohort study; NCC, Nested case-control study; FA, Fatty acids; SFA, Saturated fatty acids; MUFA, Monounsaturated fatty acids; PUFA, Polyunsaturated fatty acids.

## Discussion

This study was conducted based on prospective cohort data, providing stronger evidence for causal inference compared to the cross-sectional study by establishing temporal order (exposure before outcome). Our dose-response analyses, though not always monotonic, reveal non-random relationships that support an association. Nonetheless, observational studies cannot prove causation; future Mendelian randomization or randomized trials are needed to confirm causality. By re-analyzing long-term follow-up data on the relationship between a series of lipid metabolism indicators and the risk of T2D, we obtained the HRs of the following indicators separately: Non-HDL-C (HR = 1.27), Lp(a) (HR = 0.73), TG/HDL-C ratio (HR = 1.51, *P*_for non-linearity_ = 0.2502), VAI (HR = 1.77), LAP (HR = 2.94), TyG index (HR = 2.51), HTW phenotype (HR = 3.54), and AIP (HR = 1.97). Simultaneously, the study also analyzed the relationship between different types of peripheral FAs levels and T2D risk. Specifically, the even-chain SFA palmitic acid (C16:0) was positively associated with T2D risk (RR = 1.19), whereas odd-chain SFAs (e.g., C15:0; RR = 0.82) and very long-chain SFAs (e.g., C20:0; RR = 0.78) showed inverse associations. For MUFAs, palmitoleic acid (C16:1n-7) demonstrated a strong positive correlation with risk (RR = 1.33), and among PUFAs, n-3 PUFAs like DHA (RR = 0.91) and the n-6 PUFA linoleic acid (LA; RR = 0.80) were inversely related to T2D risk, while another n-6 PUFA, dihomo-γ-linolenic acid (DGLA, C20:3n-6), was positively associated (RR = 1.36). Furthermore, individual trans FA isomers displayed opposing relationships: trans-palmitoleic acid (C16:1n-7t) was inversely associated with risk (RR = 0.77), while its positional isomer C16:1n-9t was positively associated (RR = 1.40).

Regarding traditional lipid metabolism indicators, this study found that elevated non-HDL-C was significantly associated with an increased risk of T2D (RR = 1.27), consistent with the results of a prospective study by *Han* et al. (2025) (5), further confirming the clinical value of non-HDL-C as a predictor of diabetes. Non-HDL-C encompasses all atherogenic lipoprotein particles except HDL, including LDL, IDL, VLDL, and their remnants. Its elevation reflects an increased overall burden of atherogenic lipoproteins in the circulation. Pathophysiologically, elevated non-HDL-C levels may promote the ectopic lipid deposition (e.g., in pancreatic, liver, and muscle tissues), induce endoplasmic reticulum stress and mitochondrial dysfunction, and activate inflammatory signaling pathways, thereby impairing insulin secretion and aggravating peripheral insulin resistance (1). With respect to Lp(a), this study found an inverse association with T2D risk, wherein higher levels were associated with a lower risk (RR = 0.73). This result aligns with the conclusion proposed by *Paige* et al. (2017) (7), who reported that high Lp(a) levels associated with lower risk of diabetes, but differs from some other studies. For instance, in a large female cohort (the Women’s Health Study), *Mora* et al. (2010) (64), reported no significant association or a slight positive correlation between Lp(a) and T2D risk. Several factors may account for this discrepancy. Firstly, differences in population characteristics likely played an important influencing factor: the participants of *Mora* et al. (2010) (64) were primarily middle-aged and older women (mean age > 60 years), whereas subgroup analyses in this present study suggest that the negative correlation of Lp(a) is more pronounced in adult general populations covering a broader age range. Secondly, variation in follow-up duration might also contribute to inconsistent results. The follow-up time in *Mora* et al. (2010) (64) exceeded 10 years; over such a extended timeframe, age-related metabolic changes, cardiovascular complications, and concomitant medications might have gradually obscured the initial impact of Lp(a) on glucose metabolism, confounding its true effect. Subgroup analyses in this study also indicated similar outcome where Lp(a) had lost its predictive value for T2D in cohorts with follow-up durations exceeding10 years. From a mechanistic perspective, Lp(a) is a complex lipoprotein composed of an LDL-like particle covalently linked via a disulfide bond to the specific Apo(a). Some studies suggest that high concentrations of Lp(a) may competitively inhibit the activity of Proprotein Convertase Subtilisin/Kexin type 9 (PCSK9), thereby indirectly enhancing the sensitivity of insulin signaling pathways. However, higher Lp(a) is not invariably beneficial, as substantial evidence clearly establishes high Lp(a) levels as an independent risk factor for Atherosclerotic Cardiovascular Disease (ASCVD) and Calcific Aortic Valve Stenosis (CAVS). Therefore, a comprehensive assessment of Lp(a)’s clinical significance requires balancing its potential metabolic benefits against its established cardiovascular risks.

Among the composite lipid related formula, weight related parameters and triglycerides are the main constituent factors.A large dose-response meta-analysis published by *Ahmad* et al. (2022) (65)(covering 216 cohort studies) confirmed not only a strong positive linear association between BMI and T2D risk (a 72% increase in risk per 5-unit increase) but also independent associations for central obesity indicators: WC, waist-to-hip ratio, and waist-to-height ratio, with each 10 cm, 0.1 unit, and 0.1 unit increase associated with 61%, 63%, and 73% increased risk, respectively. Composite indices that combine TG with body weight (e.g., LAP) or with fasting glucose (TyG index) could predict T2D risk more strongly than single lipid parameters. Among these, the TyG index is a parameter that has recently gained widespread attention: it uses low-cost, widely available fasting glucose and TG, and predicts risk even when fasting glucose is normal. Clinically, TyG could be used as a first-line indicator to conduct preliminary screening, and confirmatory glucose testing (such as OGTT) should be performed in patients with significantly elevated TyG value. In recent years, some new formulas derived from TyG, such as TyG-BMI, TyG-WC, TyG-WHtR, and TyG-BRI are constantly being developed, which may have better predictive value for the risk of diabetes, but also needs to be verified by large samples and long-term cohort research (66). To improve prediction accuracy, we suggest adopt a combination of classification evaluation and multidimensional evaluation: WC-based indices, such as HTW and LAP, may more suitable obese individuals, but in non-overweight populations, their predictive power may be greatly reduced; meanwhile, try to use a variety of T2D risk prediction formulas, which may significantly improve the prediction accuracy. At the same time, challenges cannot be ignored either.: how to obtain precise cut-off points for each indicator still needs to be explored through research on large sample populations.

FAs, as fundamental components of lipids, also possess potential predictive value for the occurrence of T2D. High levels of even-chain SFAs (e.g., C14:0, C16:0) are considered primary drivers of lipotoxicity and increased T2D risk, originating from diet (e.g., red meat, dairy fat) and hepatic *de novo* lipogenesis (a process strongly stimulated by high-carbohydrate diets). In contrast, odd-chain (e.g., C15:0, C17:0) and very long-chain SFAs (e.g., C20:0, C22:0) exhibit clear negative correlations with the occurrence of T2D. For instance, C15:0 and C17:0 cannot be synthesized *de novo* and metabolized via ω-oxidation, avoiding lipotoxic intermediates and potentially enhancing insulin sensitivity. Very long-chain SFAs are associated with cell membrane integrity and anti-inflammatory signaling. Regarding MUFAs, palmitoleic acid (C16:1n-7) is a marker of endogenous lipid synthesis;elevated levels reflect carbohydrate-induced hepatic *de novo* lipogenesis, and are strongly correlated with high TG, insulin resistance, and high T2D risk-consistent with the finding of *Gengdong* et al.’s (2020) (23). In contrast, trans-palmitoleic acid (C16:1n-7t) from dairy products is associated with a lower risk of diabetes and plays essential roles in improving insulin sensitivity and regulating adipose tissue function. In addition, N-3 PUFAs (represented by DHA) exert protective effects through potent anti-inflammatory and cell membrane repair actions. Among n-6 PUFAs, LA shows a negative correlation between serum levels and T2D risk, whereas its metabolites, such as DGLA, demonstrate a clear risk-increasing effect. The divergent effects of different metabolites within the same metabolic pathway underscore the complex role of n-6 PUFAs metabolic balance in the pathogenesis of diabetes. Overall, the impact of FAs on T2D risk demonstrates a clear structure-activity relationship with predictive value determined not only by category affiliation but also closely related to specific molecular structure, dietary source, and metabolic pathway.

In our study, potential sources of heterogeneity were explored through pre-specified subgroup and sensitivity analyses. The heterogeneity may mainly stem from the following aspects. From the clinical perspective, differences in the age of the study population and follow-up duration were key factors. For example, in the analysis of the TG/HDL-C ratio, TyG index, VAI, Lp(a), etc., the predictive performance of these biomarkers was consistently stronger in studies with shorter follow-up periods (≤ 10 years) compared to those with longer follow-up (> 10 years). This pattern can be explained by the cumulative effect of clinical confounding factors over extended follow-up periods. With prolonged observation periods, factors such as population aging, development of comorbidities (particularly cardiovascular and metabolic diseases), and initiation of pharmacotherapies (e.g., statins or antihypertensive agents) may attenuate or obscure the initial association between biomarkers and T2D incidence. Therefore, while both age structure and follow-up duration contributed to heterogeneity, the diminished predictive performance over longer periods suggests these lipid metabolism indicators are particularly valuable for short to medium-term T2D risk prediction. Their long-term predictive value, however, requires careful interpretation taking into account the potential confounding effects of evolving clinical conditions over time. In addition, differences in baseline glucose metabolism status (e.g., normoglycemia vs. prediabetes) can modify the predictive value of lipid biomarkers. From the clinical research design perspective, differences in the effect measures reported by the included studies-such as HR, OR, and RR-may also be an important source of heterogeneity, even though these measures can be transformed under certain conditions. In addition, differences in the standardization of data reporting between different studies also contributed to the inconsistency in which some studies only reported effect estimates based on quartile groups, while others provided risk ratios per unit increment. Although a uniform increment model was applied for conversion, this discrepancy in data sources undoubtedly increased heterogeneity. Furthermore, variability in biomarker measurement methods (e.g., enzymatic assays vs. nuclear magnetic resonance spectroscopy) and lipid particle subfractions may contribute to heterogeneity. Adjustment for confounders also varied considerably across primary studies; for example, some studies controlled for body mass index while others did not. Finally, differences in laboratory measurement methods, instruments for blood lipid indicators, and adjustment strategies for confounding factors across the original studies likely also contributed to heterogeneity. Future primary research should standardize measurement protocols and confounder adjustment to reduce heterogeneity.

This study further conducted a comprehensive assessment of publication bias for the included lipid metabolism indicators. Multiple methods-including Egger’s regression test, Begg’s rank correlation test, and visual inspection of funnel plot asymmetry-were used for the comprehensive evaluation. For several composite indicators such as the TG/HDL-C ratio, VAI, LAP, and TyG index, multiple tests consistently indicated significant publication bias. Such bias could lead to an overestimation of the true effect size of the association between these indicators and T2D risk. Although sensitivity analyses indicated that the direction and significance of these associations remained stable after removing individual borderline studies, the presence of publication bias suggests that their effect sizes should be interpreted with cautious optimism. In contrast, for traditional lipid metabolism Indicators such as Lp(a) and non-HDL-C indicated no significant publication bias detected in the current analysis. This may be explained by the relative maturity of research on these indicators, the larger sample sizes of the included studies, or relatively better acceptance and publication status of negative results in this field, collectively make their pooled results potentially more robust and closer to the true association.

The methodological quality of the included meta-analyses was evaluated using the AMSTAR 2 tool and the strength of evidence for each association was systematically rated based on pre-defined credibility criteria. Evidence grading comprehensively considered multiple dimensions such as statistical significance, study sample size, heterogeneity, and publication bias. The evaluation results showed that among the associations between the investigated lipid metabolism indicators and T2D risk, no indicator was found to possess “Convincing” (Class I) evidence. Seven composite indicators-the TG/HDL-C ratio, VAI, LAP, TyG index, AIP, LA, and DGLA- were classified as “Highly Suggestive” (Class II), demonstrating strong statistical significance, biological plausibility and consistency across studies with sufficient sample sizes, despite the presence of moderate heterogeneity. In comparison, the CVAI, HTW phenotype, C15:0, and C16:1n-7 received “Suggestive” (Class III) evidence support. The associations of Lp(a), non-HDL-C, C16:0, C20:0, C16:1n-7t, C16:1n-9t, and DHA with T2D risk only received “Weak” (Class IV) evidence grades, primarily due to limited statistical significance in the relevant meta-analyses and a limited number of original studies, leading to high uncertainty in the conclusions.

This study also has several limitations. Firstly, the number of available prospective cohort studies for certain lipid indicators [such as non-HDL-C, Lp(a)] remains limited, which restricts the statistical power of the meta-analysis and precludes reliable dose-response relationship analyses or in-depth subgroup analyses for all associations. Secondly, significant heterogeneity persisted in some studies even after subgroup and sensitivity analyses, affecting the precision of the pooled effect estimates and the evidence grading. Thirdly, the search was restricted to English-language publications, which might unavoidably cause the omission of some important researches.

Future research can be developed from the following aspects. Firstly, given that most included studies were rated as low or critically low confidence by AMSTAR-2, the foremost priority is to promote large-scale, multi-center, standardized prospective cohort studies – particularly for indicators with currently weak evidence [e.g., Lp(a)] – to move beyond hypothesis-generating findings toward definitive conclusions. Moreover, expanding sample sizes is needed to verify generalizability across different populations. Methodologically, future studies are encouraged to adopt standardized reporting practices by uniformly expressing association strength using HR or RR per unit increment and providing detailed data for continuous exposure whenever possible. This would facilitate accurate dose-response analysis and reduce heterogeneity during meta-analysis. Secondly, to address current issues in data collection and metabolomics detection/reporting, there is an urgent need to strengthen standardized data management. Inconsistent protocols across laboratories and incomplete reporting of metabolomic measurements remain major barriers to reproducibility and cross-study comparability. Future research should establish and adhere to unified guidelines for sample handling, quality control, and data documentation. Thirdly, leveraging big data and machine learning approaches that integrate multiple domains – including phenotypes (body weight, WC), routine clinical indicators, and metabolomic profiles – holds great promise for developing more accurate risk prediction models. Such integrative models can capture complex, non-linear interactions and enable personalized prevention strategies for T2D.

This study confirmed the strong predictive ability of various lipid and glucose metabolism-derived indicators (e.g., LAP). Integrating traditional lipid metabolism Indicators (e.g., TG, HDL-C), composite indicators (e.g., LAP, TyG, VAI), glucose markers (e.g., fasting glucose, HbA1c), genetic/inflammatory factors, and clinical characteristics (e.g., age, BMI, family history) - combine with machine learning - could enable more accurate, personalized T2D risk prediction. Such models would facilitate early identification of high-risk individuals and targeted interventions, contributing to the primary prevention of T2D.

## Conclusion

This review systematically evaluated the evidence for associations between lipid metabolism biomarkers and T2D risk, yielding the following conclusions. Composite lipid metabolism indicators, the HTW, LAP, TyG index, AIP, VAI and TG/HDL-C ratio, showed potential suggestive value in the prediction of T2D and were rated as “Highly Suggestive” evidence grade. Among single lipid metabolism indicators, non-HDL-C was positively correlated with T2D risk, while Lp(a) showed a negative correlation, but both were rated as “Weak” evidence strength due to high heterogeneity or a limited number of studies. The analysis of FAs reflected their complex structure-activity relationship: even-chain SFAs were associated with increased risk, while odd-chain and very long-chain SFAs and some n-3 PUFAs were associated with protective effects. Most of these associations were graded as “Suggestive” to “Weak” evidence. In summary, this study systematically updated and integrated currently available data from prospective research, providing supportive evidence for considering lipid metabolism biomarkers in early T2D risk assessment and in the development of personalized prevention and intervention strategies, but it should be noted that all included meta-analyses were graded as class II to IV evidence and rated as low or critically low confidence using AMSTAR-2, these findings should be interpreted with caution.

## References

[1] Tushuizen ME Bunck MC Pouwels PJ Bontemps S van Waesberghe JH Schindhelm RK . Pancreatic fat content and beta-cell function in men with and without type 2 diabetes. Diabetes Care. (2007) 30:2916–21. doi: 10.2337/dc08-0184 17666465

[2] Savage DB Petersen KF Shulman GI . Disordered lipid metabolism and the pathogenesis of insulin resistance. Physiol Rev. (2007) 87:507–20. doi: 10.1152/physrev.00024.2006. PMID: 17429039 PMC2995548

[3] Rütti S Ehses JA Sibler RA Prazak R Rohrer L Georgopoulos S . Low- and high-density lipoproteins modulate function, apoptosis, and proliferation of primary human and murine pancreatic beta-cells. Endocrinology. (2009) 150:4521–30. doi: 10.1210/en.2009-0252 19628574

[4] Grundy SM Stone NJ Bailey AL Beam C Birtcher KK Blumenthal RS . 2018 AHA/ACC/AACVPR/AAPA/ABC/ACPM/ADA/AGS/APhA/ASPC/NLA/PCNA Guideline on the management of blood cholesterol: A report of the American College of Cardiology/American Heart Association Task Force on Clinical Practice Guidelines. Circulation. (2019) 139:e1082–143. doi: 10.1161/cir.0000000000000625. PMID: 30586774 PMC7403606

[5] Han M Shen Y Guo X Hong C Ji X Guo H . Association between non-high-density lipoprotein cholesterol and type 2 diabetes: a systematic review and meta-analysis of cohort studies. Endocr J. (2025) 72:43–51. doi: 10.1507/endocrj.ej24-0189. PMID: 39313371 PMC11778345

[6] Tsimikas S . A test in context: Lipoprotein(a): Diagnosis, prognosis, controversies, and emerging therapies. J Am Coll Cardiol. (2017) 69:692–711. doi: 10.1016/j.jacc.2016.11.042 28183512

[7] Paige E Masconi KL Tsimikas S Kronenberg F Santer P Weger S . Lipoprotein(a) and incident type-2 diabetes: results from the prospective Bruneck study and a meta-analysis of published literature. Cardiovasc Diabetol. (2017) 16:38. doi: 10.1186/s12933-017-0520-z. PMID: 28320383 PMC5359972

[8] Wu X Yu Z Su W Isquith DA Neradilek MB Lu N . Low levels of ApoA1 improve risk prediction of type 2 diabetes mellitus. J Clin Lipidol. (2017) 11:362–8. doi: 10.1016/j.jacl.2017.01.009. PMID: 28502492

[9] Ley SH Harris SB Connelly PW Mamakeesick M Gittelsohn J Wolever TM . Association of apolipoprotein B with incident type 2 diabetes in an aboriginal Canadian population. Clin Chem. (2010) 56:666–70. doi: 10.1373/clinchem.2009.136994. PMID: 20110448 PMC5123873

[10] Mackey RH Mora S Bertoni AG Wassel CL Carnethon MR Sibley CT . Lipoprotein particles and incident type 2 diabetes in the multi-ethnic study of atherosclerosis. Diabetes Care. (2015) 38:628–36. doi: 10.2337/dc14-0645. PMID: 25592196 PMC4370328

[11] Amato MC Giordano C Galia M Criscimanna A Vitabile S Midiri M . Visceral adiposity index: a reliable indicator of visceral fat function associated with cardiometabolic risk. Diabetes Care. (2010) 33:920–2. doi: 10.2337/dc09-1825. PMID: 20067971 PMC2845052

[12] Page MJ McKenzie JE Bossuyt PM Boutron I Hoffmann TC Mulrow CD . The PRISMA 2020 statement: an updated guideline for reporting systematic reviews. Bmj. (2021) 372:n71. doi: 10.31222/osf.io/v7gm2. PMID: 33782057 PMC8005924

[13] Khanmohammadi S Tavolinejad H Aminorroaya A Rezaie Y Ashraf H Vasheghani-Farahani A . Association of lipid accumulation product with type 2 diabetes mellitus, hypertension, and mortality: a systematic review and meta-analysis. J Diabetes Metab Disord. (2022) 21:1943–73. doi: 10.1007/s40200-022-01114-z. PMID: 36404835 PMC9672205

[14] Zhong H Luo L Wang X Xiao Y . Association between triglyceride to HDL cholesterol ratio and a risk of diabetes mellitus: a systematic review and meta-analysis. Lab Med. (2025) 56:1–6. doi: 10.1093/labmed/lmae052. PMID: 39066659

[15] Cheng C Liu Y Sun X Yin Z Li H Zhang M . Dose-response association between the triglycerides: High-density lipoprotein cholesterol ratio and type 2 diabetes mellitus risk: The rural Chinese cohort study and meta-analysis. J Diabetes. (2019) 11:183–92. doi: 10.1111/1753-0407.12836. PMID: 30091266

[16] Deng R Chen W Zhang Z Zhang J Wang Y Sun B . Association between visceral obesity index and diabetes: a systematic review and meta-analysis. J Clin Endocrinol Metab. (2024) 109:2692–707. doi: 10.1210/clinem/dgae303. PMID: 38709677 PMC11403314

[17] Shen F Guo C Zhang D Liu Y Zhang P . Visceral adiposity index as a predictor of type 2 diabetes mellitus risk: a systematic review and dose-response meta-analysis. Nutr Metab Cardiovasc Dis. (2024) 34:811–22. doi: 10.1016/j.numecd.2023.04.009. PMID: 38326187

[18] Pranata R Huang I Irvan Lim MA Vania R . The association between triglyceride-glucose index and the incidence of type 2 diabetes mellitus-a systematic review and dose-response meta-analysis of cohort studies. Endocrine. (2021) 74:254–62. doi: 10.1007/s12020-021-02780-4. PMID: 34086260

[19] da Silva A Caldas APS Rocha D Bressan J . Triglyceride-glucose index predicts independently type 2 diabetes mellitus risk: a systematic review and meta-analysis of cohort studies. Prim Care Diabetes. (2020) 14:584–93. doi: 10.1016/j.pcd.2020.09.001. PMID: 32928692

[20] Ren Y Luo X Wang C Yin L Pang C Feng T . Prevalence of hypertriglyceridemic waist and association with risk of type 2 diabetes mellitus: a meta-analysis. Diabetes Metab Res Rev. (2016) 32:405–12. doi: 10.1002/dmrr.2725. PMID: 26417844

[21] Ma CM Liu XL Lu N Wang R Lu Q Yin FZ . Hypertriglyceridemic waist phenotype and abnormal glucose metabolism: a system review and meta-analysis. Endocrine. (2019) 64:469–85. doi: 10.1007/s12020-019-01945-6. PMID: 31065910

[22] Huang L Lin JS Aris IM Yang G Chen WQ Li LJ . Circulating saturated fatty acids and incident type 2 diabetes: a systematic review and meta-analysis. Nutrients. (2019) 11. doi: 10.3390/nu11050998. PMID: 31052447 PMC6566227

[23] Chen G Li Y Zeng F Deng G Liang J Wang J . Biomarkers of fatty acids and risk of type 2 diabetes: a systematic review and meta-analysis of prospective cohort studies. Crit Rev Food Sci Nutr. (2021) 61:2705–18. doi: 10.1080/10408398.2020.1784839. PMID: 32598176

[24] Kaya A Onat A Yüksel H Can G Yüksel M Ademoğlu E . Lipoprotein(a)-activated immunity, insulin resistance and new-onset diabetes. Postgrad Med. (2017) 129:611–8. doi: 10.1080/00325481.2017.1342508. PMID: 28633585

[25] Muhanhali D Zhai T Cai Z Ling Y . Lipoprotein(a) concentration is associated with risk of type 2 diabetes and cardiovascular events in a Chinese population with very high cardiovascular risk. Endocrine. (2020) 69:63–72. doi: 10.1007/s12020-020-02286-5. PMID: 32253681

[26] Skoumas I Andrikou I Grigoriou K Dima I Lazarou E Vlachopoulos C . Lipoprotein(a), metabolic profile and new-onset type 2 diabetes in patients with familial combined hyperlipidemia: a 9 year follow-up study. J Clin Lipidol. (2023) 17:512–8. doi: 10.1016/j.jacl.2023.05.103. PMID: 37321915

[27] Wang Y Zhang X Li Y Gui J Mei Y Yang X . Obesity- and lipid-related indices as a predictor of type 2 diabetes in a national cohort study. Front Endocrinol (Lausanne). (2023) 14:1331739. doi: 10.3389/fendo.2023.1331739. PMID: 38356678 PMC10864443

[28] Liang X Xing Z Li Y Gui S Hu H . Non-linear dose-response relationship between the visceral adiposity index and diabetes in adults with normoglycemia: a cohort study. Front Endocrinol (Lausanne). (2024) 15:1441878. doi: 10.3389/fendo.2024.1441878. PMID: 39698032 PMC11652130

[29] Fu X Zhao Y Wu Y Wen L Huo W Zhang D . Relationship between trajectory of Chinese visceral adiposity index and risk of type 2 diabetes mellitus: evidence from the China-PAR project. Diabetes Obes Metab. (2025) 27:785–94. doi: 10.1111/dom.16074. PMID: 39562295

[30] Zhang Y Gao W Li B Liu Y Tang X Yan L . The association between the visceral obesity indices and the future diabetes mellitus risk: a prospective cohort study. Diabetes Obes Metab. (2025) 27:4490–8. doi: 10.1111/dom.16492. PMID: 40432378

[31] Yu J Yi Q Chen G Hou L Liu Q Xu Y . The visceral adiposity index and risk of type 2 diabetes mellitus in China: a national cohort analysis. Diabetes Metab Res Rev. (2022) 38:e3507. doi: 10.1002/dmrr.3507. PMID: 34679251

[32] Wu J Gong L Li Q Hu J Zhang S Wang Y . A novel visceral adiposity index for prediction of type 2 diabetes and pre-diabetes in Chinese adults: a 5-year prospective study. Sci Rep. (2017) 7:13784. doi: 10.1038/s41598-017-14251-w. PMID: 29062099 PMC5653832

[33] Yang SH Yoon J Lee YJ Park B Jung DH . Lipid accumulation product index predicts new-onset type 2 diabetes among non-obese Koreans: a 12-year longitudinal study. Diabetes Metab Syndr Obes. (2022) 15:3729–37. doi: 10.2147/dmso.s389889. PMID: 36474727 PMC9719681

[34] Jin X Wei Y Mo Y Zhang Q Xu M Mai X . Associations of obesity and novel lipid indicators in the risk of type 2 diabetes mellitus in Chinese elderly hypertensive patients. Front Endocrinol (Lausanne). (2025) 16:1475323. doi: 10.3389/fendo.2025.1475323. PMID: 40235662 PMC11996637

[35] Wu Y Zhang Y Zhao Y Zhang X Gu M Huo W . Elevated lipid accumulation product trajectory patterns are associated with increasing incident risk of type 2 diabetes mellitus in China. Prev Med. (2025) 190:108186. doi: 10.1016/j.ypmed.2024.108186. PMID: 39612991

[36] Yu J Yi Q Hou L Chen G Shen Y Song Y . Transition of lipid accumulation product status and the risk of type 2 diabetes mellitus in middle-aged and older Chinese: a national cohort study. Front Endocrinol (Lausanne). (2021) 12:770200. doi: 10.3389/fendo.2021.770200. PMID: 34899605 PMC8660859

[37] Nusrianto R Ayundini G Kristanti M Astrella C Amalina N Muhadi . Visceral adiposity index and lipid accumulation product as a predictor of type 2 diabetes mellitus: the Bogor cohort study of non-communicable diseases risk factors. Diabetes Res Clin Pract. (2019) 155:107798. doi: 10.1016/j.diabres.2019.107798. PMID: 31330161

[38] Hafezi SG Saberi-Karimian M Ghasemi M Ghamsary M Moohebati M Esmaily H . Prediction of the 10-year incidence of type 2 diabetes mellitus based on advanced anthropometric indices using machine learning methods in the Iranian population. Diabetes Res Clin Pract. (2024) 214:111755. doi: 10.1016/j.diabres.2024.111755. PMID: 38936481

[39] Yang C Guo ZR Hu XS Zhou ZY Wu M . A prospective study on the association between lipid accumulation product or body mass index and diabetes. Zhonghua liu xing bing xue za zhi = Zhonghua liuxingbingxue zazhi. (2010) 31:5–8. 20302688

[40] Liu S Yu J Wang L Zhang X Wang F Zhu Y . Weight-adjusted waist index as a practical predictor for diabetes, cardiovascular disease, and non-accidental mortality risk. Nutr Metab Cardiovasc Dis. (2024) 34:2498–510. doi: 10.1016/j.numecd.2024.06.012. PMID: 39117486

[41] Chung TL Liu YH Wu PY Huang JC Chen SC . Sex difference in the associations among obesity-related indices with incidence of diabetes mellitus in a large Taiwanese population follow-up study. Front Public Health. (2023) 11:1094471. doi: 10.3389/fpubh.2023.1094471. PMID: 36741951 PMC9895090

[42] Liang X Lai K Li X Li Y Xing Z Gui S . Non-linear relationship between triglyceride glucose index and new-onset diabetes among individuals with non-alcoholic fatty liver disease: a cohort study. Lipids Health Dis. (2025) 24:94. doi: 10.1186/s12944-025-02518-5. PMID: 40089802 PMC11910846

[43] Zhang F Sun Y Bai Y Wu R Yang H . Association of triglyceride-glucose index and diabesity: evidence from a national longitudinal study. Lipids Health Dis. (2024) 23:412. doi: 10.1186/s12944-024-02403-7. PMID: 39707324 PMC11660576

[44] Shan Y Liu Q Gao T . Triglyceride-glucose index in predicting the risk of new-onset diabetes in the general population aged 45 years and older: a national prospective cohort study. BMC Endocr Disord. (2025) 25:25. doi: 10.1186/s12902-025-01848-w. PMID: 39865224 PMC11765927

[45] D’Elia L Rendina D Iacone R Strazzullo P Galletti F . Triglyceride-glucose index and new-onset type 2 diabetes mellitus in middle-aged men. Metabolites. (2025) 15. doi: 10.3390/metabo15080537 PMC12388705 40863155

[46] Xu M Huang M Qiang D Gu J Li Y Pan Y . Hypertriglyceridemic waist phenotype and lipid accumulation product: two comprehensive obese indicators of waist circumference and triglyceride to predict type 2 diabetes mellitus in Chinese population. J Diabetes Res. (2020) 2020:9157430. doi: 10.1155/2020/9157430. PMID: 33344653 PMC7725575

[47] Chen G Yi Q Hou L Peng S Fan M Song P . Transition of hypertriglyceridemic-waist phenotypes and the risk of type 2 diabetes mellitus among middle-aged and older Chinese: a national cohort study. Int J Environ Res Public Health. (2021) 18. doi: 10.3390/ijerph18073664. PMID: 33915915 PMC8037185

[48] Chen G Liu S Ouyang W Yang L Chen Y Guo X . Relationships between atherogenic index of plasma and body mass index with the risk of type 2 diabetes mellitus: insights from CHARLS. Acta Diabetol. (2025) 62:1775–85. doi: 10.1007/s00592-025-02516-0. PMID: 40332562

[49] Zhang YY Cao R Wan Q . Association between the plasma atherogenic index and type 2 diabetes in Chinese population: prospective cohort study based on 4C study. Front Endocrinol (Lausanne). (2025) 16:1571602. doi: 10.3389/fendo.2025.1571602. PMID: 40309448 PMC12040675

[50] Wang T Zhang M Shi W Li Y Zhang T Shi W . Atherogenic index of plasma, high sensitivity C-reactive protein and incident diabetes among middle-aged and elderly adults in China: a national cohort study. Cardiovasc Diabetol. (2025) 24:103. doi: 10.1186/s12933-025-02653-4 40045300 PMC11883954

[51] Yi Q Ren Z Bai G Zhu S Li S Li C . The longitudinal effect of the atherogenic index of plasma on type 2 diabetes in middle-aged and older Chinese. Acta Diabetol. (2022) 59:269–79. doi: 10.1007/s00592-021-01801-y 34648090

[52] Li YW Kao TW Chang PK Chen WL Wu LW . Atherogenic index of plasma as predictors for metabolic syndrome, hypertension and diabetes mellitus in Taiwan citizens: a 9-year longitudinal study. Sci Rep. (2021) 11:9900. doi: 10.1038/s41598-021-89307-z 33972652 PMC8110777

[53] Onat A Can G Kaya H Hergenç G . Atherogenic index of plasma (log10 triglyceride/high-density lipoprotein-cholesterol) predicts high blood pressure, diabetes, and vascular events. J Clin Lipidol. (2010) 4:89–98. doi: 10.1016/j.jacl.2010.02.005. PMID: 21122635

[54] Zou Y Lu S Li D Huang X Wang C Xie G . Exposure of cumulative atherogenic index of plasma and the development of prediabetes in middle-aged and elderly individuals: evidence from the CHARLS cohort study. Cardiovasc Diabetol. (2024) 23:355. doi: 10.1186/s12933-024-02449-y. PMID: 39350154 PMC11443941

[55] Wen L Wu Y Fu X Huo W Xu A Piao C . Atherogenic index of plasma and its 5-year changes associated with type 2 diabetes risk: a 10-year cohort study. Cardiovasc Diabetol. (2025) 24:349. doi: 10.1186/s12933-025-02903-5. PMID: 40855436 PMC12376724

[56] Jia X Lin H Ding Y Gu X Wang S Xu Y . Serum medium-chain fatty acids and the risk of incident diabetes: findings from the 4C study. J Clin Endocrinol Metab. (2025) 110:441–51. doi: 10.1210/clinem/dgae483. PMID: 39031583 PMC11747750

[57] Wang S Hu C Lin H Jia X Hu R Zheng R . Association of circulating long-chain free fatty acids and incident diabetes risk among normoglycemic Chinese adults: a prospective nested case-control study. Am J Clin Nutr. (2024) 120:336–46. doi: 10.1016/j.ajcnut.2024.05.003. PMID: 38729573

[58] Seah JYH Hong Y Cichońska A Sabanayagam C Nusinovici S Wong TY . Circulating metabolic biomarkers are consistently associated with type 2 diabetes risk in Asian and European populations. J Clin Endocrinol Metab. (2022) 107:e2751–61. doi: 10.1210/clinem/dgac212. PMID: 35390150

[59] Bragg F Kartsonaki C Guo Y Holmes M Du H Yu C . Circulating metabolites and the development of type 2 diabetes in Chinese adults. Diabetes Care. (2022) 45:477–80. doi: 10.2337/dc21-1415. PMID: 34848488 PMC7612375

[60] Villasanta-Gonzalez A Alcala-Diaz JF Vals-Delgado C Arenas AP Cardelo MP Romero-Cabrera JL . A plasma fatty acid profile associated to type 2 diabetes development: from the CORDIOPREV study. Eur J Nutr. (2022) 61:843–57. doi: 10.1007/s00394-021-02676-z. PMID: 34609622 PMC8854256

[61] Miao Z Lin JS Mao Y Chen GD Zeng FF Dong HL . Erythrocyte n-6 polyunsaturated fatty acids, gut microbiota, and incident type 2 diabetes: a prospective cohort study. Diabetes Care. (2020) 43:2435–43. doi: 10.2337/dc20-0631. PMID: 32723842 PMC7510039

[62] Seo IH Son DH Lee HS Lee YJ . Non-HDL cholesterol as a predictor for incident type 2 diabetes in community-dwelling adults: longitudinal findings over 12 years. Transl Res. (2022) 243:52–9. doi: 10.1016/j.trsl.2021.12.008. PMID: 34979322

[63] Janghorbani M Amini M . Utility of hypertriglyceridemic waist phenotype for predicting incident type 2 diabetes: the Isfahan Diabetes Prevention Study. J Diabetes Investig. (2016) 7:860–6. doi: 10.1111/jdi.12520. PMID: 27180654 PMC5089948

[64] Mora S Kamstrup PR Rifai N Nordestgaard BG Buring JE Ridker PM . Lipoprotein(a) and risk of type 2 diabetes. Clin Chem. (2010) 56:1252–60. doi: 10.1373/clinchem.2010.146779. PMID: 20511445 PMC2912456

[65] Jayedi A Soltani S Motlagh SZ Emadi A Shahinfar H Moosavi H . Anthropometric and adiposity indicators and risk of type 2 diabetes: systematic review and dose-response meta-analysis of cohort studies. Bmj. (2022) 376:e067516. doi: 10.1136/bmj-2021-067516. PMID: 35042741 PMC8764578

[66] Liu H Guo F Fu H Xu X Wang Z Kang J . Associations of triglyceride-glucose-related composite obesity indices with cardiovascular diseases and mortality: a systematic review and meta-analysis. Cardiovasc Diabetol. (2026). doi: 10.1186/s12933-026-03148-6. PMID: 41888845 PMC13141572

