# Supplementary material for: Lipid biomarkers for the prediction of type 2 diabetes risk, an umbrella review and updated meta-analyses of prospective observational studies
Source: Front Endocrinol (Lausanne). 2026 May 8;17:1784917. doi: 10.3389/fendo.2026.1784917 (PMC13194066; doi:10.3389/fendo.2026.1784917)
Supplement: Supplementary file 1 [file DataSheet1.docx]

**Fig. S1** Flow diagram of literature screening for the association between the risk of Type 2 Diabetes and Non-HDL-C.


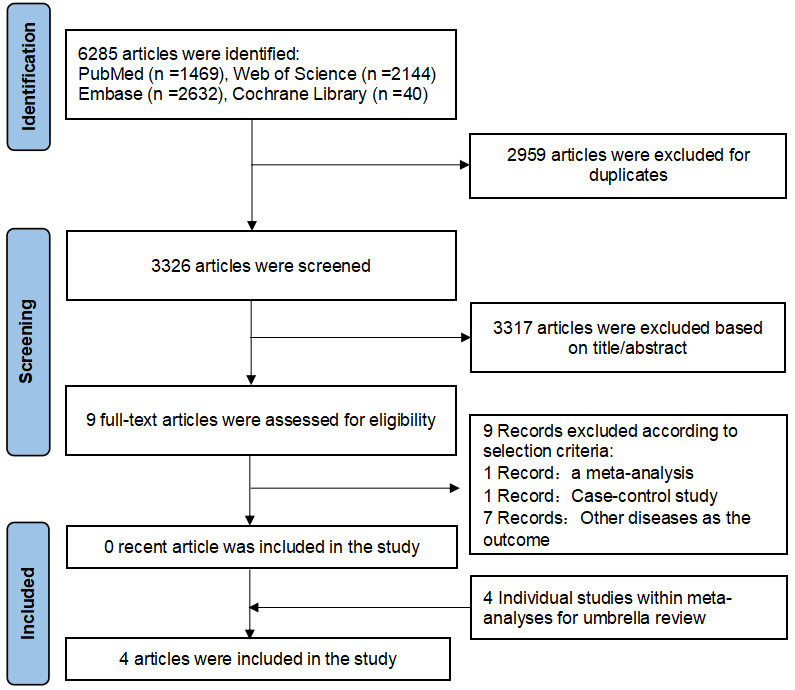


**Fig. S2** Flow diagram of literature screening for the association between the risk of Type 2 Diabetes and Lp(a).


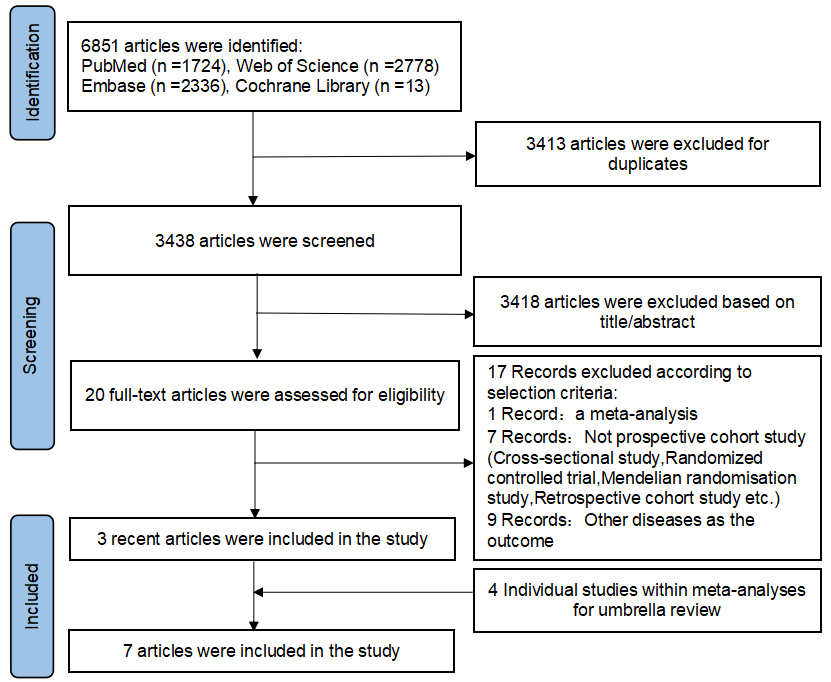


**Fig. S3** Flow diagram of literature screening for the association between the risk of Type 2 Diabetes and TG/HDL-C.


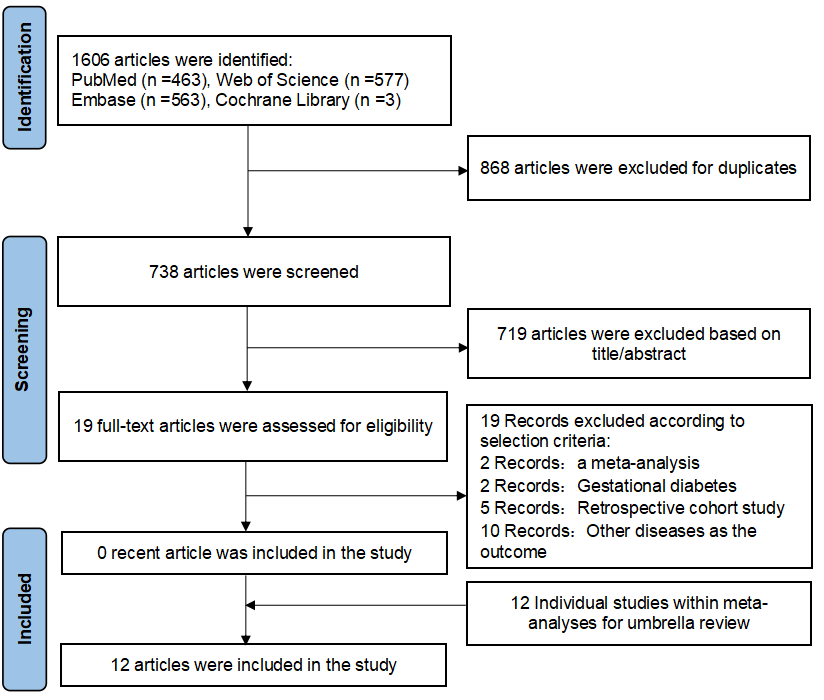


**Fig. S4** Flow diagram of literature screening for the association between the risk of Type 2 Diabetes and VAI.


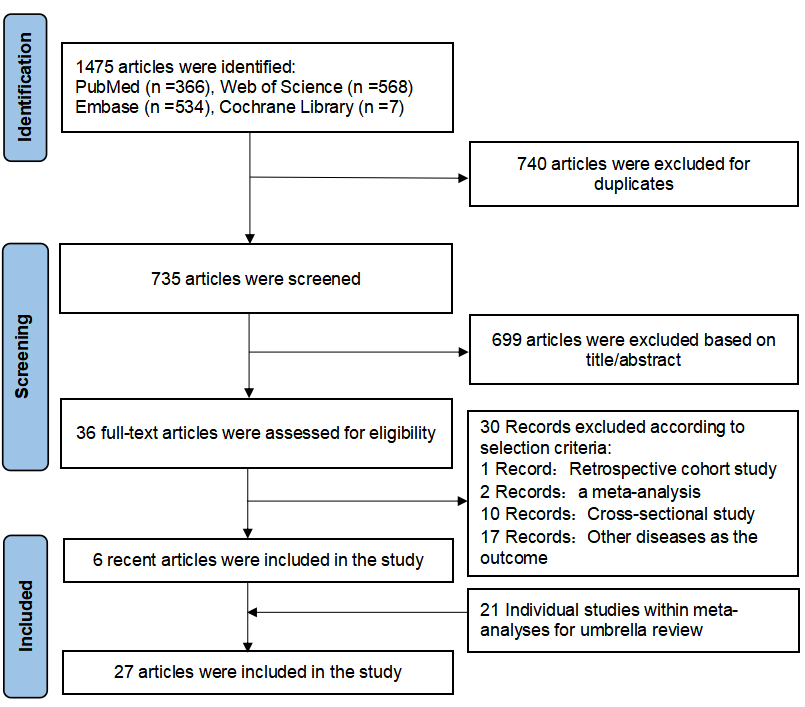


**Fig. S5** Flow diagram of literature screening for the association between the risk of Type 2 Diabetes and LAP.


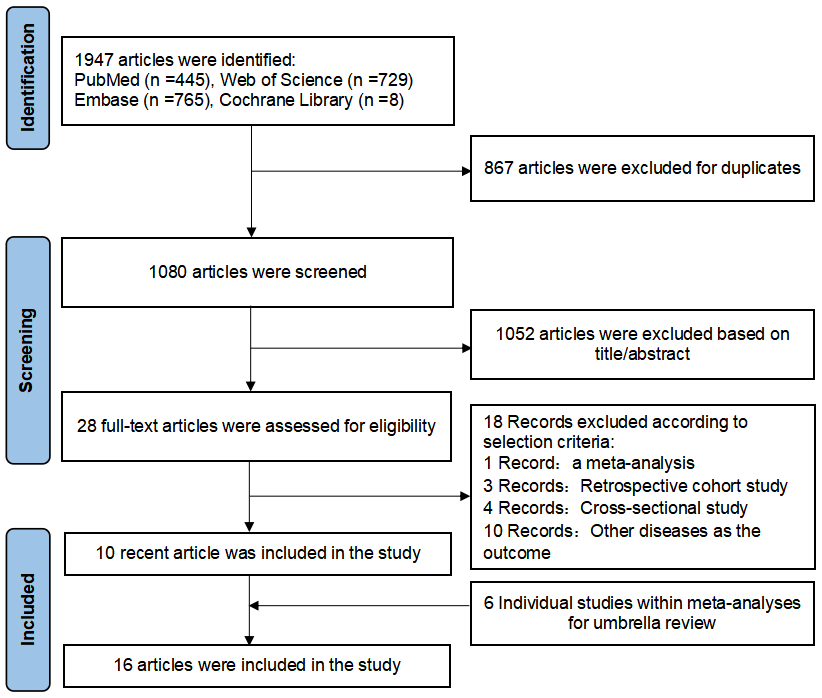


**Fig. S6** Flow diagram of literature screening for the association between the risk of Type 2 Diabetes and TyG.


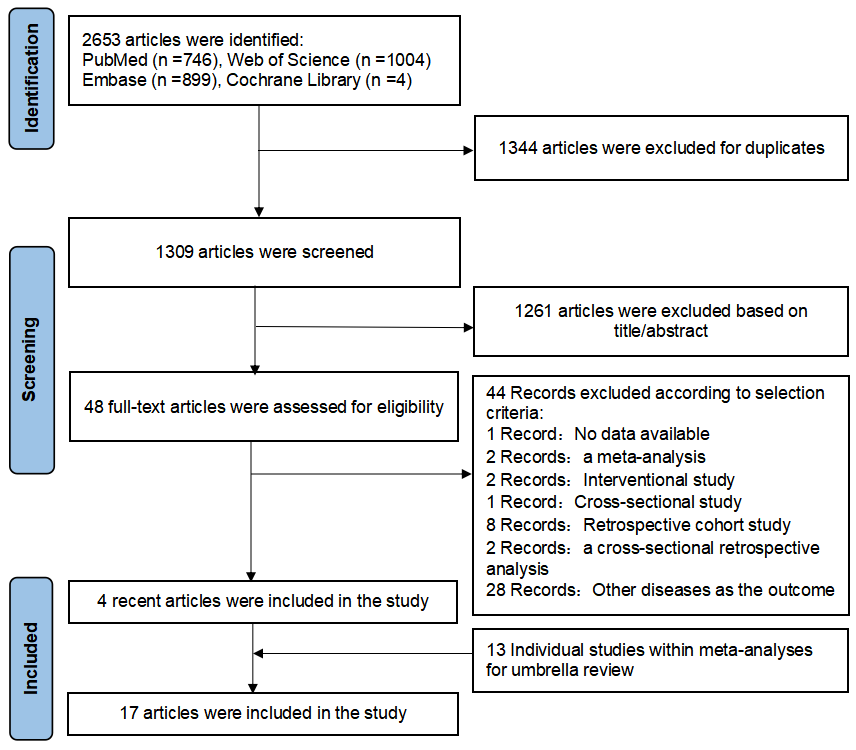


**Fig. S7** Flow diagram of literature screening for the association between the risk of Type 2 Diabetes and HTW.


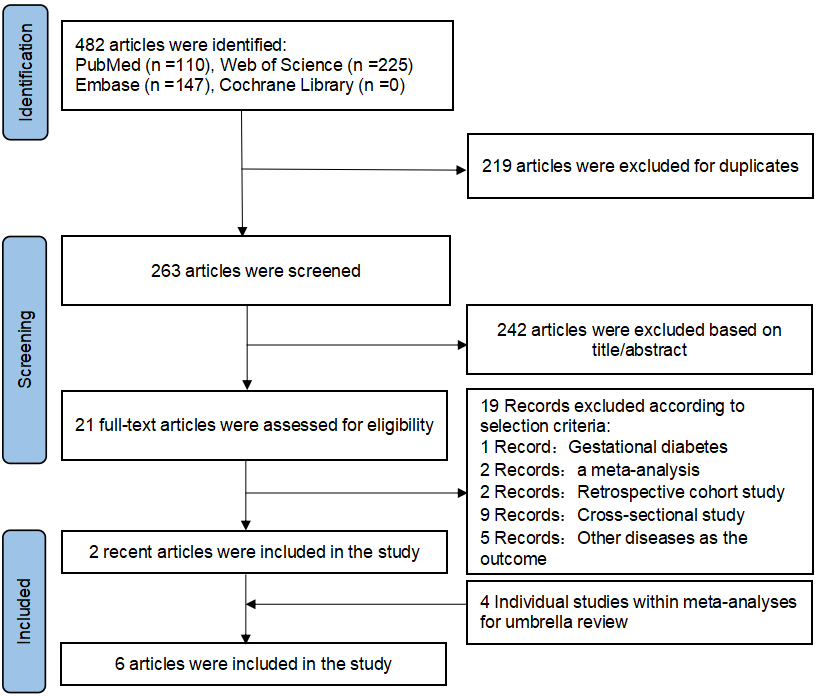


**Fig. S8** Flow diagram of literature screening for the association between the risk of Type 2 Diabetes and AIP.


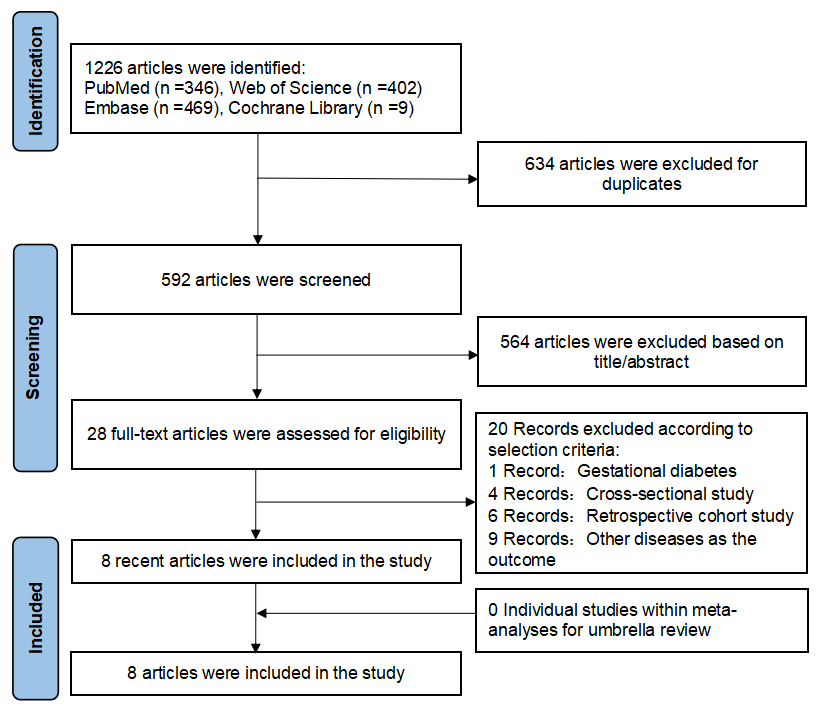


**Fig. S9** Flow diagram of literature screening for the association between the risk of Type 2 Diabetes and FAs.


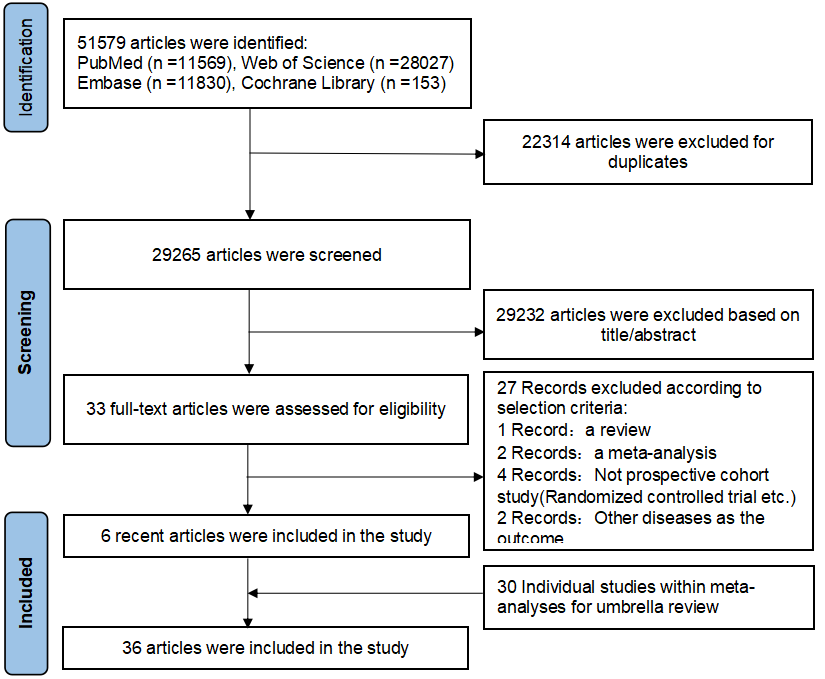


**Fig. S10** Forest plot for the association between the risk of Type 2 Diabetes and Non-HDL-C.

**Fig. S11** Sensitivity analysis of the association between the risk of Type 2 Diabetes and Non-HDL-C.

**Fig. S12** Forest plot for the association between the risk of Type 2 Diabetes and Lp(a).

**Fig. S13** Sensitivity analysis of the association between the risk of Type 2 Diabetes and Lp(a).

**Fig. S14a** Forest plot of subgroup analysis for the association between the risk of Type 2 Diabetes and Lp(a), stratified by population age groups.

**Fig. S14b** Forest plot of subgroup analysis for the association between the risk of Type 2 Diabetes and Lp(a), stratified by duration of follow-up.

**Fig. S15** Forest plot for the association between the risk of Type 2 Diabetes and TG/HDL-C.

**Fig. S16** Sensitivity analysis of the association between the risk of Type 2 Diabetes and TG/HDL-C.

**Fig. S17a** Forest plot of subgroup analysis for the association between the risk of Type 2 Diabetes and TG/HDL-C, stratified by population age groups.

**Fig. S17b** Forest plot of subgroup analysis for the association between the risk of Type 2 Diabetes and TG/HDL-C, stratified by duration of follow-up.

**Fig. S18** Funnel plot with Begg's and Egger's Tests for publication bias on the association between the risk of Type 2 Diabetes and Lipid metabolism indicators. [A] TG/HDL-C [B] VAI [C] LAP [D] TyG [E] AIP

B

C

D

E

A

**Fig. S19** Forest plot for the association between the risk of Type 2 Diabetes and VAI.

**Fig. S20** Sensitivity analysis of the association between the risk of Type 2 Diabetes and VAI.

**Fig. S21a** Forest plot of subgroup analysis for the association between the risk of Type 2 Diabetes and VAI, stratified by population age groups.

**Fig. S21b** Forest plot of subgroup analysis for the association between the risk of Type 2 Diabetes and VAI, stratified by duration of follow-up.

**Fig. S22** Forest plot for the association between the risk of Type 2 Diabetes and CVAI.

**Fig. S23** Sensitivity analysis of the association between the risk of Type 2 Diabetes and CVAI.

**Fig. S24** Forest plot for the association between the risk of Type 2 Diabetes and LAP.

**Fig. S25** Sensitivity analysis of the association between the risk of Type 2 Diabetes and LAP.

**Fig. S26** Forest plot of subgroup analysis for the association between the risk of Type 2 Diabetes and LAP, stratified by population age groups.

**Fig. S27** Forest plot for the association between the risk of Type 2 Diabetes and TyG.

**Fig. S28** Sensitivity analysis of the association between the risk of Type 2 Diabetes and TyG.

**Fig. S29a** Forest plot of subgroup analysis for the association between the risk of Type 2 Diabetes and TyG, stratified by population age groups.

**Fig. S29b** Forest plot of subgroup analysis for the association between the risk of Type 2 Diabetes and TyG, stratified by duration of follow-up.

**Fig. S30** Forest plot for the association between the risk of Type 2 Diabetes and HTW.

**Fig. S31** Sensitivity analysis of the association between the risk of Type 2 Diabetes and HTW.

**Fig. S32** Forest plot of subgroup analysis for the association between the risk of Type 2 Diabetes and HTW, stratified by population age groups.

**Fig. S33** Forest plot for the association between the risk of Type 2 Diabetes and AIP.

**Fig. S34** Sensitivity analysis of the association between the risk of Type 2 Diabetes and AIP.

**Fig. S35** Forest plot of subgroup analysis for the association between the risk of Type 2 Diabetes and AIP, stratified by population age groups.

**Fig. S36** Forest plot for the association between the risk of Type 2 Diabetes and SFAE.

**Fig. S37** Forest plot for the association between the risk of Type 2 Diabetes and SFAO.

**Fig. S38** Forest plot for the association between the risk of Type 2 Diabetes and SFAV.

**Fig. S39** Forest plot for the association between the risk of Type 2 Diabetes and MUFA.

**Fig. S40** Forest plot for the association between the risk of Type 2 Diabetes and n-3PUFA.

**Fig. S41** Forest plot for the association between the risk of Type 2 Diabetes and n-6PUFA.

**Fig. S41** (continued)

**Fig. S42** Forest plot for the association between the risk of Type 2 Diabetes and trans FA.
