# Supplementary material for: Lipid biomarkers for the prediction of type 2 diabetes risk, an umbrella review and updated meta-analyses of prospective observational studies
Source: Front Endocrinol (Lausanne). 2026 May 8;17:1784917. doi: 10.3389/fendo.2026.1784917 (PMC13194066; doi:10.3389/fendo.2026.1784917)
Supplement: Supplementary file 2 [file Table1.docx]

| **Supplementary Table S1. Formulas for Calculating Lipid Metabolism Indicators.** | |
| --- | --- |
| **Indicator Name** | **Calculation Formula** |
| Non-high-density lipoprotein cholesterol | non-HDL-C = TC - HDL-C |
| Triglyceride to high-density lipoprotein cholesterol ratio | TG/HDL-C Ratio = TG / HDL-C |
| Visceral Adiposity Index | Male: VAI = [WC / (39.68 + (1.88 × BMI))] × (TG / 1.03) × (1.31 / HDL-C) Female: VAI = [WC / (36.58 + (1.89 × BMI))] × (TG / 0.81) × (1.52 / HDL-C) |
| Chinese Visceral Adiposity Index | Male: CVAI = -267.93+ 0.68×Age + 0.03 × BMI + 4.00 × WC + 22.00 × lgTG - 16.32 × HDL-C Female: CVAI = -187.32 + 1.71 × Age + 4.23 × BMI + 1.12 × WC + 39.76 × lgTG - 11.66 × HDL-C |
| Lipid accumulation product | Male: LAP = (WC - 61.3) × TG  Female: LAP = (WC - 55.6) × TG |
| Triglyceride-Glucose Index | TyG Index = Ln [Fasting TG (mg/dL) × Fasting Glucose (mg/dL) / 2] |
| Atherogenic Index of Plasma | AIP = Log (TG / HDL-C) |

| **Supplementary Table S2. Search strategy for the meta analysis.** |
| --- |
| **Literature search strategy in PubMed** |
| #1 ("blood lipids"[Text Word] OR "dyslipidemia"[Text Word] OR "hyperlipidemia"[Text Word] OR "hypercholesterolemia"[Text Word] OR "hypertriglyceridemia"[Text Word] OR "Triglyceride"[Text Word] OR "total cholesterol"[Text Word] OR "HDL-C"[Text Word] OR "High-density lipoprotein cholesterol"[Text Word] OR "LDL-C"[Text Word] OR "Low-Density Lipoprotein Cholesterol"[Text Word] OR "ApoA1"[Text Word] OR "apolipoprotein a i"[Text Word] OR "APOB"[Text Word] OR "apolipoprotein b"[Text Word] OR "Lipoprotein A"[Text Word] OR "non-high-density lipoprotein cholesterol"[Text Word] OR "non-HDL"[Text Word] OR "Fatty Acids"[Text Word] OR "High-density Lipoprotein Cholesterol Ratio"[Text Word] OR "triglyceride to HDL cholesterol ratio"[Text Word] OR "Visceral Obesity Index"[Text Word] OR "Visceral adiposity index"[Text Word] OR "triglyceride-glucose index"[Text Word] OR "Hypertriglyceridemic waist phenotype"[Text Word] OR "Atherogenic Index of plasma"[Text Word]) |
| #2 ("meta-analysis"[Text Word] OR "systematic review"[Text Word]) |
| #3 ("diabetes mellitus type 2"[Text Word] OR "diabetes"[Text Word] OR "T2DM"[Text Word] OR "type 2 diabetes"[Text Word]) |
| #4 #1 AND #2 AND #3 |
| **Literature search strategy in Embase** |
| #1 ('blood lipids':ti,ab,kw OR 'dyslipidemia':ti,ab,kw OR 'hyperlipidemia':ti,ab,kw OR 'hypercholesterolemia':ti,ab,kw OR 'hypertriglyceridemia':ti,ab,kw OR 'triglyceride':ti,ab,kw OR 'total cholesterol':ti,ab,kw OR 'hdl-c':ti,ab,kw OR 'high-density lipoprotein cholesterol':ti,ab,kw OR 'ldl-c':ti,ab,kw OR 'low-density lipoprotein cholesterol':ti,ab,kw OR 'apoa1':ti,ab,kw OR 'apolipoprotein a i':ti,ab,kw OR 'apob':ti,ab,kw OR 'apolipoprotein b':ti,ab,kw OR 'lipoprotein a':ti,ab,kw OR 'non-high-density lipoprotein cholesterol':ti,ab,kw OR 'non-hdl':ti,ab,kw OR 'fatty acids':ti,ab,kw OR 'high-density lipoprotein cholesterol ratio':ti,ab,kw OR 'triglyceride to hdl cholesterol ratio':ti,ab,kw OR 'visceral obesity index':ti,ab,kw OR 'visceral adiposity index':ti,ab,kw OR 'triglyceride-glucose index':ti,ab,kw OR 'hypertriglyceridemic waist phenotype':ti,ab,kw OR 'atherogenic index of plasma':ti,ab,kw) |
| #2 ('meta-analysis':ti,ab,kw OR 'systematic review':ti,ab,kw) |
| #3 ('diabetes mellitus, type 2':ti,ab,kw OR 'diabetes':ti,ab,kw OR 't2dm':ti,ab,kw OR 'type 2 diabetes':ti,ab,kw) |
| #4 #1 AND #2 AND #3 |
| **Literature search strategy in Web of Science** |
| #1 ( (((((((((((((((((((((((((TS=("blood lipids")) OR TS=( "dyslipidemia" )) OR TS=("hyperlipidemia")) OR TS=( "hypercholesterolemia")) OR TS=("hypertriglyceridemia")) OR TS=("Triglyceride")) OR TS=("total cholesterol")) OR TS=( "HDL-C" )) OR TS=("High-density lipoprotein cholesterol" )) OR TS=("LDL-C")) OR TS=( "Low-Density Lipoprotein Cholesterol")) OR TS=( "ApoA1" )) OR TS=("apolipoprotein a i")) OR TS=( "APOB")) OR TS=( "apolipoprotein b" )) OR TS=("Lipoprotein A")) OR TS=( "non-high-density lipoprotein cholesterol")) OR TS=( "non-HDL" )) OR TS=("Fatty Acids" )) OR TS=("High-density Lipoprotein Cholesterol Ratio")) OR TS=( "triglyceride to HDL cholesterol ratio")) OR TS=( "Visceral Obesity Index")) OR TS=( "Visceral adiposity index" )) OR TS=("triglyceride-glucose index" )) OR TS=("Hypertriglyceridemic waist phenotype" )) OR TS=("Atherogenic Index of plasma") and Preprint Citation Index (Exclude – Database) ) |
| #2 ( (TS=("meta-analysis")) OR TS=( "systematic review") and Preprint Citation Index (Exclude – Database) ) |
| #3 ( (((TS=("diabetes mellitus type 2" )) OR TS=("diabetes")) OR TS=("T2DM" )) OR TS=("type 2 diabetes") and Preprint Citation Index (Exclude – Database) ) |
| #4 #1 AND #2 AND #3 |
| **Literature search strategy in Cochrane Library** |
| #1 (("blood lipids"):ti,ab,kw OR ("dyslipidemia"):ti,ab,kw OR ("hyperlipidemia"):ti,ab,kw OR ("hypercholesterolemia"):ti,ab,kw OR ("hypertriglyceridemia"):ti,ab,kw OR ("Triglyceride"):ti,ab,kw OR ("total cholesterol"):ti,ab,kw OR ("HDL-C"):ti,ab,kw OR ("High-density lipoprotein cholesterol"):ti,ab,kw OR ("LDL-C"):ti,ab,kw OR ("Low-Density Lipoprotein Cholesterol"):ti,ab,kw OR ("ApoA1"):ti,ab,kw OR ("apolipoprotein a i"):ti,ab,kw OR ("APOB"):ti,ab,kw OR ("apolipoprotein b"):ti,ab,kw OR ("Lipoprotein A"):ti,ab,kw OR ("non-high-density lipoprotein cholesterol"):ti,ab,kw OR ("non-HDL"):ti,ab,kw OR ("Fatty Acids"):ti,ab,kw OR ("High-density Lipoprotein Cholesterol Ratio"):ti,ab,kw OR ("triglyceride to HDL cholesterol ratio"):ti,ab,kw OR ("Visceral Obesity Index"):ti,ab,kw OR ("Visceral adiposity index"):ti,ab,kw OR ("triglyceride-glucose index"):ti,ab,kw OR ("Hypertriglyceridemic waist phenotype"):ti,ab,kw OR ("Atherogenic Index of plasma"):ti,ab,kw) |
| #2 (("meta-analysis"):ti,ab,kw OR ("Systematic Review"):ti,ab,kw) |
| #3 (("diabetes mellitus, type 2"):ti,ab,kw OR ("diabetes"):ti,ab,kw OR ("T2DM"):ti,ab,kw OR ("type 2 diabetes"):ti,ab,kw) |
| #4 #1 AND #2 AND #3 |

| **Supplementary Table S3. Search strategy for primary studies on Lp(a).** |
| --- |
| **Literature search strategy in PubMed** |
| #1 ((Lipoprotein A[Text Word]) AND ("diabetes mellitus type 2"[Text Word] OR "diabetes"[Text Word] OR "T2DM"[Text Word] OR "type 2 diabetes"[Text Word])) |
| **Literature search strategy in Embase** |
| #1 ('lipoprotein a':ab,ti) |
| #2 ('diabetes mellitus type 2':ab,ti OR 'diabetes':ab,ti OR 't2dm':ab,ti OR 'type 2 diabetes':ab,ti) |
| #3 #1 AND #2 |
| **Literature search strategy in Web of Science** |
| #1 ( TS=("Lipoprotein A") and Preprint Citation Index (Exclude – Database) and Research Commons (Exclude – Database) ) |
| #2 ( (((TS=("diabetes mellitus type 2")) OR TS=("diabetes")) OR TS=("T2DM")) OR TS=("type 2 diabetes") and Preprint Citation Index (Exclude – Database) and Research Commons (Exclude – Database) ) |
| #3 #1 AND #2 |
| **Literature search strategy in Cochrane Library** |
| #1 (("Lipoprotein A"):ti,ab,kw) |
| #2 (("diabetes mellitus type 2"):ti,ab,kw AND ("diabetes"):ti,ab,kw AND ("T2DM"):ti,ab,kw AND ("type 2 diabetes"):ti,ab,kw) |
| #3 #1 AND #2 |

| **Supplementary Table S4. Search strategy for primary studies on Non-HDL-C.** |
| --- |
| **Literature search strategy in PubMed** |
| #1 ((("non-high-density lipoprotein cholesterol"[Text Word]) OR ("non-HDL"[Text Word])) AND ("diabetes mellitus type 2"[Text Word] OR "diabetes"[Text Word] OR "T2DM"[Text Word] OR "type 2 diabetes"[Text Word])) |
| **Literature search strategy in Embase** |
| #1 ( 'non-high-density lipoprotein cholesterol':ti,ab,kw OR 'non-hdl':ti,ab,kw) |
| #2 ( 'diabetes mellitus type 2':ti,ab,kw OR 'diabetes':ti,ab,kw OR 't2dm':ti,ab,kw OR 'type 2 diabetes':ti,ab,kw) |
| #3 #1 AND #2 |
| **Literature search strategy in Web of Science** |
| #1 ( (TS=("non-high-density lipoprotein cholesterol")) OR TS=("non-HDL") and Preprint Citation Index (Exclude – Database) and Research Commons (Exclude – Database) ) |
| #2 ( (((TS=("diabetes mellitus type 2")) OR TS=("diabetes")) OR TS=("T2DM")) OR TS=("type 2 diabetes") and Preprint Citation Index (Exclude – Database) and Research Commons (Exclude – Database) ) |
| #3 #1 AND #2 |
| **Literature search strategy in Cochrane Library** |
| #1 ( ("non-high-density lipoprotein cholesterol"):ti,ab,kw OR ("non-HDL"):ti,ab,kw) |
| #2 (("diabetes mellitus type 2"):ti,ab,kw AND ("diabetes"):ti,ab,kw AND ("T2DM"):ti,ab,kw AND ("type 2 diabetes"):ti,ab,kw) |
| #3 #1 AND #2 |

| **Supplementary Table S5. Search strategy for primary studies on TG/HDL-C.** |
| --- |
| **Literature search strategy in PubMed** |
| #1 ("High-density Lipoprotein Cholesterol Ratio"[Text Word] OR "triglyceride to HDL cholesterol ratio"[Text Word]) |
| #2 ("diabetes mellitus type 2"[Text Word] OR "diabetes"[Text Word] OR "T2DM"[Text Word] OR "type 2 diabetes"[Text Word]) |
| #3 #1 AND #2 |
| **Literature search strategy in Embase** |
| #1 ('high-density lipoprotein cholesterol ratio':ti,ab,kw OR 'triglyceride to hdl cholesterol ratio':ti,ab,kw) |
| #2 ('diabetes mellitus type 2':ti,ab,kw OR 'diabetes':ti,ab,kw OR 't2dm':ti,ab,kw OR 'type 2 diabetes':ti,ab,kw) |
| #3 #1 AND #2 |
| **Literature search strategy in Web of Science** |
| #1 ( (TS=("High-density Lipoprotein Cholesterol Ratio")) OR TS=("triglyceride to HDL cholesterol ratio") and Preprint Citation Index (Exclude – Database) and Research Commons (Exclude – Database) ) |
| #2 ( (((TS=("diabetes mellitus type 2")) OR TS=("diabetes")) OR TS=("T2DM")) OR TS=("type 2 diabetes") and Preprint Citation Index (Exclude – Database) and Research Commons (Exclude – Database) ) |
| #3 #1 AND #2 |
| **Literature search strategy in Cochrane Library** |
| #1 (("High-density Lipoprotein Cholesterol Ratio"):ti,ab,kw OR ("triglyceride to HDL cholesterol ratio"):ti,ab,kw) |
| #2 (("diabetes mellitus type 2"):ti,ab,kw AND ("diabetes"):ti,ab,kw AND ("T2DM"):ti,ab,kw AND ("type 2 diabetes"):ti,ab,kw) |
| #3 #1 AND #2 |

| **Supplementary Table S6. Search strategy for primary studies on VAI.** |
| --- |
| **Literature search strategy in PubMed** |
| #1 ("Visceral Obesity Index"[Text Word] OR "Visceral adiposity index"[Text Word]) |
| #2 ("diabetes mellitus type 2"[Text Word] OR "diabetes"[Text Word] OR "T2DM"[Text Word] OR "type 2 diabetes"[Text Word]) |
| #3 #1 AND #2 |
| **Literature search strategy in Embase** |
| #1 ('visceral obesity index':ti,ab,kw OR 'visceral adiposity index':ti,ab,kw ) |
| #2 ('diabetes mellitus type 2':ti,ab,kw OR 'diabetes':ti,ab,kw OR 't2dm':ti,ab,kw OR 'type 2 diabetes':ti,ab,kw) |
| #3 #1 AND #2 |
| **Literature search strategy in Web of Science** |
| #1 ( (((TS=("diabetes mellitus type 2")) OR TS=("diabetes")) OR TS=("T2DM")) OR TS=("type 2 diabetes") and Preprint Citation Index (Exclude – Database) and Research Commons (Exclude – Database) ) |
| #2 ( (TS=("Visceral Obesity Index")) OR TS=("Visceral adiposity index") and Preprint Citation Index (Exclude – Database) and Research Commons (Exclude – Database) ) |
| #3 #1 AND #2 |
| **Literature search strategy in Cochrane Library** |
| #1 (("Visceral adiposity index"):ti,ab,kw OR ("Visceral Obesity Index"):ti,ab,kw) |
| #2 (("diabetes mellitus type 2"):ti,ab,kw AND ("diabetes"):ti,ab,kw AND ("T2DM"):ti,ab,kw AND ("type 2 diabetes"):ti,ab,kw) |
| #3 #1 AND #2 |

| **Supplementary Table S7. Search strategy for primary studies on LAP.** |
| --- |
| **Literature search strategy in PubMed** |
| #1 (lipid accumulation product[Text Word]) OR (LAP[Text Word]) |
| #2 "diabetes mellitus type 2"[Text Word] OR "diabetes"[Text Word] OR "T2DM"[Text Word] OR "type 2 diabetes"[Text Word] |
| #3 #1 AND #2 |
| **Literature search strategy in Embase** |
| #1 ('lipid accumulation product':ti,ab,kw OR 'lap':ti,ab,kw) |
| #2 ('diabetes mellitus type 2':ti,ab,kw OR 'diabetes':ti,ab,kw OR 't2dm':ti,ab,kw OR 'type 2 diabetes':ti,ab,kw) |
| #3 #1 AND #2 |
| **Literature search strategy in Web of Science** |
| #1 ( (TS=("lipid accumulation product")) OR TS=(LAP) Editions: WOS.IC,WOS.CCR,WOS.SCI,WOS.AHCI,WOS.ESCI,WOS.ISTP,WOS.SSCI,GRANTS.GRANTS,KJD.KJD,MEDLINE.MEDLINE,PQDT.PQDT,SCIELO.SCIELO ) |
| #2 ( (((TS=("diabetes mellitus type 2")) OR TS=("diabetes")) OR TS=("T2DM")) OR TS=("type 2 diabetes") Editions: WOS.IC,WOS.CCR,WOS.SCI,WOS.AHCI,WOS.ESCI,WOS.ISTP,WOS.SSCI,GRANTS.GRANTS,KJD.KJD,MEDLINE.MEDLINE,PQDT.PQDT,SCIELO.SCIELO ) |
| #3 #1 AND #2 |
| **Literature search strategy in Cochrane Library** |
| #1 ("lipid accumulation product"):ti,ab,kw OR ("LAP"):ti,ab,kw (Word variations have been searched) |
| #2 ("diabetes mellitus type 2"):ti,ab,kw AND ("diabetes"):ti,ab,kw AND ("T2DM"):ti,ab,kw AND ("type 2 diabetes"):ti,ab,kw (Word variations have been searched) |
| #3 #1 AND #2 |

| **Supplementary Table S8. Search strategy for primary studies on TyG.** |
| --- |
| **Literature search strategy in PubMed** |
| #1 (("triglyceride-glucose index"[Text Word]) AND ("diabetes mellitus type 2"[Text Word] OR "diabetes"[Text Word] OR "T2DM"[Text Word] OR "type 2 diabetes"[Text Word])) |
| **Literature search strategy in Embase** |
| #1 ('triglyceride-glucose index':ti,ab,kw) |
| #2 ('diabetes mellitus type 2':ti,ab,kw OR 'diabetes':ti,ab,kw OR 't2dm':ti,ab,kw OR 'type 2 diabetes':ti,ab,kw) |
| #3 #1 AND #2 |
| **Literature search strategy in Web of Science** |
| #1 ( (((TS=("diabetes mellitus type 2")) OR TS=("diabetes")) OR TS=("T2DM")) OR TS=("type 2 diabetes") and Preprint Citation Index (Exclude – Database) and Research Commons (Exclude – Database) ) |
| #2 ( TS=("triglyceride-glucose index") and Preprint Citation Index (Exclude – Database) and Research Commons (Exclude – Database) ) |
| #3 #1 AND #2 |
| **Literature search strategy in Cochrane Library** |
| #1 (("triglyceride-glucose index"):ti,ab,kw) |
| #2 (("diabetes mellitus type 2"):ti,ab,kw AND ("diabetes"):ti,ab,kw AND ("T2DM"):ti,ab,kw AND ("type 2 diabetes"):ti,ab,kw) |
| #3 #1 AND #2 |

| **Supplementary Table S9. Search strategy for primary studies on HTW.** |
| --- |
| **Literature search strategy in PubMed** |
| #1 (("Hypertriglyceridemic waist "[Text Word]) AND ("diabetes mellitus type 2"[Text Word] OR "diabetes"[Text Word] OR "T2DM"[Text Word] OR "type 2 diabetes"[Text Word])) |
| **Literature search strategy in Embase** |
| #1 ('hypertriglyceridemic waist':ti,ab,kw) |
| #2 ('diabetes mellitus type 2':ti,ab,kw OR 'diabetes':ti,ab,kw OR 't2dm':ti,ab,kw OR 'type 2 diabetes':ti,ab,kw) |
| #3 #1 AND #2 |
| **Literature search strategy in Web of Science** |
| #1 ( (((TS=("diabetes mellitus type 2")) OR TS=("diabetes")) OR TS=("T2DM")) OR TS=("type 2 diabetes") and Preprint Citation Index (Exclude – Database) and Research Commons (Exclude – Database) ) |
| #2 ( TS=(Hypertriglyceridemic waist) and Preprint Citation Index (Exclude – Database) and Research Commons (Exclude – Database) ) |
| #3 #1 AND #2 |
| **Literature search strategy in Cochrane Library** |
| #1 (("Hypertriglyceridemic waist"):ti,ab,kw) |
| #2 (("diabetes mellitus type 2"):ti,ab,kw AND ("diabetes"):ti,ab,kw AND ("T2DM"):ti,ab,kw AND ("type 2 diabetes"):ti,ab,kw) |
| #3 #1 AND #2 |

| **Supplementary Table S10. Search strategy for primary studies on AIP.** |
| --- |
| **Literature search strategy in PubMed** |
| #1 (("Atherogenic Index of plasma"[Text Word]) AND ("diabetes mellitus type 2"[Text Word] OR "diabetes"[Text Word] OR "T2DM"[Text Word] OR "type 2 diabetes"[Text Word])) |
| **Literature search strategy in Embase** |
| #1 ('atherogenic index of plasma':ti,ab,kw) |
| #2 ( 'diabetes mellitus type 2':ti,ab,kw OR 'diabetes':ti,ab,kw OR 't2dm':ti,ab,kw OR 'type 2 diabetes':ti,ab,kw) |
| #3 #1 AND #2 |
| **Literature search strategy in Web of Science** |
| #1 ( (((TS=("diabetes mellitus type 2")) OR TS=("diabetes")) OR TS=("T2DM")) OR TS=("type 2 diabetes") and Preprint Citation Index (Exclude – Database) and Research Commons (Exclude – Database) ) |
| #2 ( TS=("Atherogenic Index of plasma") and Preprint Citation Index (Exclude – Database) and Research Commons (Exclude – Database) ) |
| #3 #1 AND #2 |
| **Literature search strategy in Cochrane Library** |
| #1 (("Atherogenic Index of plasma"):ti,ab,kw) |
| #2 (("diabetes mellitus type 2"):ti,ab,kw AND ("diabetes"):ti,ab,kw AND ("T2DM"):ti,ab,kw AND ("type 2 diabetes"):ti,ab,kw) |
| #3 #1 AND #2 |

| **Supplementary Table S11. Search strategy for primary studies on FAs.** |
| --- |
| **Literature search strategy in PubMed** |
| #1 ("Fatty Acids"[Text Word] AND ("diabetes mellitus type 2"[Text Word] OR "diabetes"[Text Word] OR "T2DM"[Text Word] OR "type 2 diabetes"[Text Word])) |
| #2 ("controlled"[Text Word] OR "randomized"[Text Word] OR "RCT"[Text Word] OR "case report"[Text Word] OR "cross-sectional study"[Text Word] OR "mendelian randomization study"[Text Word] OR "retrospective study"[Text Word] OR "case series"[Text Word] OR "clinical trial"[Text Word] OR "rodent"[Title] OR "mice"[Title] OR "rat"[Title] OR "rats"[Title] OR "cell"[Title] OR "mouse"[Title] OR "Inhibitors"[Title]) |
| #3 #1 AND #2 |
| **Literature search strategy in Embase** |
| #1 ('fatty acids':ti,ab,kw) |
| #2 ('diabetes mellitus type 2':ti,ab,kw OR 'diabetes':ti,ab,kw OR 't2dm':ti,ab,kw OR 'type 2 diabetes':ti,ab,kw) |
| #3 #1 AND #2 |
| #4 ('controlled':ti,ab,kw OR 'randomized':ti,ab,kw OR 'rct':ti,ab,kw OR 'case report':ti,ab,kw OR 'cross-sectional study':ti,ab,kw OR 'mendelian randomization study':ti,ab,kw OR 'retrospective study':ti,ab,kw OR 'case series':ti,ab,kw OR 'clinical trial':ti,ab,kw) |
| #5 ('rodent':ti OR 'mice':ti OR 'rat':ti OR 'rats':ti OR 'cell':ti OR 'mouse':ti OR 'inhibitors':ti) |
| #6 #4 OR #5 |
| #7 #3 NOT #6 |
| **Literature search strategy in Web of Science** |
| #1 ( (((TS=("diabetes mellitus type 2")) OR TS=("diabetes")) OR TS=("T2DM")) OR TS=("type 2 diabetes") and Preprint Citation Index (Exclude – Database) and Research Commons (Exclude – Database) ) |
| #2 ( TS=("Fatty Acids") and Preprint Citation Index (Exclude – Database) and Research Commons (Exclude – Database) ) |
| #3 #1 AND #2 |
| #4 ( TS=("Fatty Acids") and Preprint Citation Index (Exclude – Database) and Research Commons (Exclude – Database) ) |
| #5 #3 NOT #4 |
| **Literature search strategy in Cochrane Library** |
| #1 (("Fatty Acids"):ti,ab,kw) |
| #2 (("diabetes mellitus type 2"):ti,ab,kw AND ("diabetes"):ti,ab,kw AND ("T2DM"):ti,ab,kw AND ("type 2 diabetes"):ti,ab,kw) |
| #3 #1 AND #2 |

| **Supplementary Table S12. Criteria for quality of evidence classification in observational studies** | |
| --- | --- |
| **Category** | **Criteria** |
| Convincing, class I | ● > 1000 cases or (more than 20000 participants for continuous outcomes) ● *P* < 1×10^-6^ ● *I^2^* < 50% ● No small study effect ● Prediction interval excludes null value ● No excess significance bias |
| Highly suggestive, classⅡ | ● > 1000 cases or (more than 20000 participants for continuous outcomes) ● *P* < 1×10^-6^ ● *P* < 0.05 of largest study in meta-analysis |
| Suggestive, class Ⅲ | ● >1000 cases or (more than 20000 participants for continuous outcomes) ● *P* < 1×10^-3^ |
| Weak, class IV | ● *P* < 0.05 |
| Not significant | ● *P* > 0.05 |

| **Supplementary Table S13. List of Excluded Studies for the meta-analyses.** |
| --- |
| **Data is not published publicly (n = 2)** |
| 1. Qian F, Ardisson Korat AV, Imamura F, Marklund M, Tintle N, Virtanen JK, Zhou X, Bassett JK, Lai H, Hirakawa Y et al: n-3 Fatty Acid Biomarkers and Incident Type 2 Diabetes: An Individual Participant-Level Pooling Project of 20 Prospective Cohort Studies. Diabetes Care 2021, 44(5):1133-1142. |
| 2. Wu JHY, Marklund M, Imamura F, Tintle N, Ardisson Korat AV, de Goede J, Zhou X, Yang WS, de Oliveira Otto MC, Kröger J et al: Omega-6 fatty acid biomarkers and incident type 2 diabetes: pooled analysis of individual-level data for 39 740 adults from 20 prospective cohort studies. Lancet Diabetes Endocrinol 2017, 5(12):965-974. |
| **Gestational diabetes as the outcome (n = 2)** |
| 1. Hu J, Gillies CL, Lin S, Stewart ZA, Melford SE, Abrams KR, Baker PN, Khunti K, Tan BK: Association of maternal lipid profile and gestational diabetes mellitus: A systematic review and meta-analysis of 292 studies and 97,880 women. EClinicalMedicine 2021, 34:100830. |
| 2. Song T, Su G, Chi Y, Wu T, Xu Y, Chen C: Triglyceride-glucose index predicts the risk of gestational diabetes mellitus: a systematic review and meta-analysis. Gynecol Endocrinol 2022, 38(1):10-15. |
| **Interventional study (n = 8)** |
| 1. Gaeini Z, Bahadoran Z, Mirmiran P: Saturated Fatty Acid Intake and Risk of Type 2 Diabetes: An Updated Systematic Review and Dose-Response Meta-Analysis of Cohort Studies. Adv Nutr 2022, 13(6):2125-2135 |
| 2. Chen C, Yang Y, Yu X, Hu S, Shao S: Association between omega-3 fatty acids consumption and the risk of type 2 diabetes: A meta-analysis of cohort studies. J Diabetes Investig 2017, 8(4):480-488. |
| 3. Neuenschwander M, Barbaresko J, Pischke CR, Iser N, Beckhaus J, Schwingshackl L, Schlesinger S: Intake of dietary fats and fatty acids and the incidence of type 2 diabetes: A systematic review and dose-response meta-analysis of prospective observational studies. PLoS Med 2020, 17(12):e1003347. |
| 4. Wu JH, Micha R, Imamura F, Pan A, Biggs ML, Ajaz O, Djousse L, Hu FB, Mozaffarian D: Omega-3 fatty acids and incident type 2 diabetes: a systematic review and meta-analysis. Br J Nutr 2012, 107 Suppl 2(0 2):S214-227. |
| 5. Zhou Y, Tian C, Jia C: Association of fish and n-3 fatty acid intake with the risk of type 2 diabetes: A meta-analysis of prospective studies. British Journal of Nutrition 2012, 108(3):408-417. |
| 6. Namazi N, Brett NR, Bellissimo N, Larijani B, Heshmati J, Azadbakht L: The association between types of seafood intake and the risk of type 2 diabetes: a systematic review and meta-analysis of prospective cohort studies. Health Promot Perspect 2019, 9(3):164-173. |
| 7. Wang S, Cai R, Yuan Y, Varghese Z, Moorhead J, Ruan XZ: Association between reductions in low-density lipoprotein cholesterol with statin therapy and the risk of new-onset diabetes: a meta-analysis. Sci Rep 2017, 7:39982. |
| 8. Delpino FM, Figueiredo LM, da Silva BGC, da Silva TG, Mintem GC, Bielemann RM, Gigante DP: Omega-3 supplementation and diabetes: A systematic review and meta-analysis. Crit Rev Food Sci Nutr 2022, 62(16):4435-4448. |
| **Not a meta-analysis (n = 3)** |
| 1. Nusrianto R, Tahapary DL, Soewondo P: Visceral adiposity index as a predictor for type 2 diabetes mellitus in Asian population: A systematic review. Diabetes and Metabolic Syndrome: Clinical Research and Reviews 2019, 13(2):1231-1235. |
| 2. Liu W, Zhang G, Nie Z, Guan X, Sun T, Jin X, Li B: Low Concentration of Lipoprotein(a) is an Independent Predictor of Incident Type 2 Diabetes. Horm Metab Res 2024, 56(7):504-508. |
| 3. Forouhi NG, Imamura F, Sharp SJ, Koulman A, Schulze MB, Zheng J, Ye Z, Sluijs I, Guevara M, Huerta JM et al: Association of Plasma Phospholipid n-3 and n-6 Polyunsaturated Fatty Acids with Type 2 Diabetes: The EPIC-InterAct Case-Cohort Study. PLoS Med 2016, 13(7):e1002094 |
| **Case-control study (n = 2)** |
| 1. Zhu XW, Deng FY, Lei SF: Meta-analysis of Atherogenic Index of Plasma and other lipid parameters in relation to risk of type 2 diabetes mellitus. Primary Care Diabetes 2015, 9(1):60-67. |
| 2. Ma MY, Li KL, Zheng H, Dou YL, Han LY, Wang L: Omega-3 index and type 2 diabetes: Systematic review and meta-analysis. Prostaglandins Leukot Essent Fatty Acids 2021, 174:102361. |
| **Randomized clinical trial (n = 1)** |
| 1. Xiao Y, Zhang Q, Liao X, Elbelt U, Weylandt KH: The effects of omega-3 fatty acids in type 2 diabetes: A systematic review and meta-analysis. Prostaglandins Leukot Essent Fatty Acids 2022, 182:102456. |
| **Other diseases as the outcome (n = 14)** |
| 1. Tian X, Zhang N, Tse G, Li G, Sun Y, Liu T: Association between lipoprotein(a) and premature atherosclerotic cardiovascular disease: a systematic review and meta-analysis. Eur Heart J Open 2024, 4(3):oeae031. |
| 2. Ulloque-Badaracco JR, Al-Kassab-Córdova A, Hernandez-Bustamante EA, Alarcon-Braga EA, Huayta-Cortez M, Carballo-Tello XL, Seminario-Amez RA, Herrera-Añazco P, Benites-Zapata VA: Association of apolipoproteins and lipoprotein(a) with metabolic syndrome: a systematic review and meta-analysis. Lipids Health Dis 2023, 22(1):98. |
| 3. Li Z, Yuan Y, Qi Q, Wang Q, Feng L: Relationship between dyslipidemia and diabetic retinopathy in patients with type 2 diabetes mellitus: a systematic review and meta-analysis. Syst Rev 2023, 12(1):148. |
| 4. Chen Y, Chang Z, Liu Y, Zhao Y, Fu J, Zhang Y, Liu Y, Fan Z: Triglyceride to high-density lipoprotein cholesterol ratio and cardiovascular events in the general population: A systematic review and meta-analysis of cohort studies. Nutr Metab Cardiovasc Dis 2022, 32(2):318-329. |
| 5. Ulloque-Badaracco JR, Mosquera-Rojas MD, Hernandez-Bustamante EA, Alarcón-Braga EA, Ulloque-Badaracco RR, Al-Kassab-Córdova A, Herrera-Añazco P, Benites-Zapata VA, Hernandez AV: Association between Lipid Profile and Apolipoproteins with Risk of Diabetic Foot Ulcer: A Systematic Review and Meta-Analysis. Int J Clin Pract 2022, 2022:5450173 |
| 6. Deng S, Peng L: Triglyceride Glucose Index and the Risk of Diabetic Nephropathy in Patients with Type 2 Diabetes: A Meta-Analysis. Horm Metab Res 2025, 57(2):106-116. |
| 7. Mardi P, Abdi F, Ehsani A, Seif E, Djalalinia S, Heshmati J, Shahrestanaki E, Gorabi AM, Qorbani M: Is non-high-density lipoprotein associated with metabolic syndrome? A systematic review and meta-analysis. Front Endocrinol (Lausanne) 2022, 13:957136. |
| 8. Wu F, Cui C, Wu J, Wang Y: Can Lipoprotein(a) Predict the Risk of Diabetic Nephropathy in Type 2 Diabetes Mellitus?: A Systematic Review and Meta-Analysis. Horm Metab Res 2025, 57(4):242-251. |
| 9. Ye X, Kong W, Zafar MI, Chen LL: Serum triglycerides as a risk factor for cardiovascular diseases in type 2 diabetes mellitus: a systematic review and meta-analysis of prospective studies. Cardiovasc Diabetol 2019, 18(1):48. |
| 10. Feng X, Yao Y, Wu L, Cheng C, Tang Q, Xu S: Triglyceride-Glucose Index and the Risk of Stroke: A Systematic Review and Dose-Response Meta-Analysis. Horm Metab Res 2022, 54(3):175-186. |
| 11. Min D, Zhao J, Liu M: Atherogenic index of plasma and risk of diabetic nephropathy in type 2 diabetes: A meta‑analysis. Biomolecules & biomedicine 2025. |
| 12. Cao Y, Yan L, Guo N, Yu N, Wang Y, Cao X, Yang S, Lv F: Non-high-density lipoprotein cholesterol and risk of cardiovascular disease in the general population and patients with type 2 diabetes: A systematic review and meta-analysis. Diabetes Research and Clinical Practice 2019, 147:1-8. |
| 13. Khalaji A, Behnoush AH, Khanmohammadi S, Ghanbari Mardasi K, Sharifkashani S, Sahebkar A, Vinciguerra C, Cannavo A: Triglyceride-glucose index and heart failure: a systematic review and meta-analysis. Cardiovasc Diabetol 2023, 22(1):244 |
| 14. Zhou J, Zhu L, Li Y: Association between the triglyceride glucose index and diabetic retinopathy in type 2 diabetes: a meta-analysis. Front Endocrinol (Lausanne) 2023, 14:1302127. |

| **Supplementary Table S14. List of Excluded Studies for primary studies on Lp(a).** |
| --- |
| **A meta-analysis (n = 1)** |
| 1. Paige E, Masconi KL, Tsimikas S, Kronenberg F, Santer P, Weger S, Willeit J, Kiechl S, Willeit P: Lipoprotein(a) and incident type-2 diabetes: results from the prospective Bruneck study and a meta-analysis of published literature. Cardiovasc Diabetol 2017, 16(1):38. |
| **Not prospective cohort study (n = 1)** |
| 1. Robbins DC, Howard BV: Lipoprotein(a) and diabetes. Diabetes Care 1991, 14(4):347-349. |
| **Cross-sectional study (n = 2)** |
| 1. Buchmann N, Scholz M, Lill CM, Burkhardt R, Eckardt R, Norman K, Loeffler M, Bertram L, Thiery J, Steinhagen-Thiessen E et al: Association between lipoprotein(a) level and type 2 diabetes: no evidence for a causal role of lipoprotein(a) and insulin. Acta Diabetol 2017, 54(11):1031-1038. |
| 2. Liu C, Xu MX, He YM, Zhao X, Du XJ, Yang XJ: Lipoprotein (a) is not significantly associated with type 2 diabetes mellitus: cross-sectional study of 1604 cases and 7983 controls. Acta Diabetol 2017, 54(5):443-453. |
| **Randomized controlled trial (n = 1)** |
| 1. Liu W, Zhang G, Nie Z, Guan X, Sun T, Jin X, Li B: Low Concentration of Lipoprotein(a) is an Independent Predictor of Incident Type 2 Diabetes. Horm Metab Res 2024, 56(7):504-508. |
| **Mendelian randomisation study (n = 2)** |
| 1. Ye Z, Haycock PC, Gurdasani D, Pomilla C, Boekholdt SM, Tsimikas S, Khaw KT, Wareham NJ, Sandhu MS, Forouhi NG: The association between circulating lipoprotein(a) and type 2 diabetes: is it causal? Diabetes 2014, 63(1):332-342. |
| 2. Kamstrup PR, Nordestgaard BG: Lipoprotein(a) concentrations, isoform size, and risk of type 2 diabetes: a Mendelian randomisation study. Lancet Diabetes Endocrinol 2013, 1(3):220-227. |
| **Retrospective cohort study (n = 1)** |
| 1. Fu Q, Hu L, Xu Y, Yi Y, Jiang L: High lipoprotein(a) concentrations are associated with lower type 2 diabetes risk in the Chinese Han population: a large retrospective cohort study. Lipids Health Dis 2021, 20(1):76. |
| **Other diseases as the outcome (n = 9)** |
| 1. Lim TS, Yun JS, Cha SA, Song KH, Yoo KD, Ahn YB, Park YM, Ko SH: Elevated lipoprotein(a) levels predict cardiovascular disease in type 2 diabetes mellitus: a 10-year prospective cohort study. Korean J Intern Med 2016, 31(6):1110-1119. |
| 2. Yun JS, Ahn YB, Song KH, Yoo KD, Park YM, Kim HW, Ko SH: Lipoprotein(a) predicts a new onset of chronic kidney disease in people with Type 2 diabetes mellitus. Diabet Med 2016, 33(5):639-643. |
| 3. Buchmann N, Ittermann T, Demuth I, Markus MRP, Völzke H, Dörr M, Friedrich N, Lerch MM, Santos RD, Schipf S et al: Lipoprotein(a) and Metabolic Syndrome. Dtsch Arztebl Int 2022, 119(15):270-276. |
| 4. Chen R, Zhang K, Liu H, Liu L, Li H, Yan Y, Zhou Z, Meng C, Wang X, Wu H et al: Lipoprotein(a) as a Risk Factor for Recurrent Ischemic Stroke in Type 2 Diabetes. Diabetes Metab Syndr Obes 2025, 18:1631-1641. |
| 5. Gholami Chahkand MS, Esmaeilpour Moallem F, Qezelgachi A, Seifouri K, Pesaran Afsharian A, Sheikhzadeh F, Poursalehi A, Fani Sadrabadi FS, Saghab Torbati M, Ramezanzade M et al: Lipoprotein (a) as a predictor of diabetic retinopathy in patients with type 2 diabetes: A systematic review. Diab Vasc Dis Res 2023, 20(6):14791641231197114. |
| 6. Kamstrup PR: Lipoprotein(a) and Cardiovascular Disease. Clin Chem 2021, 67(1):154-166. |
| 7. Li M, Wang Y, Yao Q, Liang Q, Zhang Y, Wang X, Li Q, Qiang W, Yang J, Shi B et al: Association between Lipoprotein(a) and diabetic nephropathy in patients with type 2 diabetes. Front Endocrinol (Lausanne) 2023, 14:1337469. |
| 8. Tu WJ, Liu H, Liu Q, Cao JL, Guo M: Association between serum lipoprotein(a) and diabetic retinopathy in han Chinese patients with type 2 diabetes. Journal of Clinical Endocrinology and Metabolism 2017, 102(7):2525-2532. |
| 9. Wang T: Association between lipoprotein(a) plasma levels and diabetic nephropathy in Han Chinese patients with type 2 diabetes mellitus. PLoS One 2024, 19(5):e0299240 |

| **Supplementary Table S15. List of Excluded Studies for primary studies on Non-HDL-C.** |
| --- |
| **A meta-analysis (n = 1)** |
| 1. Han M, Shen Y, Guo X, Hong C, Ji X, Guo H, Jin Y, Yuan H: Association between non-high-density lipoprotein cholesterol and type 2 diabetes: a systematic review and meta-analysis of cohort studies. Endocr J 2025, 72(1):43-51. |
| **Case-control study (n = 1)** |
| 1. Dali-Sahi M, Kachekouche Y, Dennouni-Medjati N, Nafuye G: Non-HDL cholesterol predictive factor of type 2 diabetes in the city of Tlemcen. Diabetes Metab Syndr 2019, 13(1):518-521. |
| **Other diseases as the outcome (n = 7)** |
| 1. Schulze MB, Shai I, Manson JE, Li T, Rifai N, Jiang R, Hu FB: Joint role of non-HDL cholesterol and glycated haemoglobin in predicting future coronary heart disease events among women with type 2 diabetes. Diabetologia 2004, 47(12):2129-2136. |
| 2. Ding X, Zhou H, Yue Q, Shu Z, Ma X, Li Y, Wu S: Association of trajectories of non-high-density lipoprotein cholesterol concentration with risk of cardiovascular disease: the Kailuan Study. BMJ Open 2023, 13(4):e069807. |
| 3. Lu W, Resnick HE, Jablonski KA, Jones KL, Jain AK, Howard WMJ, Robbins DC, Howard BV: Non-HDL cholesterol as a predictor of cardiovascular disease in type 2 diabetes: The strong heart study. Diabetes Care 2003, 26(1):16-23. |
| 4. Pandeya A, Sharma M, Regmi P, Basukala A, Lamsal M: Pattern of dyslipidemia and evaluation of non-HDL cholesterol as a marker of risk factor for cardiovascular disease in type 2 diabetes mellitus. Nepal Med Coll J 2012, 14(4):278-282. |
| 5. Hong S, Han K, Park JH, Yu SH, Lee CB, Kim DS: Higher Non-High-Density Lipoprotein Cholesterol Was Higher Associated With Cardiovascular Disease Comparing Higher LDL-C in Nine Years Follow Up: Cohort Study. J Lipid Atheroscler 2023, 12(2):164-174. |
| 6. Luo Y, Peng D: Residual Atherosclerotic Cardiovascular Disease Risk: Focus on Non-High-Density Lipoprotein Cholesterol. J Cardiovasc Pharmacol Ther 2023, 28:10742484231189597. |
| 7. Araki A, Iimuro S, Sakurai T, Umegaki H, Iijima K, Nakano H, Oba K, Yokono K, Sone H, Yamada N et al: Non-high-density lipoprotein cholesterol: An important predictor of stroke and diabetes-related mortality in Japanese elderly diabetic patients. Geriatrics and Gerontology International 2012, 12(SUPPL.1):18-28. |

| **Supplementary Table S16. List of Excluded Studies for primary studies on TG/HDL-C.** |
| --- |
| **A meta-analysis (n = 2)** |
| 1. Cheng C, Liu Y, Sun X, Yin Z, Li H, Zhang M, Zhang D, Wang B, Ren Y, Zhao Y et al: Dose–response association between the triglycerides: High-density lipoprotein cholesterol ratio and type 2 diabetes mellitus risk: The rural Chinese cohort study and meta-analysis. Journal of Diabetes 2019, 11(3):183-192. |
| 2. Zhong H, Luo L, Wang X, Xiao Y: Association between triglyceride to HDL cholesterol ratio and a risk of diabetes mellitus: a systematic review and meta-analysis. Laboratory Medicine 2025, 56(1):1-6. |
| **Gestational diabetes (n = 2)** |
| 1. You Y, Hu H, Cao C, Han Y, Tang J, Zhao W: Association between the triglyceride to high-density lipoprotein cholesterol ratio and the risk of gestational diabetes mellitus: a second analysis based on data from a prospective cohort study. Front Endocrinol (Lausanne) 2023, 14:1153072. |
| 2. Ma N, Bai L, Lu Q: First-Trimester Triglyceride-Glucose Index and Triglyceride/High-Density Lipoprotein Cholesterol are Predictors of Gestational Diabetes Mellitus Among the Four Surrogate Biomarkers of Insulin Resistance. Diabetes, Metabolic Syndrome and Obesity 2024, 17:1575-1583. |
| **Retrospective cohort study (n = 5)** |
| 1. Xu G, Song J: The Association Between the Triglyceride to High-Density Lipoprotein Cholesterol Ratio and the Incidence of Type 2 Diabetes Mellitus in the Japanese Population. Metabolic Syndrome and Related Disorders 2024, 22(6):471-478. |
| 2. Qin H, Chen Z, Zhang Y, Wang L, Ouyang P, Cheng L, Zhang Y: Triglyceride to high-density lipoprotein cholesterol ratio is associated with incident diabetes in men: A retrospective study of Chinese individuals. J Diabetes Investig 2020, 11(1):192-198. |
| 3. Sun Y, Wang Z, Huang Z, Hu H, Han Y: The Association Between the Triglyceride-to-High-Density Lipoprotein Cholesterol Ratio and the Risk of Progression to Diabetes From Prediabetes: A 5-year Cohort Study in Chinese Adults. Front Endocrinol (Lausanne) 2022, 13:947157. |
| 4. Huang Z, Zhang X, Sun D, Yu K: Triglyceride to high-density lipoprotein cholesterol ratio is associated with diabetes incidence in non-obese individuals with normoglycemia: a retrospective cohort study based on individuals from East Asia. Front Endocrinol (Lausanne) 2024, 15:1442731. |
| 5. Yang T, Liu Y, Li L, Zheng Y, Wang Y, Su J, Yang R, Luo M, Yu C: Correlation between the triglyceride-to-high-density lipoprotein cholesterol ratio and other unconventional lipid parameters with the risk of prediabetes and Type 2 diabetes in patients with coronary heart disease: a RCSCD-TCM study in China. Cardiovasc Diabetol 2022, 21(1):93. |
| **Other diseases as the outcome (n = 10)** |
| 1. Azarpazhooh MR, Najafi F, Darbandi M, Kiarasi S, Oduyemi T, Spence JD: Triglyceride/High-Density Lipoprotein Cholesterol Ratio: A Clue to Metabolic Syndrome, Insulin Resistance, and Severe Atherosclerosis. Lipids 2021, 56(4):405-412. |
| 2. Lee MY, Hsiao PJ, Huang JC, Hsu WH, Chen SC, Chang JM, Shin SJ: Associations between triglyceride/high-density lipoprotein cholesterol ratio and micro- and macroangiopathies in type 2 diabetes mellitus. Endocrine Practice 2018, 24(7):615-621. |
| 3. Raikou VD, Kyriaki D, Gavriil S: Triglycerides to High-Density Lipoprotein Cholesterol Ratio Predicts Chronic Renal Disease in Patients without Diabetes Mellitus (STELLA Study). J Cardiovasc Dev Dis 2020, 7(3). |
| 4. Lv S, Zhang H, Chen J, Shen Z, Zhu C, Gu Y, Yu X, Zhang D, Wang Y, Ding X et al: The effect of triglycerides to high-density lipoprotein cholesterol ratio on the reduction of renal function: findings from China health and retirement longitudinal study (CHARLS). Lipids Health Dis 2021, 20(1):110. |
| 5. Di Marco M, Scilletta S, Miano N, Capuccio S, Musmeci M, Di Mauro S, Filippello A, Scamporrino A, Bosco G, Di Giacomo Barbagallo F et al: Triglycerides to high density lipoprotein cholesterol ratio (TG/HDL), but not triglycerides and glucose product (TyG) index, is associated with arterial stiffness in prediabetes. Diabetes Research and Clinical Practice 2025, 224. |
| 6. Zhou L, Mai J, Li Y, Guo M, Wu Y, Gao X, Wu Y, Liu X, Zhao L: Triglyceride to high-density lipoprotein cholesterol ratio and risk of atherosclerotic cardiovascular disease in a Chinese population. Nutrition, Metabolism and Cardiovascular Diseases 2020, 30(10):1706-1713. |
| 7. Yang SH, Du Y, Li XL, Zhang Y, Li S, Xu RX, Zhu CG, Guo YL, Wu NQ, Qing P et al: Triglyceride to High-Density Lipoprotein Cholesterol Ratio and Cardiovascular Events in Diabetics With Coronary Artery Disease. American Journal of the Medical Sciences 2017, 354(2):117-124. |
| 8. Quispe R, Martin SS, Jones SR: Triglycerides to high-density lipoprotein-cholesterol ratio, glycemic control and cardiovascular risk in obese patients with type 2 diabetes. Current Opinion in Endocrinology, Diabetes and Obesity 2016, 23(2):150-156. |
| 9. Liao LP, Wu L, Yang Y: The relationship between triglyceride/high-density lipoprotein cholesterol ratio and coronary microvascular disease. BMC Cardiovasc Disord 2023, 23(1):228. |
| 10. Yu Y, Yang ZC, Wang LX: Triglyceride to High-Density Lipoprotein Cholesterol Ratio and Sensorineural Hearing Loss in Community-Dwelling Adults: an NHANES Analysis. Yonsei Medical Journal 2024, 65(12):741-751. |

| **Supplementary Table S17. List of Excluded Studies for primary studies on VAI.** |
| --- |
| **A meta-analysis (n = 2)** |
| 1. Shen F, Guo C, Zhang D, Liu Y, Zhang P: Visceral adiposity index as a predictor of type 2 diabetes mellitus risk: A systematic review and dose–response meta-analysis. Nutrition, Metabolism and Cardiovascular Diseases 2024, 34(4):811-822. |
| 2. Deng R, Chen W, Zhang Z, Zhang J, Wang Y, Sun B, Yin K, Cao J, Fan X, Zhang Y et al: Association Between Visceral Obesity Index and Diabetes: A Systematic Review and Meta-analysis. J Clin Endocrinol Metab 2024, 109(10):2692-2707. |
| **Retrospective cohort study (n = 1)** |
| 1. Shang L, Li R, Zhao Y, Sun H, Tang B, Hou Y: Association Between Chinese Visceral Adiposity Index and Incident Type 2 Diabetes Mellitus in Japanese Adults. Diabetes Metab Syndr Obes 2021, 14:3743-3751. |
| **Cross-sectional study (n = 10)** |
| 1. Liu PJ, Ma F, Lou HP, Chen Y: Visceral adiposity index is associated with pre-diabetes and type 2 diabetes mellitus in Chinese adults aged 20-50. Annals of Nutrition and Metabolism 2016, 68(4):235-243. |
| 2. Qiu LT, Zhang JD, Fan BY, Li L, Sun GX: Association of visceral adiposity index and lipid accumulation products with prediabetes in US adults from NHANES 2007–2020: A cross-sectional study. PLoS ONE 2024, 19(9 September 2024). |
| 3. Huang L, Liao J, Lu C, Yin Y, Ma Y, Wen Y: The non-linear relationship between the visceral adiposity index and the risk of prediabetes and diabetes. Front Endocrinol (Lausanne) 2025, 16:1407873. |
| 4. Zheng D, Zhao C, Ma K, Ruan Z, Zhou H, Wu H, Lu F: Association between visceral adiposity index and risk of diabetes and prediabetes: Results from the NHANES (1999-2018). PLoS One 2024, 19(4):e0299285. |
| 5. Liu Y, Jiang H, Luo L, Gao Z: Relationship between four visceral obesity indices and prediabetes and diabetes: a cross-sectional study in Dalian, China. BMC Endocr Disord 2024, 24(1):191. |
| 6. Zar A, Ali SS: Visceral Adiposity Index: A Simple Tool For Assessing Risk Of Type 2 Diabetes Mellitus. J Ayub Med Coll Abbottabad 2022, 34(2):345-350. |
| 7. Nusrianto R, Tahapary DL, Soewondo P: Visceral adiposity index as a predictor for type 2 diabetes mellitus in Asian population: A systematic review. Diabetes Metab Syndr 2019, 13(2):1231-1235. |
| 8. Lv C, Huo R: Association between visceral adiposity index, lipid accumulation product and type 2 diabetes mellitus in US adults with hypertension: a cross-sectional analysis of NHANES from 2005 to 2018. BMC Endocr Disord 2024, 24(1):216. |
| 9. Gu D, Ding Y, Zhao Y, Qu Q: Visceral Adiposity Index was a useful Predictor of Prediabetes. Exp Clin Endocrinol Diabetes 2018, 126(10):596-603. |
| 10. Zhou H, Li T, Li J, Zhuang X, Yang J: The association between visceral adiposity index and risk of type 2 diabetes mellitus. Sci Rep 2024, 14(1):16634. |
| **Other diseases as the outcome (n = 17)** |
| 1. Zhao X, Sun J, Xin S, Zhang X: Study on the association between visceral adiposity index and diabetic kidney disease in hospitalized patients with type 2 diabetes mellitus in China. Front Endocrinol (Lausanne) 2025, 16:1549954. |
| 2. Tawfik MY, Mohamed SF, Elotla SF: Association of novel visceral obesity indices with 10-year risk of major cardiovascular events in patients with type 2 diabetes mellitus. J Egypt Public Health Assoc 2025, 100(1):12. |
| 3. Motamed N, Khonsari MR, Rabiee B, Ajdarkosh H, Hemasi GR, Sohrabi MR, Maadi M, Zamani F: Discriminatory Ability of Visceral Adiposity Index (VAI) in Diagnosis of Metabolic Syndrome: A Population Based Study. Exp Clin Endocrinol Diabetes 2017, 125(3):202-207. |
| 4. Wen S, Huang X, Huang Z, Zhang X, Dai C, Han F, Zheng W, Wang F, Chen S, Zhang B et al: The association of changes in the Chinese visceral adiposity index and cardiometabolic diseases: a cohort study. Diabetol Metab Syndr 2024, 16(1):228. |
| 5. Liu Q, Sun M, Liu Y, Xu W, Zheng H, Ning N, Huang R, Zhou J, Shao J, Zhou W et al: Chinese Visceral Adiposity Index Trajectory and Stroke in Prediabetes and Diabetes: A Prospective Cohort Study. Diabetes Metab Res Rev 2025, 41(1):e70025. |
| 6. Tang M, Wei XH, Cao H, Zhen Q, Liu F, Wang YF, Fan NG, Peng YD: Association between Chinese visceral adiposity index and metabolic-associated fatty liver disease in Chinese adults with type 2 diabetes mellitus. Front Endocrinol (Lausanne) 2022, 13:935980. |
| 7. Sun M, Liu Q, Liu Y, Ning N, Zhou J, Zhou D, Zheng H, Wu S, Gao J, Ma Y: Baseline and cumulative Chinese visceral adiposity index and diabetic kidney disease: A prospective cohort study. Diabetes, Obesity and Metabolism 2025, 27(4):1920-1931. |
| 8. Ye X, Zhang G, Han C, Wang P, Lu J, Zhang M: The association between Chinese visceral adiposity index and cardiometabolic multimorbidity among Chinese middle-aged and older adults: a national cohort study. Front Endocrinol (Lausanne) 2024, 15:1381949. |
| 9. Kang S, Chen S, Lin Z, Zhang M, Wang J, Liu S, Wang Y, Zhao D, Wu Y, Li Y: Association between Chinese visceral adiposity index and heart failure in diabetic patients. Diabetes Obes Metab 2025. |
| 10. Gao D, Chen X, Liu D, Su D, Cheng Y, Cui Y, Hu W, Li Z, Li G, Zhang X et al: Association Between Chinese Visceral Adiposity Index and Risk of Incident Hypertension Among Older Adults: A Prospective Cohort Study. High Blood Press Cardiovasc Prev 2025. |
| 11. Wu Z, Yu S, Kang X, Liu Y, Xu Z, Li Z, Wang J, Miao X, Liu X, Li X et al: Association of visceral adiposity index with incident nephropathy and retinopathy: a cohort study in the diabetic population. Cardiovasc Diabetol 2022, 21(1):32. |
| 12. Li C, Wang G, Zhang J, Jiang W, Wei S, Wang W, Pang S, Pan C, Sun W: Association between visceral adiposity index and incidence of diabetic kidney disease in adults with diabetes in the United States. Sci Rep 2024, 14(1):17957. |
| 13. Kouli GM, Panagiotakos DB, Kyrou I, Georgousopoulou EN, Chrysohoou C, Tsigos C, Tousoulis D, Pitsavos C: Visceral adiposity index and 10-year cardiovascular disease incidence: The ATTICA study. Nutr Metab Cardiovasc Dis 2017, 27(10):881-889. |
| 14. Chen J, Li YT, Niu Z, He Z, Xie YJ, Hernandez J, Huang W, Wang HHX: Association of Visceral Obesity Indices With Incident Diabetic Retinopathy in Patients With Diabetes: Prospective Cohort Study. JMIR Public Health Surveill 2024, 10:e48120. |
| 15. Li L, Xi L, Wang Q: Association between Chinese visceral adiposity index and risk of new-onset hypertension in middle-aged and older adults with prediabetes: evidence from a large national cohort study. Front Public Health 2025, 13:1509898. |
| 16. Zhang Z, Zhao L, Lu Y, Meng X, Zhou X: Association between Chinese visceral adiposity index and risk of stroke incidence in middle-aged and elderly Chinese population: evidence from a large national cohort study. J Transl Med 2023, 21(1):518. |
| 17. Fan Z, Yang L, Song J, Hu H, Zhu K, Fu X, Luo X: Correlation between Visceral Adiposity Index (VAI) and Congestive Heart Failure (CHF) in patients with hypertension: a study based on NHANES 2001–2018. BMC Cardiovascular Disorders 2025, 25(1). |

| **Supplementary Table S18. List of Excluded Studies for primary studies on LAP.** |
| --- |
| **A meta-analysis (n = 1)** |
| 1. Khanmohammadi S, Tavolinejad H, Aminorroaya A, Rezaie Y, Ashraf H, Vasheghani-Farahani A. Association of lipid accumulation product with type 2 diabetes mellitus, hypertension, and mortality: a systematic review and meta-analysis. J Diabetes Metab Disord. 2022 Aug 30;21(2):1943-1973. doi: 10.1007/s40200-022-01114-z. PMID: 36404835; PMCID: PMC9672205. |
| **Retrospective cohort study (n = 3)** |
| 1. Liu T, Lu W, Zhao X, Yao T, Song B, Fan H, Gao G, Liu C. Relationship between lipid accumulation product and new-onset diabetes in the Japanese population: a retrospective cohort study. Front Endocrinol (Lausanne). 2023 May 17;14:1181941. doi: 10.3389/fendo.2023.1181941. PMID: 37265697; PMCID: PMC10230034. |
| 1. Ma E, Ohira T, Fukasawa M, Shirafuji A, Matsuzaki K, Hosoya M, Yasumura S, Shimabukuro M. Predictive value of obesity-related indices for incident type 2 diabetes mellitus: a longitudinal study of the Fukushima Health Database 2015-2021. Diabetol Metab Syndr. 2025 Jun 21;17(1):236. doi: 10.1186/s13098-025-01795-5. PMID: 40544266; PMCID: PMC12181894. |
| 1. Yang T, Zhao B, Pei D. Evaluation of the Association between Obesity Markers and Type 2 Diabetes: A Cohort Study Based on a Physical Examination Population. J Diabetes Res. 2021 Dec 28;2021:6503339. doi: 10.1155/2021/6503339. PMID: 34993251; PMCID: PMC8727144. |
| **Cross-sectional study (n = 4)** |
| 1. Lv C, Huo R. Association between visceral adiposity index, lipid accumulation product and type 2 diabetes mellitus in US adults with hypertension: a cross-sectional analysis of NHANES from 2005 to 2018. BMC Endocr Disord. 2024 Oct 15;24(1):216. doi: 10.1186/s12902-024-01750-x. PMID: 39407231; PMCID: PMC11476220. |
| 2. Lin CY, Li JB, Wu F, Wang JJ, An HH, Qiu HN, Xia LF, Li YS, Zhai YJ, Li CJ, Lin JN. Comparison of lipid accumulation product and visceral adiposity index with traditional obesity indices in early-onset type 2 diabetes prediction: a cross-sectional study. Diabetol Metab Syndr. 2023 May 27;15(1):111. doi: 10.1186/s13098-023-01056-3. PMID: 37237396; PMCID: PMC10223887. |
| 3. Kavaric N, Klisic A, Ninic A. Are visceral adiposity index and lipid accumulation product reliable indices for metabolic disturbances in patients with type 2 diabetes mellitus? J Clin Lab Anal. 2018 Mar;32(3):e22283. doi: 10.1002/jcla.22283. Epub 2017 Jun 20. PMID: 28632304; PMCID: PMC6816927. |
| 4. Sun K, Lin D, Feng Q, Li F, Qi Y, Feng W, Yang C, Yan L, Ren M, Liu D. Assessment of adiposity distribution and its association with diabetes and insulin resistance: a population-based study. Diabetol Metab Syndr. 2019 Jun 27;11:51. doi: 10.1186/s13098-019-0450-x. PMID: 31297161; PMCID: PMC6598265. |
| **Other diseases as the outcome (n = 10)** |
| 1. Bardini G, Giannini S, Romano D, Rotella CM, Mannucci E. Lipid accumulation product and 25-OH-vitamin D deficiency in type 2 diabetes. Rev Diabet Stud. 2013 Winter;10(4):243-51. doi: 10.1900/RDS.2013.10.243. Epub 2014 Feb 10. PMID: 24841878; PMCID: PMC4160011. |
| 2. Yu X, Pu X, Xi Y, Li X, Li H, Zheng D. Association between the lipid accumulation product and chronic kidney disease among adults in the United States. Sci Rep. 2024 Sep 13;14(1):21423. doi: 10.1038/s41598-024-71894-2. PMID: 39271739; PMCID: PMC11399144. |
| 3. Tang M, Yao S, Cao H, Wei X, Zhen Q, Tan Y, Liu F, Wang Y, Peng Y, Fan N. Interrelation between the lipid accumulation product index and diabetic kidney disease in patients with type 2 diabetes mellitus. Front Endocrinol (Lausanne). 2023 Aug 14;14:1224889. doi: 10.3389/fendo.2023.1224889. PMID: 37645414; PMCID: PMC10461558. |
| 4. Zhu Z, Miao X, Wang H, Deng Y, Shi H, Li S. Association between lipid accumulation product and stroke incidence: A national cohort study based on CHARLS. J Stroke Cerebrovasc Dis. 2025 Oct;34(10):108411. doi: 10.1016/j.jstrokecerebrovasdis.2025.108411. Epub 2025 Aug 5. PMID: 40759191. |
| 5. Qi L, Kang N, Li Y, Zhao H, Chen S. The Predictive Value of Visceral Adiposity Index and Lipid Accumulation Index for Microalbuminuria in Newly Diagnosed Type 2 Diabetes Patients. Diabetes Metab Syndr Obes. 2021 Mar 11;14:1107-1115. doi: 10.2147/DMSO.S302761. PMID: 33737822; PMCID: PMC7961207. |
| 6. H Wacher N, Gómez-Díaz RA, Valdez-González AL, Duque-López X, Morán-Villota S, Mondragón-González R, Cruz-López M, Borja-Aburto VH. Factores predictivos de MASLD en diabetes tipo 2: estudio de seguimiento de un año [Predictive factors for MASLD in type 2 diabetes: 1 year follow-up study]. Rev Med Inst Mex Seguro Soc. 2024 Sep 5;62(suppl 2):1-12. Spanish. doi: 10.5281/zenodo.10814344. PMID: 39602549; PMCID: PMC12646365. |
| 7. Wan H, Wang Y, Xiang Q, Fang S, Chen Y, Chen C, Zhang W, Zhang H, Xia F, Wang N, Lu Y. Associations between abdominal obesity indices and diabetic complications: Chinese visceral adiposity index and neck circumference. Cardiovasc Diabetol. 2020 Jul 31;19(1):118. doi: 10.1186/s12933-020-01095-4. PMID: 32736628; PMCID: PMC7395356. |
| 8. Mao J, Gan S, Zhou Q, Yu F, Zhou H, Lu H, Jin J, Liu Q, Deng Z. Positive correlation between lipid accumulation product index and arterial stiffness in Chinese patients with type 2 diabetes. Front Endocrinol (Lausanne). 2023 Nov 23;14:1277162. doi: 10.3389/fendo.2023.1277162. PMID: 38075069; PMCID: PMC10702222. |
| 9. Yu ZW, Li X, Wang Y, Fu YH, Gao XY. Association Between Lipid Accumulation Product and Mild Cognitive Impairment in Patients with Type 2 Diabetes. J Alzheimers Dis. 2020;77(1):367-374. doi: 10.3233/JAD-200332. PMID: 32804130. |
| 10. Dong L, Lin M, Wang W, Ma D, Chen Y, Su W, Chen Z, Wang S, Li X, Li Z, Liu C. Lipid accumulation product (LAP) was independently associatedwith obstructive sleep apnea in patients with type 2 diabetes mellitus. BMC Endocr Disord. 2020 Dec 9;20(1):179. doi: 10.1186/s12902-020-00661-x. PMID: 33298050; PMCID: PMC7727244. |

| **Supplementary Table S19. List of Excluded Studies for primary studies on TyG.** |
| --- |
| **A meta-analysis (n = 2)** |
| 1. da Silva A, Caldas APS, Rocha DMUP, Bressan J: Triglyceride-glucose index predicts independently type 2 diabetes mellitus risk: A systematic review and meta-analysis of cohort studies. Primary Care Diabetes 2020, 14(6):584-593. |
| 2. Pranata R, Huang I, Irvan, Lim MA, Vania R: The association between triglyceride-glucose index and the incidence of type 2 diabetes mellitus—a systematic review and dose–response meta-analysis of cohort studies. Endocrine 2021, 74(2):254-262. |
| **No data available (n = 1)** |
| 1. D'Elia L, Rendina D, Iacone R, Strazzullo P, Galletti F: Triglyceride-Glucose Index and New-Onset Type 2 Diabetes Mellitus in Middle-Aged Men. Metabolites 2025, 15(8). |
| **Interventional study (n = 2)** |
| 1. Zhou J, Kong W, Chen J, Xie P, Sun W, Liu Y, Hao L, Wang Y, Ni H: [Correlation between triglyceride glucose product index and food intake and type 2 diabetes]. Wei Sheng Yan Jiu 2024, 53(5):755-762. |
| 2. Liu D, Liu Z, Wu Y, Hong Y, Fang J, Lu Y, Xu G, Kang P, Liu T, Chen LH: Dietary patterns related to triglyceride glucose index and risk of type 2 diabetes: a large-scale cohort study. Front Nutr 2024, 11:1510926. |
| **Cross-sectional study (n = 1)** |
| 1. Zhang L, Zeng L: Non-linear association of triglyceride-glucose index with prevalence of prediabetes and diabetes: a cross-sectional study. Front Endocrinol (Lausanne) 2023, 14:1295641. |
| **Retrospective cohort study (n = 8)** |
| 1. Zhang J, Fang X, Song Z, Guo XK, Lin DM, Jiang FN, Lin L, Cai ZH: Positive association of triglyceride glucose index and gestational diabetes mellitus: a retrospective cohort study. Front Endocrinol (Lausanne) 2024, 15:1475212. |
| 2. Liu EQ, Weng YP, Zhou AM, Zeng CL: Association between Triglyceride-Glucose Index and Type 2 Diabetes Mellitus in the Japanese Population: A Secondary Analysis of a Retrospective Cohort Study. Biomed Res Int 2020, 2020:2947067. |
| 3. Cao C, Hu H, Xiao P, Zan Y, Chang X, Han Y, Zhang X, Wang Y: Nonlinear relationship between triglyceride-glucose index and the risk of prediabetes and diabetes: a secondary retrospective cohort study. Front Endocrinol (Lausanne) 2024, 15:1416634. |
| 4. Kim JA, Kim J, Roh E, Hong SH, Lee YB, Baik SH, Choi KM, Noh E, Hwang SY, Cho GJ et al: Triglyceride and glucose index and the risk of gestational diabetes mellitus: A nationwide population-based cohort study. Diabetes Res Clin Pract 2021, 171:108533. |
| 5. Luo H, Yang Q, Xu H, Wu S, Wang W, Zhou R, Yang Y, Yu Q: Association between triglyceride-glucose index and the risk of type 2 diabetes mellitus. Arch Endocrinol Metab 2025, 69(2):e230493. |
| 6. Low S, Khoo KCJ, Irwan B, Sum CF, Subramaniam T, Lim SC, Wong TKM: The role of triglyceride glucose index in development of Type 2 diabetes mellitus. Diabetes Research and Clinical Practice 2018, 143:43-49. |
| 7. Chamroonkiadtikun P, Ananchaisarp T, Wanichanon W: The triglyceride-glucose index, a predictor of type 2 diabetes development: A retrospective cohort study. Primary Care Diabetes 2020, 14(2):161-167. |
| 8. Bai X, Zhu Q, Wang W, Kan S, Hu S, Hao R, Wang S, Shi Z: Second-trimester triglyceride-glucose index to predict adverse outcomes in women with gestational diabetes mellitus: A retrospective multicenter cohort study. J Diabetes Investig 2024, 15(10):1489-1499. |
| **a cross-sectional retrospective analysis (n = 2)** |
| 1. Sun Y, Gu Y, Zhou Y, Liu A, Lin X, Wang X, Du Y, Lv X, Zhou J, Li Z et al: Nonlinear association between the triglyceride-glucose index and diabetes mellitus in overweight and obese individuals: a cross-sectional retrospective analysis. Diabetol Metab Syndr 2024, 16(1):193. |
| 2. Zeng Y, Yin L, Yin X, Zhao D: Association of triglyceride-glucose index levels with gestational diabetes mellitus in the US pregnant women: a cross-sectional study. Front Endocrinol (Lausanne) 2023, 14:1241372. |
| **Other diseases as the outcome (n = 28)** |
| 1. Yu P, Pu J, Yuan Q, Huang L, Tao L, Peng Z: The prognostic value of triglyceride-glucose index to adverse renal outcomes in patients with type 2 diabetes mellitus: results from the cohort study of ACCORD. Diabetol Metab Syndr 2024, 16(1):201. |
| 2. Siddiqui K, Nawaz SS, George TP, David SK, Alfadda AA, Rafiullah M: Association of triglyceride-glucose index with diabetic kidney disease in patients with type 2 diabetes. J Diabetes Metab Disord 2025, 24(2):171. |
| 3. Shi Y, Yu C: U shape association between triglyceride glucose index and congestive heart failure in patients with diabetes and prediabetes. Nutr Metab (Lond) 2024, 21(1):42. |
| 4. Li C, Qi Q, Li W, Zhang X, Li L, Deng J, Han Q, Wu S, Li K: Triglyceride-Glucose Index Predicts Major Adverse Cardiovascular and Cerebrovascular Events in Non-Diabetic Individuals. Balkan Med J 2025, 42(4):339-346. |
| 5. Zhao M, Xiao M, Tan Q, Lu F: Triglyceride glucose index as a predictor of mortality in middle-aged and elderly patients with type 2 diabetes in the US. Sci Rep 2023, 13(1):16478. |
| 6. Tai S, Fu L, Zhang N, Yang R, Zhou Y, Xing Z, Wang Y, Zhou S: Association of the cumulative triglyceride-glucose index with major adverse cardiovascular events in patients with type 2 diabetes. Cardiovasc Diabetol 2022, 21(1):161. |
| 7. Fritz J, Bjørge T, Nagel G, Manjer J, Engeland A, Häggström C, Concin H, Teleka S, Tretli S, Gylling B et al: The triglyceride-glucose index as a measure of insulin resistance and risk of obesity-related cancers. Int J Epidemiol 2020, 49(1):193-204. |
| 8. Liao Q, Chen Y, Peng Q, Li C: Relationship between triglyceride-glucose index and gallstones risk: a population-based study. Front Endocrinol (Lausanne) 2024, 15:1420999. |
| 9. Huang Q, Nan W, He B, Xing Z, Peng Z: Association of baseline and trajectory of triglyceride-glucose index with the incidence of cardiovascular autonomic neuropathy in type 2 diabetes mellitus. Cardiovasc Diabetol 2025, 24(1):66. |
| 10. Zhao Y, Lv X, Chen C, Li K, Wang Y, Liu J: The association between triglyceride-glucose index and hyperferritinemia in patients with type 2 diabetes mellitus. Hormones 2023, 22(3):403-412. |
| 11. Zhai YJ, Lin CY, Li JB, Qiu HN, Wu F, Wang YL, Lin JN: Mediating role of triglyceride-glucose index and its derivatives in the relationship between central obesity and Hashimoto thyroiditis in type 2 diabetes. Lipids Health Dis 2024, 23(1):411. |
| 12. Pan Y, Zhong S, Zhou K, Tian Z, Chen F, Liu Z, Geng Z, Li S, Huang R, Wang H et al: Association between Diabetes Complications and the Triglyceride-Glucose Index in Hospitalized Patients with Type 2 Diabetes. Journal of Diabetes Research 2021, 2021. |
| 13. Lopez-Jaramillo P, Gomez-Arbelaez D, Martinez-Bello D, Abat MEM, Alhabib KF, Avezum Á, Barbarash O, Chifamba J, Diaz ML, Gulec S et al: Association of the triglyceride glucose index as a measure of insulin resistance with mortality and cardiovascular disease in populations from five continents (PURE study): a prospective cohort study. Lancet Healthy Longev 2023, 4(1):e23-e33. |
| 14. Pan Y, Zhao M, Song T, Tang J, Kuang M, Liu H, Zhong S: Role of Triglyceride-Glucose Index in Type 2 Diabetes Mellitus and Its Complications. Diabetes Metab Syndr Obes 2024, 17:3325-3333. |
| 15. Wu X, Qiu W, Yang H, Chen YJ, Liu J, Zhao G: Associations of the triglyceride-glucose index and atherogenic index of plasma with the severity of new-onset coronary artery disease in different glucose metabolic states. Cardiovasc Diabetol 2024, 23(1):76. |
| 16. Yan Y, Zhou L, La R, Jiang M, Jiang D, Huang L, Xu W, Wu Q: The association between triglyceride glucose index and arthritis: a population-based study. Lipids Health Dis 2023, 22(1):132. |
| 17. Li J, Dong Z, Wu H, Liu Y, Chen Y, Li S, Zhang Y, Qi X, Wei L: The triglyceride-glucose index is associated with atherosclerosis in patients with symptomatic coronary artery disease, regardless of diabetes mellitus and hyperlipidaemia. Cardiovasc Diabetol 2023, 22(1):224. |
| 18. Wang X, Xu W, Song Q, Zhao Z, Meng X, Xia C, Xie Y, Yang C, Jin P, Wang F: Association between the triglyceride–glucose index and severity of coronary artery disease. Cardiovascular Diabetology 2022, 21(1). |
| 19. Wang L, Cong HL, Zhang JX, Hu YC, Wei A, Zhang YY, Yang H, Ren LB, Qi W, Li WY et al: Triglyceride-glucose index predicts adverse cardiovascular events in patients with diabetes and acute coronary syndrome. Cardiovasc Diabetol 2020, 19(1):80. |
| 20. Huang J, Rozi R, Ma J, Fu B, Lu Z, Liu J, Ding Y: Association between higher triglyceride glucose index and increased risk of osteoarthritis: data from NHANES 2015-2020. BMC Public Health 2024, 24(1):758. |
| 21. Si Y, Fan W, Shan W, Zhang Y, Liu J, Han C, Sun L: Association between triglyceride glucose index and coronary artery disease with type 2 diabetes mellitus in middle-aged and elderly people. Medicine (Baltimore) 2021, 100(9):e25025. |
| 22. Guo D, Wu Z, Xue F, Chen S, Ran X, Zhang C, Yang J: Association between the triglyceride-glucose index and impaired cardiovascular fitness in non-diabetic young population. Cardiovasc Diabetol 2024, 23(1):39. |
| 23. Yao Y, Wang B, Geng T, Chen J, Chen W, Li L: The association between TyG and all-cause/non-cardiovascular mortality in general patients with type 2 diabetes mellitus is modified by age: results from the cohort study of NHANES 1999–2018. Cardiovascular Diabetology 2024, 23(1). |
| 24. Forbes M, Mohebbi M, Woods RL, Lotfaliany M, Reynolds CF, 3rd, O'Neil A, McNeil JJ, Berk M: Triglyceride-glucose index and its association with depressive symptoms in older adults: a longitudinal analysis. J Affect Disord 2025, 384:80-85. |
| 25. Wang H, Chen G, Sun D, Ma Y: The threshold effect of triglyceride glucose index on diabetic kidney disease risk in patients with type 2 diabetes: unveiling a non-linear association. Front Endocrinol (Lausanne) 2024, 15:1411486. |
| 26. Zhan J, Wei Q, Guo W, Liu Z, Chen S, Huang Q, Liang S, Cai D: Evaluating the triglyceride glucose index as a predictive biomarker for osteoporosis in patients with type 2 diabetes. Front Endocrinol (Lausanne) 2025, 16:1534232. |
| 27. Zhang Q, Xiao S, Jiao X, Shen Y: The triglyceride-glucose index is a predictor for cardiovascular and all-cause mortality in CVD patients with diabetes or pre-diabetes: evidence from NHANES 2001–2018. Cardiovascular Diabetology 2023, 22(1). |
| 28. Mo Z, Cao C, Han Y, Hu H, He Y, Zuo X: Relationships between triglyceride-glucose index and incident gestational diabetes mellitus: a prospective cohort study of a Korean population using publicly available data. Front Public Health 2024, 12:1294588. |

| **Supplementary Table S20. List of Excluded Studies for primary studies on HTW.** |
| --- |
| **A meta-analysis (n = 2)** |
| 1. Ren Y, Luo X, Wang C, Yin L, Pang C, Feng T, Wang B, Zhang L, Li L, Yang X et al: Prevalence of hypertriglyceridemic waist and association with risk of type 2 diabetes mellitus: A meta-analysis. Diabetes/Metabolism Research and Reviews 2016, 32(4):405-412. |
| 2. Ma CM, Liu XL, Lu N, Wang R, Lu Q, Yin FZ: Hypertriglyceridemic waist phenotype and abnormal glucose metabolism: a system review and meta-analysis. Endocrine 2019, 64(3):469-485. |
| **Gestational diabetes (n = 1)** |
| 1. Jia XJ, Wang JX, Bai LW, Hua TS, Han ZH, Lu Q: The relationship between hypertriglyceridemic waist circumference phenotype and gestational diabetes mellitus. Gynecological Endocrinology 2021, 37(4):328-331. |
| **Retrospective cohort study (n = 2)** |
| 1. Han KJ, Lee SY, Kim NH, Chae HB, Lee TH, Jang CM, Yoo KM, Park HJ, Lee MK, Jeon WS et al: Increased risk of diabetes development in subjects with the hypertriglyceridemic waist phenotype: a 4-year longitudinal study. Endocrinol Metab (Seoul) 2014, 29(4):514-521. |
| 2. Chen D, Liang Z, Sun H, Lu C, Chen W, Wang HHX, Guo VY: Association between hypertriglyceridemic–waist phenotype and risk of type 2 diabetes mellitus in middle-aged and older chinese population: A longitudinal cohort study. International Journal of Environmental Research and Public Health 2021, 18(18). |
| **Cross-sectional study (n = 9)** |
| 1. Miñambres I, Sánchez-Hernández J, Cuixart G, Sánchez-Pinto A, Sarroca J, Pérez A: Characterization of the hypertriglyceridemic waist phenotype in patients with type 2 diabetes mellitus in Spain: An epidemiological study. Revista Clinica Espanola 2021, 221(10):576-581. |
| 2. Amini M, Esmaillzadeh A, Sadeghi M, Mehvarifar N, Amini M, Zare M: The association of hypertriglyceridemic waist phenotype with type 2 diabetes mellitus among individuals with first relative history of diabetes. J Res Med Sci 2011, 16(2):156-164. |
| 3. Rumbea DA, Mera RM, Arias EE, Arriaga K, Del Brutto OH: Prevalence of hypertriglyceridemic-waist phenotype and its association with type 2 diabetes mellitus among middle-aged and older adults of Amerindian ancestry. J Prim Care Community Health 2025, 16:21501319251362044. |
| 4. Carlsson AC, Risérus U, Ärnlöv J: Hypertriglyceridemic waist phenotype is associated with decreased insulin sensitivity and incident diabetes in elderly men. Obesity 2014, 22(2):526-529. |
| 5. Miñambres I, Sánchez-Hernández J, Cuixart G, Sánchez-Pinto A, Sarroca J, Pérez A: Characterization of the hypertriglyceridemic waist phenotype in patients with type2 diabetes mellitus in Spain: An epidemiological study. Rev Clin Esp 2020. |
| 6. Díaz-Santana MV, Suárez Pérez EL, Ortiz Martínez AP, Guzmán Serrano M, Pérez Cardona CM: Association Between the Hypertriglyceridemic Waist Phenotype, Prediabetes, and Diabetes Mellitus Among Adults in Puerto Rico. J Immigr Minor Health 2016, 18(1):102-109. |
| 7. He S, Zheng Y, Shu Y, He J, Wang Y, Chen X: Hypertriglyceridemic waist might be an alternative to metabolic syndrome for predicting future diabetes mellitus. PLoS One 2013, 8(9):e73292. |
| 8. Du T, Sun X, Huo R, Yu X: Visceral adiposity index, hypertriglyceridemic waist and risk of diabetes: The China Health and Nutrition Survey 2009. International Journal of Obesity 2014, 38(6):840-847. |
| 9. Chen S, Guo X, Yu S, Sun G, Li Z, Sun Y: Association between the Hypertriglyceridemic Waist Phenotype, Prediabetes, and Diabetes Mellitus in Rural Chinese Population: A Cross-Sectional Study. Int J Environ Res Public Health 2016, 13(4):368. |
| **Other diseases as the outcome (n = 5)** |
| 1. Wang A, Li Z, Zhou Y, Wang C, Luo Y, Liu X, Guo X, Wu S, Zhao X: Hypertriglyceridemic waist phenotype and risk of cardiovascular diseases in China: Results from the Kailuan Study. International Journal of Cardiology 2014, 174(1):106-109. |
| 2. Ma CM, Wang R, Liu XL, Lu N, Lu Q, Yin FZ: The Relationship between Hypertriglyceridemic Waist Phenotype and Early Diabetic Nephropathy in Type 2 Diabetes. Cardiorenal Med 2017, 7(4):295-300. |
| 3. Wang W, Shen C, Zhao H, Tang W, Yang S, Li J, Ren Z, Zhao Y: A prospective study of the hypertriglyceridemic waist phenotype and risk of incident ischemic stroke in a Chinese rural population. Acta Neurologica Scandinavica 2018, 138(2):156-162. |
| 4. Millán Núñez-Cortés J, Mantilla Morató T, Toro R, Millán Pérez J, Mangas Rojas A: [METABOLIC SYNDROME IN PATIENTS WITH CLINICAL PHENOTYPE "HYPERTRIGLYCERIDEMIC WAIST"]. Nutr Hosp 2015, 32(3):1145-1152. |
| 5. Li Y, Zhou C, Shao X, Liu X, Guo J, Zhang Y, Wang H, Wang X, Li B, Deng K et al: Hypertriglyceridemic waist phenotype and chronic kidney disease in a Chinese population aged 40 years and older. PLoS One 2014, 9(3):e92322. |

| **Supplementary Table S21. List of Excluded Studies for primary studies on AIP.** |
| --- |
| **Gestational diabetes (n = 1)** |
| 1. Zhang J, Suo Y, Wang L, Liu D, Jia Y, Fu Y, Fan W, Jiang Y: Association between atherogenic index of plasma and gestational diabetes mellitus: a prospective cohort study based on the Korean population. Cardiovasc Diabetol 2024, 23(1):237. |
| **Cross-sectional study (n = 4)** |
| 1. Sun Y, Li F, Zhou Y, Liu A, Lin X, Zou Z, Lv X, Zhou J, Li Z, Wu X et al: Nonlinear association between atherogenic index of plasma and type 2 diabetes mellitus in overweight and obesity patients: evidence from Chinese medical examination data. Cardiovasc Diabetol 2024, 23(1):226. |
| 2. Jiang L, Li L, Xu Z, Tang Y, Zhai Y, Fu X, Liu D, Wu Q: Non-linear associations of atherogenic index of plasma with prediabetes and type 2 diabetes mellitus among Chinese adults aged 45 years and above: a cross-sectional study from CHARLS. Front Endocrinol (Lausanne) 2024, 15:1360874. |
| 3. Shi Y, Wen M: Sex-specific differences in the effect of the atherogenic index of plasma on prediabetes and diabetes in the NHANES 2011–2018 population. Cardiovascular Diabetology 2023, 22(1). |
| 4. Yin B, Wu Z, Xia Y, Xiao S, Chen L, Li Y: Non-linear association of atherogenic index of plasma with insulin resistance and type 2 diabetes: a cross-sectional study. Cardiovasc Diabetol 2023, 22(1):157. |
| **Retrospective cohort study (n = 6)** |
| 1. Cao J, Su Z, Yang J, Zhang B, Jiang R, Lu W, Huang Z, Xie Z: The atherogenic index of plasma is associated with an increased risk of diabetes in non-obese adults: a cohort study. Front Endocrinol (Lausanne) 2024, 15:1477419. |
| 2. Liu D, Lou M, Tang Y, Li C, He H: A J-shaped association between the atherogenic index of plasma and diabetes risk in a Japanese population: a large-scale retrospective cohort study. BMC Endocr Disord 2025, 25(1):141. |
| 3. Sun Y, Lin X, Zou Z, Zhao C, Liu A, Zhou J, Li Z, Wu X, Dou S, Zhu J et al: Baseline atherogenic index of plasma and its trajectory predict onset of type 2 diabetes in a health screened adult population: a large longitudinal study. Cardiovasc Diabetol 2025, 24(1):57. |
| 4. Cai X, Xu M, Chen J, Mao Y, Hu J, Li L, Pan J, Jin M, Chen L: Association Between the Trajectories of the Atherogenic Index of Plasma and Prediabetes Progression to Diabetes: A Retrospective Cohort Study. Diabetes, Metabolic Syndrome and Obesity 2024, 17:4689-4699. |
| 5. Zhou Q, Wu Y, Li M: Association between the atherogenic index of plasma and long-term risk of type 2 diabetes: a 12-year cohort study based on the Japanese population. Cardiovasc Diabetol 2025, 24(1):50. |
| 6. Yang H, Kuang M, Yang R, Xie G, Sheng G, Zou Y: Evaluation of the role of atherogenic index of plasma in the reversion from Prediabetes to normoglycemia or progression to Diabetes: a multi-center retrospective cohort study. Cardiovasc Diabetol 2024, 23(1):17. |
| **Other diseases as the outcome (n = 9)** |
| 1. Zhang J, Liu C, Peng Y, Fang Q, Wei X, Zhang C, Sun L, Hu Z, Hong J, Gu W et al: Impact of baseline and trajectory of the atherogenic index of plasma on incident diabetic kidney disease and retinopathy in participants with type 2 diabetes: a longitudinal cohort study. Lipids Health Dis 2024, 23(1):11. |
| 2. Yuan Y, Shi J, Sun W, Kong X: The positive association between the atherogenic index of plasma and the risk of new-onset hypertension: a nationwide cohort study in China. Clin Exp Hypertens 2024, 46(1):2303999. |
| 3. Rokicka D, Hudzik B, Wróbel M, Stołtny T, Stołtny D, Nowowiejska-Wiewióra A, Rokicka S, Gąsior M, Strojek K: Prognostic value of novel atherogenic indices in patients with acute myocardial infarction with and without type 2 diabetes. J Diabetes Complications 2024, 38(10):108850. |
| 4. Wang M, Liu S, Liu L, Wen X, Liao Y, Liu H, Wu S, Wu Y: Association between the cumulative exposure to atherogenic index of plasma and risk of cardiometabolic diseases: a prospective cohort study. Endocrine 2025, 89(1):67-78. |
| 5. Lin J, Li Y, Li H: Association between atherogenic index of plasma and dementia: A longitudinal observational study. J Alzheimers Dis 2025, 106(1):197-205. |
| 6. Li Z, Huang Q, Sun L, Bao T, Dai Z: Atherogenic Index in Type 2 Diabetes and Its Relationship with Chronic Microvascular Complications. Int J Endocrinol 2018, 2018:1765835. |
| 7. Fu L, Zhou Y, Sun J, Zhu Z, Xing Z, Zhou S, Wang Y, Tai S: Atherogenic index of plasma is associated with major adverse cardiovascular events in patients with type 2 diabetes mellitus. Cardiovasc Diabetol 2021, 20(1):201. |
| 8. Qin M, Chen B: Association of atherogenic index of plasma with cardiovascular disease mortality and all-cause mortality in the general US adult population: results from NHANES 2005–2018. Cardiovascular Diabetology 2024, 23(1). |
| 9. Qu L, Fang S, Lan Z, Xu S, Jiang J, Pan Y, Xu Y, Zhu X, Jin J: Association between atherogenic index of plasma and new-onset stroke in individuals with different glucose metabolism status: insights from CHARLS. Cardiovasc Diabetol 2024, 23(1):215. |

| **Supplementary Table S22. List of Excluded Studies for primary studies on FAs.** |
| --- |
| **A meta-analysis (n = 2)** |
| 1. Huang L, Lin JS, Aris IM, Yang G, Chen WQ, Li LJ: Circulating Saturated Fatty Acids and Incident Type 2 Diabetes: A Systematic Review and Meta-Analysis. Nutrients 2019, 11(5). |
| 2. Chen G, Li Y, Zeng F, Deng G, Liang J, Wang J, Su Y, Chen Y, Mao L, Liu Z et al: Biomarkers of fatty acids and risk of type 2 diabetes: a systematic review and meta-analysis of prospective cohort studies. Crit Rev Food Sci Nutr 2021, 61(16):2705-2718. |
| **A review (n = 1)** |
| 1. Shetty SS, Kumari S: Fatty acids and their role in type-2 diabetes (Review). Exp Ther Med 2021, 22(1):706. |
| **Not prospective cohort study (n = 3)** |
| 1. Reaven GM, Chen YD: Role of abnormal free fatty acid metabolism in the development of non-insulin-dependent diabetes mellitus. Am J Med 1988, 85(5a):106-112. |
| 2. Nunes EA, Rafacho A: Implications of palmitoleic acid (Palmitoleate) on glucose homeostasis, insulin resistance and diabetes. Current Drug Targets 2017, 18(6):619-628. |
| 3. Wyne KL: Free fatty acids and type 2 diabetes mellitus. American Journal of Medicine 2003, 115(8 SUPPL. 1):29-36. |
| **Randomized controlled trial (n = 1)** |
| 1. Holmes D: Diabetes: saturated fatty acids--not all bad news. Nat Rev Endocrinol 2014, 10(11):639. |
| **Other diseases as the outcome (n = 2)** |
| 1. Zhang MH, Cao YX, Wu LG, Guo N, Hou BJ, Sun LJ, Guo YL, Wu NQ, Dong Q, Li JJ: Association of plasma free fatty acids levels with the presence and severity of coronary and carotid atherosclerotic plaque in patients with type 2 diabetes mellitus. BMC Endocr Disord 2020, 20(1):156. |
| 2. Chitsazan M, Chitsazan M: The role of free fatty acids as a prognostic biomarker in coronary artery disease patients with type 2 diabetes. Eur J Prev Cardiol 2023, 30(8):728-729. |
| **Interventional study (n = 18)** |
| 1. Virtanen JK, Mursu J, Voutilainen S, Uusitupa M, Tuomainen TP: Serum omega-3 polyunsaturated fatty acids and risk of incident type 2 diabetes in men: The kuopio ischemic heart disease risk factor study. Diabetes Care 2014, 37(1):189-196. |
| 2. Østerud B, Elvevoll EO: Dietary omega-3 fatty acids and risk of type 2 diabetes: Lack of antioxidants? American Journal of Clinical Nutrition 2011, 94(2):617-618. |
| 3. Mozaffarian D: Saturated fatty acids and type 2 diabetes: more evidence to re-invent dietary guidelines. Lancet Diabetes Endocrinol 2014, 2(10):770-772. |
| 4. Kaushik M, Mozaffarian D, Spiegelman D, Manson JE, Willett WC, Hu FB: Long-chain omega-3 fatty acids, fish intake, and the risk of type 2 diabetes mellitus. Am J Clin Nutr 2009, 90(3):613-620. |
| 5. Khumalo S, Duma Z, Bekker L, Nkoana K, Pheeha SM: Type 2 Diabetes Mellitus in Low- and Middle-Income Countries: The Significant Impact of Short-Chain Fatty Acids and Their Quantification. Diagnostics (Basel) 2024, 14(15). |
| 6. Jafari T, Fallah AA, Azadbakht L: Role of dietary n-3 polyunsaturated fatty acids in type 2 diabetes: a review of epidemiological and clinical studies. Maturitas 2013, 74(4):303-308. |
| 7. Clandinin MT, Wilke MS: Do trans fatty acids increase the incidence of type 2 diabetes? American Journal of Clinical Nutrition 2001, 73(6):1001-1002. |
| 8. Banz WJ, Davis JE, Clough RW, Cheatwood JL: Stearidonic acid: is there a role in the prevention and management of type 2 diabetes mellitus? J Nutr 2012, 142(3):635s-640s. |
| 9. Ye H, Wu Y, Zhuang P, Liu X, Ao Y, Li Y, Yao J, Liu H, Yang Z, Zhang Y et al: Timing of Dietary Fatty Acids to Optimize Reduced Risk of Type 2 Diabetes Mellitus: Findings from China Health and Nutrition Survey. Nutrients 2025, 17(13). |
| 10. Wilding JPH: The importance of free fatty acids in the development of Type 2 diabetes. Diabetic Medicine 2007, 24(9):934-945. |
| 11. Jeppesen C, Schiller K, Schulze MB: Omega-3 and omega-6 fatty acids and type 2 diabetes. Current Diabetes Reports 2013, 13(2):279-288. |
| 12. Xie C, Qi C, Zhang J, Wang W, Meng X, Aikepaer A, Lin Y, Su C, Liu Y, Feng X et al: When short-chain fatty acids meet type 2 diabetes mellitus: Revealing mechanisms, envisioning therapies. Biochem Pharmacol 2025, 233:116791. |
| 13. Sivri D, Akdevelioğlu Y: Effect of Fatty Acids on Glucose Metabolism and Type 2 Diabetes. Nutr Rev 2025, 83(5):897-907. |
| 14. Wang Y, Yan F, Chen Q, Liu F, Xu B, Liu Y, Huo G, Xu J, Li B, Wang S: High-fat diet promotes type 2 diabetes mellitus by disrupting gut microbial rhythms and short-chain fatty acid synthesis. Food Funct 2024, 15(21):10838-10852. |
| 15. Bradley CA: Diabetes: Omega-6 PUFAs and T2DM. Nat Rev Endocrinol 2017, 13(12):689. |
| 16. Rylander C, Sandanger TM, Engeset D, Lund E: Consumption of lean fish reduces the risk of type 2 diabetes mellitus: a prospective population based cohort study of Norwegian women. PLoS One 2014, 9(2):e89845. |
| 17. Brayner B, Kaur G, Keske MA, Livingstone KM: FADS Polymorphism, Omega-3 Fatty Acids and Diabetes Risk: A Systematic Review. Nutrients 2018, 10(6). |
| 18. Mirmiran P, Esfandyari S, Moghadam SK, Bahadoran Z, Azizi F: Fatty acid quality and quantity of diet and risk of type 2 diabetes in adults: Tehran Lipid and Glucose Study. J Diabetes Complications 2018, 32(7):655-659. |
